# PRMT3‐Mediated H4R3me2a Promotes Primary Age‐Related Tauopathy by Driving Tau Hyperphosphorylation in Neuron

**DOI:** 10.1002/advs.202506044

**Published:** 2025-05-08

**Authors:** Haotian Liu, Xinnan Liu, Fengyuan Tian, Yashuang Chen, Jingying Li, Xue Wang, Wenying Qiu, Xia Wang, Chao Ma, Wei Ge

**Affiliations:** ^1^ Department of Immunology State Key Laboratory of Complex, Severe, and Rare Diseases Institute of Basic Medical Sciences Chinese Academy of Medical Sciences School of Basic Medicine Peking Union Medical College Beijing 100005 China; ^2^ Department of Human Anatomy Histology and Embryology Neuroscience Center National Human Brain Bank for Development and Function Institute of Basic Medical Sciences Chinese Academy of Medical Sciences School of Basic Medicine Peking Union Medical College Beijing 100005 China

**Keywords:** Primary age‐related tauopathy, Alzheimer's disease, Tau hyperphosphorylation, PRMT3, H4R3me2a, miR‐448, PI3K/AKT/GSK3β signaling

## Abstract

Primary age‐related tauopathy (PART) and Alzheimer's disease (AD) both exhibit 3R/4R hyperphosphorylated tau‐positive neurofibrillary tangles (NFTs) within the hippocampal–entorhinal system. Notably, PART patients show a higher degree of tau hyperphosphorylation in the entorhinal cortex (EC) than AD, yet the molecular mechanisms driving Aβ‐independent tau hyperphosphorylation in PART remain poorly understood. Herein, through transcriptomic profiling of postmortem EC tissues and in vitro and in vivo functional validation, the present study identifies protein arginine methyltransferase 3 (PRMT3) as a critical driver of tau hyperphosphorylation. Mechanistically, PRMT3‐mediated tau hyperphosphorylation is dependent on asymmetric dimethylation of histone H4 at arginine 3 (H4R3me2a), which upregulates miR‐448. Elevated miR‐448 specifically targets and suppresses IGF1R, leading to downstream GSK3β activation and subsequent tau hyperphosphorylation through PI3K/AKT/GSK3β signaling. Treatment with SGC707, a selective PRMT3 inhibitor, effectively reduces tau hyperphosphorylation and demonstrates therapeutic promise for PART and potentially other tauopathies. Collectively, this study defines the PRMT3/H4R3me2a/miR‐448 axis as a critical regulatory pathway in tau hyperphosphorylation within PART, underscoring the potential of PRMT3 inhibition as a targeted therapeutic strategy for tauopathies.

## Graphical abstract

This study identifies PRMT3‐mediated H4R3me2a as a critical driver of tau hyperphosphorylation in primary age‐related tauopathy. Mechanistic insights reveal that the PRMT3/H4R3me2a/miR‐448 axis suppresses IGF1R expression via epigenetic regulation, further dysregulating the PI3K/AKT/GSK3β pathway. Therapeutic potential is highlighted with PRMT3 inhibition by SGC707, reducing tau pathology and reversing neuronal dysfunction in cellular and mouse models.

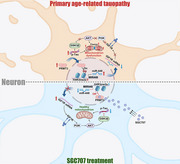

## 1 Introduction

Tau protein is predominantly expressed in neurons and plays a critical role in a variety of cellular processes, most notably in the stabilization of neuronal microtubules.^[^ ^1^ ^]^ Tauopathies, a group of neurodegenerative diseases first termed by Bernardino Ghetti and Michel Goedert, are marked by the abnormal accumulation of tau aggregates, which are composed of hyperphosphorylated tau in neurons.^[^ ^2^ ^]^ Abnormal tau hyperphosphorylation disrupts tau's binding to microtubules, reduces its solubility, and promotes its self‐aggregation.^[^ ^3^ ^]^ Moreover, hyperphosphorylated tau mislocalizes to dendritic spines, impairing synaptic function and contributing to neurodegeneration.^[^ ^4^ ^]^ Tauopathies encompass a wide range of neurodegenerative diseases, including primary tauopathies such as primary age‐related tauopathy (PART), as well as secondary tauopathies like Alzheimer's disease (AD). This makes tau‐targeted therapies potentially applicable across multiple neurodegenerative conditions. Antibody‐based immunotherapies have dominated clinical trials for tauopathies, but since tau is an endogenous protein, these approaches pose a significant risk of adverse and potentially irreversible autoimmune responses.^[^ ^5^ ^]^ Therefore, understanding the mechanisms driving tau hyperphosphorylation is crucial for developing precise therapeutic interventions, as a more refined approach beyond monoclonal antibodies may be required to effectively target tauopathies.

AD is the most prevalent and thoroughly investigated tauopathy. From a neuropathologic standpoint, AD is defined by the simultaneous presence of Aβ plaques and tau aggregates, satisfying the criteria for Alzheimer's disease neuropathologic change (ADNC).^[^ ^6^ ^]^ However, this dual pathology hinders the investigation of tau hyperphosphorylation as an independent process, thereby limiting the identification of common mechanisms across different tauopathies.

PART is particularly notable for its onset in elderly individuals and its tau pathology being limited to specific brain regions, such as the hippocampus and entorhinal cortex, which are critical for memory function.^[^ ^7^ ^]^ PART is clinically characterized by mild cognitive impairment, with a prevalence as high as 20% in individuals over the age of 80.^[^ ^8^ ^]^ Although the cognitive decline associated with PART is typically mild and often indistinguishable from normal aging, it could coexist with α‐synucleinopathies and/or TDP‐43 proteinopathies, accelerating the progression of cognitive decline.^[^ ^9^ ^]^ Without comorbidities, PART can be designated as “Definite” or “Possible” based on the extent and distribution of tau aggregates and the absence of Aβ plaques. Definite PART is defined as Braak stage I–IV in the complete absence of Aβ plaques (Thal phase “A” = 0 and CERAD neuritic plaque “C” = 0). Possible PART is diagnosed when there is a moderate level of Aβ plaques but not sufficient to meet the criteria for ADNC. Given the absence of comorbidities and Aβ plaques in Definite PART, studying this disease offers a unique opportunity to identify mechanisms that specifically drive tau hyperphosphorylation.

PART and AD share striking similarities in their tau pathology. The tau aggregates found in both PART and AD are identical and composed of paired helical hyperphosphorylated‐tau filaments containing both 3R and 4R tau isoforms,^[^ ^7^, ^10^ ^]^ which are even indistinguishable under cryo‐EM.^[^ ^11^ ^]^ The anatomical distribution and spread of tau pathology in both diseases are similar, resulting in the application of the same tau pathological staging system for both PART and AD.^[^ ^7^, ^12^ ^]^ Numerous studies have shown that Aβ pathology can exacerbate tau hyperphosphorylation,^[^ ^13^ ^]^ theoretically making tau pathology more severe in AD than in PART. However, some studies revealed that in the entorhinal cortex (EC)—where tau pathology first emerges—the level of tau phosphorylation in PART can surpass that observed in AD.^[^ ^14^ ^]^ This suggests the possible presence of a potent driver of tau phosphorylation in the EC region of PART patients, independent of Aβ‐mediated mechanisms. Therefore, describing the differences in tau phosphorylation level in the EC region between PART and AD, and utilizing omics analysis to uncover key proteins driving these differences, is crucial for advancing our understanding of tauopathy mechanisms and for identifying potential therapeutic targets.

In the present study, 41 Definite PART (referred to hereafter as PART) patients and 45 AD patients from National Human Brain Bank for Development and Function (Beijing, China) were included (Table S1, Supporting Information). We semiquantitatively assessed tau hyperphosphorylation in the EC region using AT8 immunoreactivity, finding it more severe in PART than in AD. We reclassified and analyzed the transcriptome data from our previously published studies,^[^ ^15^ ^]^ and revealed that PRMT3, a protein arginine methyltransferase, is the key protein driving PART tau hyperphosphorylation. In the EC region of PART patients, PRMT3 expression is significantly elevated, and its nuclear translocation increases, catalyzing the asymmetric dimethylation of histone H4 at arginine 3 (H4R3me2a), which is enriched at the promoter region of the MIR448 gene. This enrichment promotes the transcriptional activation of miR‐448, which in turn suppresses IGF1R expression, leading to dysfunction of the PI3K/AKT/GSK3β signaling pathway and ultimately resulting in the GSK3β activation and hyperphosphorylation of tau. In vitro and in vivo study highlighted the therapeutic efficacy of SGC707, a potent allosteric inhibitor of PRMT3, in reducing tau hyperphosphorylation and reversing cognitive deficits.

## 2 Results

### 2.1 PART Patients Present More Severe Tau Hyperphosphorylation than AD in the EC Region, Associated with Increased Expression of PRMT3

**Figure 1:**
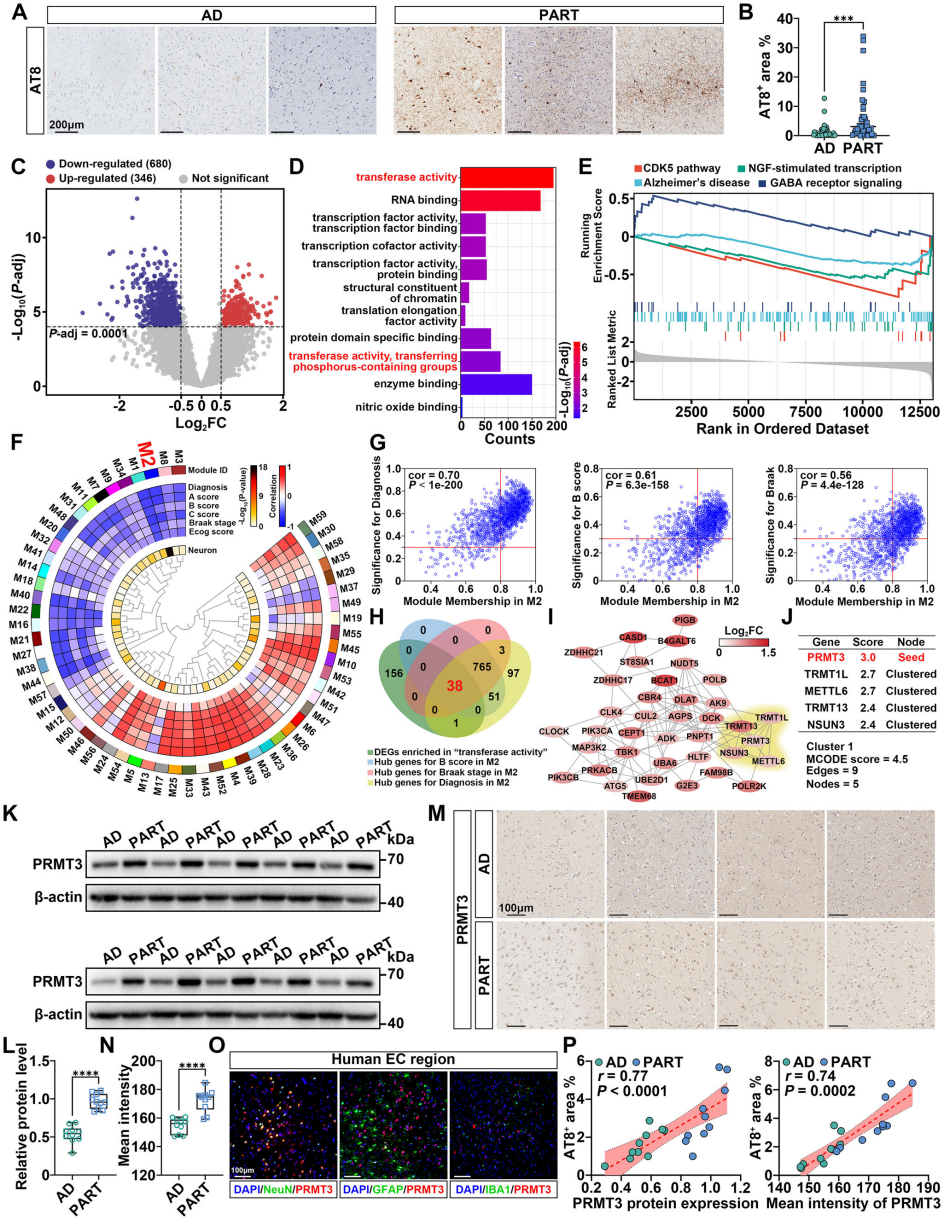
PRMT3 is increased in PART patients and correlates with tau hyperphosphorylation. A) Representative images of AT8 staining in the EC region of PART and AD patients. B) Quantification of percent area covered by AT8 staining. *n* = 43 for AD, *n* = 40 for PART. C) Volcano plots of DEGs; blue dots indicate downregulated genes and red dots indicate upregulated genes. D) Summary of DEGs Gene Ontology enrichment results. E) Summary of DEGs Gene Set Enrichment Analysis results. F) A gene co‐expression network was built using WGCNA, which consisted of 59 gene co‐expression modules. Module relatedness is shown in the central dendrogram. Module eigengenes were correlated with neuropathological and cognitive traits. The neuron nature of each module was assessed by module protein overlap with cell‐type‐specific marker lists of neurons. G) Module membership and gene significance in module M2. H) Venn diagram for identifying DEGs serving as candidate tau hyperphosphorylation driving genes in PART. I) The PPI network of DEGs visualized in Cytoscape. J) Key cluster and hub seed protein identified by MCODE plug‐in. K,L) Western blot analysis of PRMT3 in EC lysates from PART and AD patients (normalized to β‐actin). *n* = 10 for AD, *n* = 10 for PART. M,N) IHC analysis of PRMT3 in the EC region from PART and AD patients. Scale bar, 100 µm. *n* = 10 for AD, *n* = 10 for PART. O) Co‐localization of PRMT3 with NeuN, GFAP, or IBA1 in the human brain EC region. Scale bar, 100 µm. P) Correlation between PRMT3 expression and tau phosphorylation level (AT8⁺ area) in the EC region of PART and AD patients. *n* = 10 for AD, *n* = 10 for PART. Quantified data are presented as mean ± SD. Unpaired two‐tailed Student's *t* test (B, L, and N). ***p < 0.001, ****p *<* 0.0001. See also Figure S1 (Supporting Information).

To evaluate the tau phosphorylation level in the EC region of PART and AD patients, immunohistochemistry (IHC) for the p‐tau S202/T205 (AT8) were performed (*n* = 43 for AD; *n* = 40 for PART; **Figure** **1**A). We observed a significant increase in the area covered by AT8^+^ tau phosphorylation in the EC region of PART patients (Figure 1B). This finding is consistent with previous results based on smaller sample sizes.^[^ ^14^ ^]^

The transcriptome data we previously published were utilized to further screen key proteins that promote tau hyperphosphorylation in PART.^[^ ^15^ ^]^ 13 individuals with PART and 32 individuals with AD were included. The principal component analysis (PCA) plot revealed a clear separation between PART and AD samples, highlighting significant differences in their transcriptional profiles (Figure S1A, Supporting Information). We identified 1026 differentially expressed genes (DEGs; |log_2_FC| > 0.5, *P*‐adj < 0.0001) in the EC region of the PART transcriptome compared with AD (Figure 1C). Gene Ontology (GO) analysis of the 1026 DEGs revealed multiple enriched terms, including transferase activity and transcription factor activity, suggesting a role for transferase and protein transcription in PART tau pathology (Figure 1D). Gene Set Enrichment Analysis (GSEA) showed that the DEGs were significantly enriched in pathways related to neuronal function and tau phosphorylation (Figure 1E). GSEA pathway network analysis suggested that mitochondrial function might be impaired in PART (Figure S1B, Supporting Information).

To further identify potential driving proteins in PART tau pathology, weighted gene co‐expression network analysis (WGCNA) and cell type marker enrichment were performed. Specifically, sample dendrogram and trait heatmap showed that all 45 samples were included and no outlining sample was found (Figure S1C, Supporting Information). When the scale‐free fit index (signed *R* ^2^) on the ordinate approached the threshold value of 0.85 (indicated by the red line), the network was deemed close to a scale‐free distribution. Additionally, mean connectivity was near zero, identifying a soft threshold value of 10 as the optimal choice (Figure S1D, Supporting Information). We then used power 10 to calculate the adjacency and the topological overlap matrix (TOM), resulting in a clustering dendrogram with 59 gene modules (Figure S1E, Supporting Information). To investigate the relationships between these modules and tau pathology, we correlated module eigengenes with diagnosis, “ABC” score, Braak stage, and Ecog score (Figure 1F). Considering the bioinformatic analysis results of DEGs and the fact that neurons are the primary site of tau pathology, we also assessed the neuronal nature of co‐expression modules by determining if the modules were enriched in particular cell type marker proteins (Figure 1F). One particular module, M2, was identified as having the most significant neuronal characteristics and a strong tau pathology correlation. Hub genes related to diagnosis, B score, and Braak stage within the M2 module were identified based on module membership (MM > 0.8) and gene significance (GS > 0.3) (Figure 1G). We conducted a Venn analysis between these hub genes and DEGs enriched in the GO term “transferase activity.” The intersection was 38 candidate tau hyperphosphorylation driving genes in PART (Figure 1H). The 38 genes were then used to construct a protein–protein interaction (PPI) network, which was analyzed using molecular complex detection (MCODE) module (Figure 1I). The MCODE algorithm identified the highest‐scoring cluster (Score = 4.5, Edges = 9, Nodes = 5), with PRMT3 serving as the unique seed protein, indicating that PRMT3 could play a pivotal role within the network (Figure 1J). Western blot of EC tissues obtained from donors were quantitated, showing higher protein expression levels of PRMT3 in PART than AD (Figure 1K,L). IHC experiments corroborated these findings (Figure 1M,N and Figure S1F, Supporting Information). Using IF co‐staining for PRMT3 along with neuronal marker (NeuN), astrocyte marker (GFAP), or microglial marker (IBA1), it was found that PRMT3 is predominantly expressed in neurons (Figure 1O). This finding is consistent with the results of cell type enrichment analysis. To further support these findings, we performed additional analyses on publicly available single‐nucleus RNA sequencing (snRNA‐seq) datasets from the GEO database (GSE186538),^[^ ^16^ ^]^ which include samples from the hippocampus and EC—two regions profoundly affected in PART pathology. These analyses revealed that PRMT3 expression is primarily restricted to neurons, with minimal expression in other brain cell types, such as astrocytes, microglia, oligodendrocytes, and endothelial cells (Figure S1G,H, Supporting Information). These results are consistent with our findings in Figure 1F,O, further reinforcing the neuron‐specific expression of PRMT3. The correlation between AT8^+^ tau pathology and PRMT3 expression levels revealed a significant positive association between the two (Figure 1P). These results suggest that PRMT3 is likely a key driver of tau hyperphosphorylation in the EC region in PART patients.

### 2.2 PRMT3 Mediates Tau Phosphorylation In Vitro through Its Enzymatic Activity

**Figure 2:**
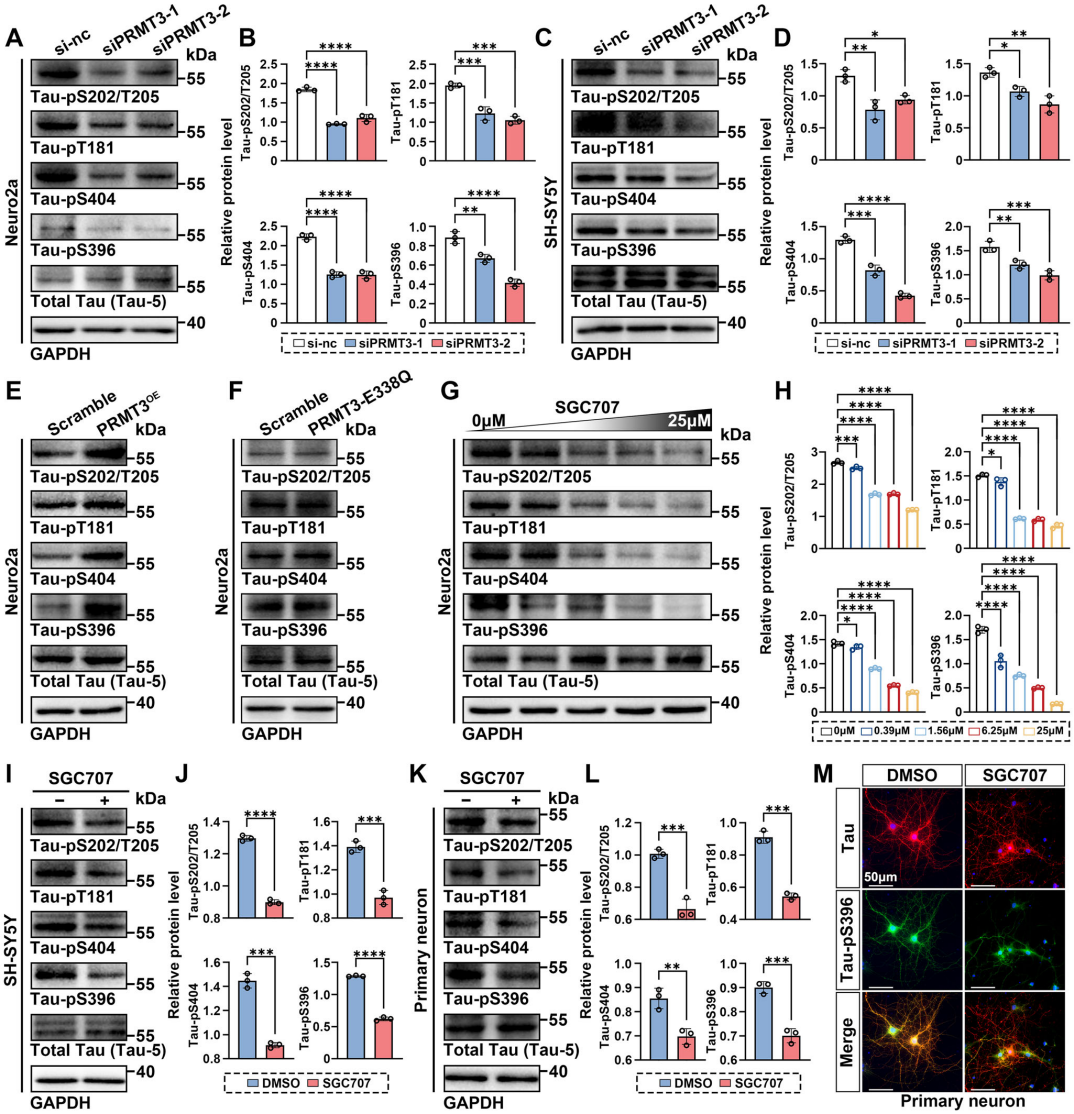
PRMT3 enzymatically promotes tau phosphorylation in vitro. A,B) Western blot and quantification of tau phosphorylation (normalized to total tau) in Neuro2a cells with PRMT3 knockdown. C,D) Western blot and quantification of tau phosphorylation (normalized to total tau) in SH‐SY5Y cells with PRMT3 knockdown. E,F) Western blot of tau phosphorylation in Neuro2a cells overexpressing PRMT3 and PRMT3‐E338Q. G,H) Western blot and quantification of tau phosphorylation (normalized to total tau) in Neuro2a cells treated with a concentration gradient of SGC707. I,J) Western blot and quantification of tau phosphorylation (normalized to total tau) in SH‐SY5Y cells treated with SGC707. K,L) Western blot and quantification of tau phosphorylation (normalized to total tau) in mouse primary neurons treated with SGC707. M) Representative images of tau (red) and tau‐pS396 (green) staining in mouse primary neurons treated with SGC707. Scale bar, 50 µm. Quantified data are presented as mean ± SD. *n* = 3 per group (B, D, H, J, and L). Unpaired Student's *t* test (J, L) or one‐way ANOVA followed by Dunnett's multiple comparisons test (B, D, and H), *p < 0.05, **p < 0.01, ***p < 0.001, ****p < 0.0001.

Tau hyperphosphorylation is a key mechanism driving tau pathology. To explore the effects of PRMT3 on tau phosphorylation, we knocked down PRMT3 in mouse neuroblastoma cell line, Neuro2a. The knockdown was confirmed by qRT‐PCR and western blot analysis (Figure S2A, Supporting Information). PRMT3 knockdown reduced tau phosphorylation at S202/T205, T181, S404, and S396 residues (**Figure** **2**A,B). This result was similarly observed with PRMT3 knockdown in human neuroblastoma cell line, SH‐SY5Y (Figure 2C,D). The knockdown efficiency was also confirmed by qRT‐PCR and western blot analysis (Figure S2B, Supporting Information). We next constructed a Neuro2a cell line stably overexpressing PRMT3 (Neuro2a‐PRMT3 cells) and measured tau phosphorylation level at S202/T205, T181, S404, and S396 residues (Figure S2C, Supporting Information). The phosphorylation levels at these sites were significantly increased in Neuro2a‐PRMT3 cells (Figure 2E and Figure S2D, Supporting Information). PRMT3 is a type I PRMT that mainly catalyzes the asymmetric dimethylation of arginine residues. To determine whether PRMT3 affects tau phosphorylation through its enzymatic activity, we constructed catalytic inactive mutant PRMT3‐E338Q. Interestingly, the catalytic inactive mutant of PRMT3 was incapable to confer such change in tau phosphorylation (Figure 2F and Figure S2E, Supporting Information). We next utilized the potent, selective, and non‐competitive PRMT3 inhibitor SGC707.^[^ ^17^ ^]^ SGC707 reduced tau phosphorylation at S202/T205, T181, S404, and S396 residues in a dose‐dependent manner, without affecting the expression levels of PRMT3 (Figure 2G,H and Figure S2F, Supporting Information). The inhibitory effect of SGC707 on tau phosphorylation was similarly observed in SH‐SY5Y cells and mouse primary neurons (Figure 2I–M and Figure S2G, Supporting Information). These results suggest that PRMT3 positively regulates tau phosphorylation levels through its enzymatic activity.

### 2.3 AAV‐Induced Neuron‐Specific PRMT3 Overexpression Exacerbates Tau Hyperphosphorylation and Cognitive Impairment in PS19 Tauopathy Mice

**Figure 3:**
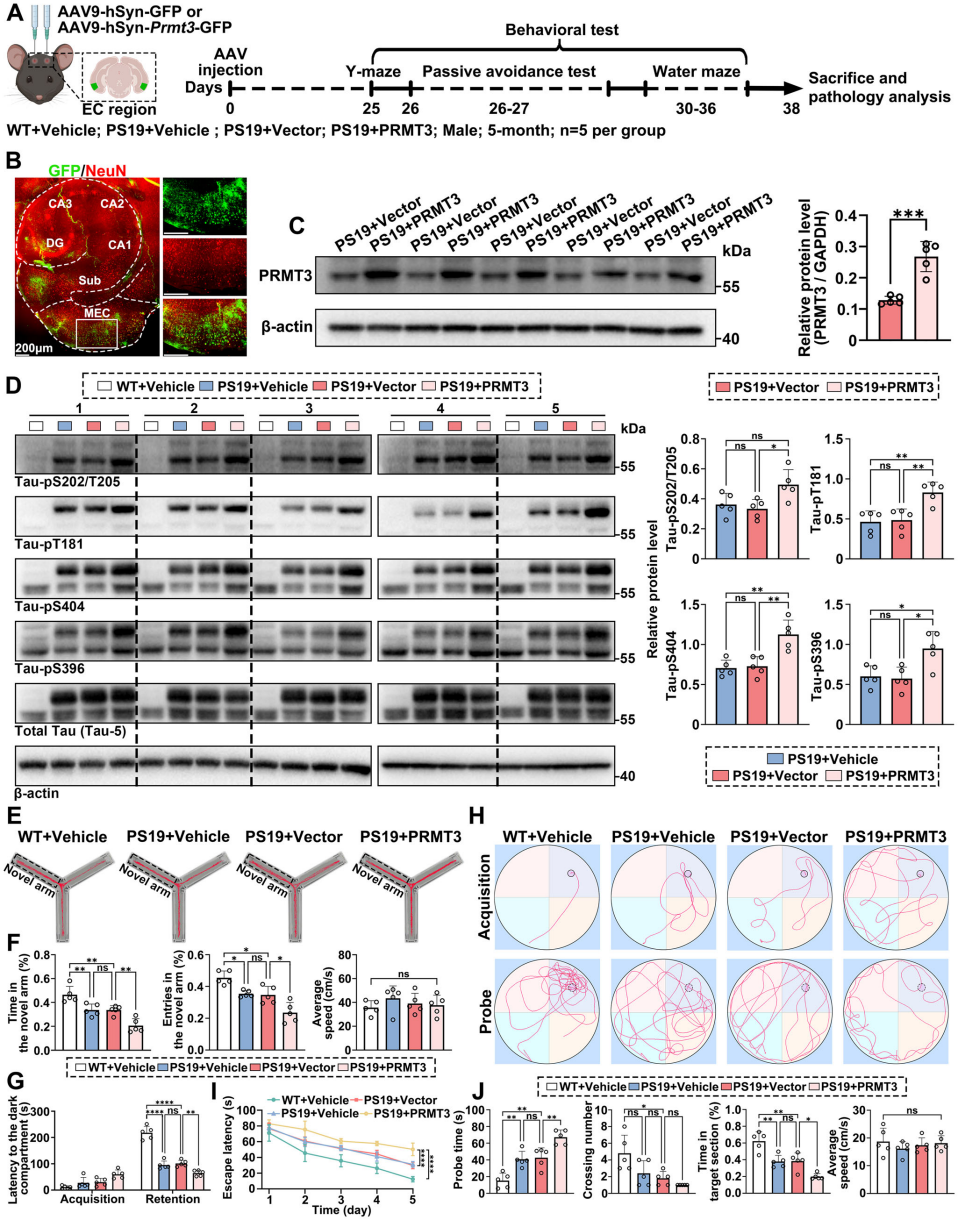
AAV‐induced neuron‐specific PRMT3 overexpression exacerbates tau hyperphosphorylation and cognitive impairment in PS19 tauopathy mice. A) Schematic of the experimental timeline for AAV treatment, behavioral testing, and pathological analysis in 5‐month‐old PS19 male mice. B) IF analysis of AAV‐induced GFP expression in the EC region. Representative images showing GFP signal colocalized with neuron. Scale bar, 200 µm. C) Western blot and quantification of PRMT3 levels in the EC region of vector‐injected or PRMT3‐injected PS19 mice. D) Western blot and quantification of tau phosphorylation (normalized to total tau) in the EC region of four PS19 mouse groups. E,F) Representative path traces and quantification of the Y‐maze test performance of four PS19 mouse groups. G) Latency to enter the dark compartment of four PS19 mouse groups in passive avoidance test. H–J) Representative swim paths and quantification of the Morris water maze test performance of four PS19 mouse groups. Quantified data are presented as mean ± SD. *n* = 5 per group. Unpaired Student's *t* test (C) or one‐way ANOVA followed by Tukey's multiple comparisons test, *p < 0.05, **p < 0.01, ***p < 0.001, ****p < 0.0001, ns = not significant.

To further investigate the in vivo impact of neuronal PRMT3 on tau phosphorylation, we employed Tau P301S transgenic mice (PS19), which overexpress human tau carrying the P301S mutation. This model was selected because tau pathology in PS19 mice is primarily localized to the hippocampus and EC—regions that closely mirror the early distribution of tau pathology observed in PART.^[^ ^7^, ^18^ ^]^ We next performed bilateral stereotaxic injections into the medial entorhinal cortex (MEC) of 5‐month‐old PS19 mice with either PBS (Vehicle), AAV9‐hSyn‐GFP (Vector), or AAV9‐hSyn‐*Prmt3*‐GFP. Age‐matched wild‐type (WT) littermates received bilateral MEC injections of PBS and served as healthy baseline controls (**Figure** **3**A). At 38 days post‐injection, IF and western blot analysis confirmed robust neuronal overexpression of PRMT3 (Figure 3B,C). Neuron‐specific PRMT3 overexpression markedly exacerbated tau phosphorylation at multiple pathological sites, including S202/T205, T181, S404, and S396 (Figure 3D). To assess cognitive function, several behavioral tests were conducted starting on day 25 post‐injection. In the Y‐maze test, mice overexpressing PRMT3 entered the novel arm less frequently and spent significantly less time in the novel arm compared to vector‐injected PS19 (Figure 3E,F). In the passive avoidance test, PRMT3 overexpression significantly impaired memory retention (Figure 3G). Consistently, in the water maze, PRMT3‐overexpressing mice exhibited impaired spatial learning and memory, as evidenced by increased latency to locate the hidden platform during the training phase and reduced time spent in the target quadrant during the probe trial, relative to vector‐injected PS19 (Figure 3H–J). Together, these findings demonstrate that neuronal overexpression of PRMT3 in the EC region aggravates tau hyperphosphorylation and exacerbates cognitive deficits in PS19 tauopathy mice.

### 2.4 PRMT3‐Mediated H4R3me2a Is Strongly Associated with Tau Hyperphosphorylation in PART

**Figure 4:**
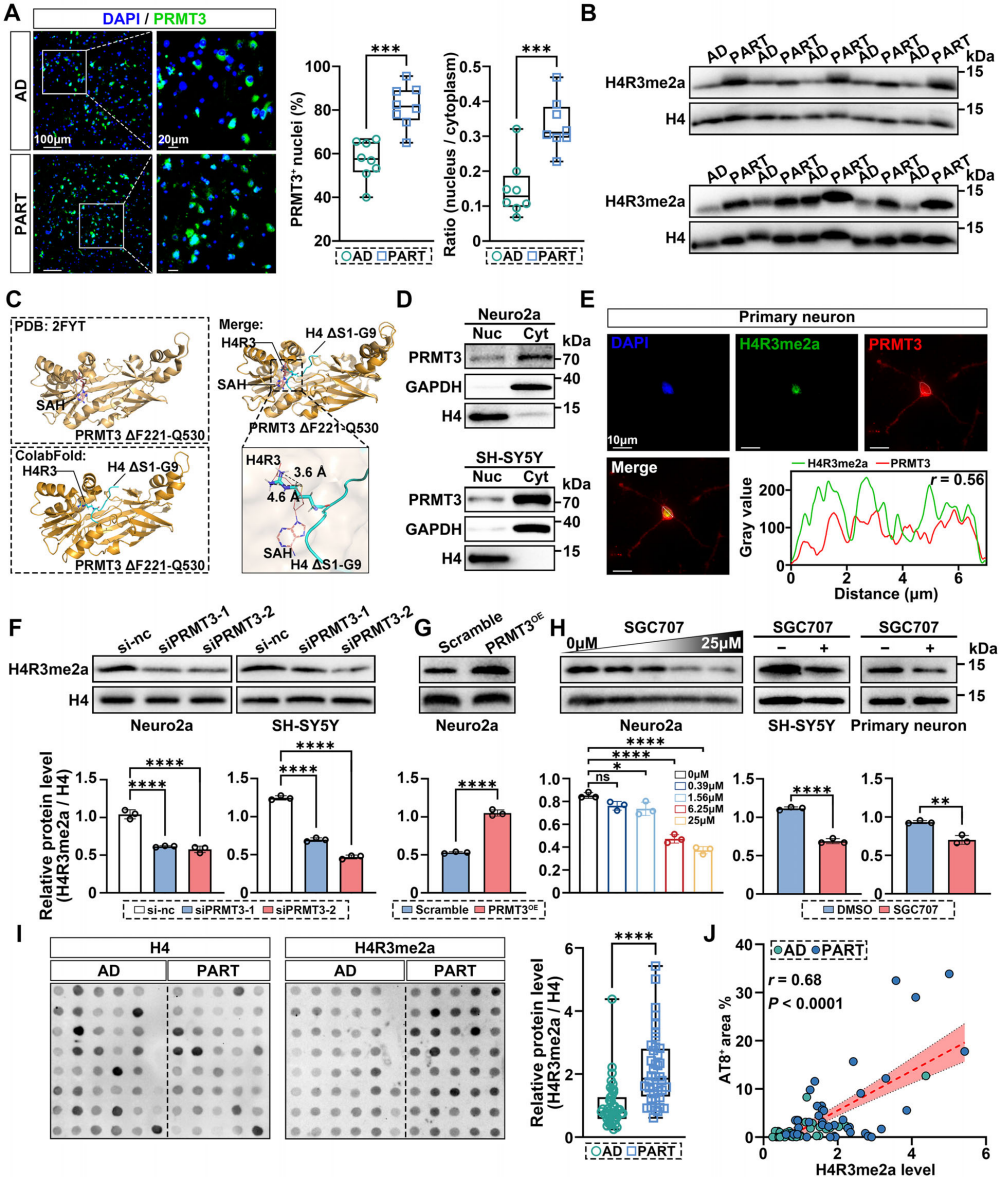
PRMT3‐mediated H4R3me2a is strongly associated with tau hyperphosphorylation in PART. A) IF analysis of PRMT3 in the EC region of PART and AD patients. Scale bar, 100, 20 µm. B) Western blot analysis of H4R3me2a and H4 in EC lysates from PART and AD patients. C) Structural analysis of PRMT3 and H4 peptide interaction using molecular docking and simulation. D) Nuclear and cytoplasmic localization of PRMT3 in Neuro2a and SH‐SY5Y cells. E) Nuclear colocalization of PRMT3 and H4R3me2a in primary neurons. Scale bar, 10 µm. F) Western blot and quantification of H4R3me2a (normalized to H4) in Neuro2a cells and SH‐SY5Y cells with PRMT3 knockdown. G) Western blot and quantification of H4R3me2a (normalized to H4) in Neuro2a cells overexpressing PRMT3. H) Western blot and quantification of H4R3me2a (normalized to H4) in SGC707‐treated Neuro2a, SH‐SY5Y cells, and primary neurons. I) Dot blot and quantification of H4R3me2a (normalized to H4) in EC lysates from PART and AD patients. *n* = 43 for AD, *n* = 40 for PART. J) Correlation between H4R3me2a level and tau phosphorylation level (AT8⁺ area) in the EC region of PART and AD patients. *n* = 43 for AD, *n* = 40 for PART. Quantified data are presented as mean ± SD. *n* = 8 per group (A), *n* = 3 per group (F–H). Unpaired two‐tailed Student's *t* test (A, F, G, H, and I) or one‐way ANOVA followed by Dunnett's multiple comparisons test (H). **p < 0.01, ***p < 0.001, ****p < 0.0001.

We next investigated the molecular mechanisms by which neuronal PRMT3 promotes tau phosphorylation. Under physiological conditions, PRMT3 is predominantly localized in the cytoplasm.^[^ ^19^ ^]^ However, in the EC region of the PART brain, we observed an increased number of neurons with nuclear PRMT3 expression and an increased expression level of PRMT3 within the nucleus, suggesting that PRMT3 may catalyze enhanced modification of nuclear substrates (**Figure** **4**A). PRMT3 has been reported to catalyze the asymmetric dimethylation of histone H4 at arginine 3 (H4R3).^[^ ^17^ ^]^ Western blot analysis showed that the level of H4R3me2a was significantly higher in PART samples compared to AD (Figure 4B and Figure S3A, Supporting Information). In addition to PRMT3, PRMT1, and PRMT6 are also capable of catalyzing H4R3me2a.^[^ ^20^ ^]^ To confirm that the high level of H4R3me2a is due to PRMT3, we first analyzed the transcriptome data for the expression levels of PRMT1, PRMT3, and PRMT6. Only PRMT3 was upregulated in the EC region of the PART brain (Figure S3B, Supporting Information). Based on cell type analysis of snRNA‐seq data from the human EC region (GEO dataset GSE186538),^[^ ^16^ ^]^ it was further found that PRMT3‐positive neurons were the most abundant and had the highest expression levels in neurons compared to PRMT1 and PRMT6 (Figure S3C, Supporting Information). Western blot assay of PART and AD with Pearson's correlation test of gray values revealed a significant positive correlation between PRMT3 expression and H4R3me2a level (Figure S3D, Supporting Information). These results highlight the significant role of PRMT3‐mediated H4R3me2a in PART.

In this study, we used ColabFold to simulate the docking of the PRMT3 MTase domain and H4 peptide (1–9). The distance between the methyl group of the donor SAM (S‐adenosylmethionine) and the methyl acceptor site on H4R3 is 3.6 and 4.6 Å, respectively, further corroborating previous research findings that PRMT3 catalyzes H4R3me2a from a structural perspective (Figure 4C). Nuclear‐cytoplasmic fractionation and IF experiments confirmed that PRMT3 is also expressed in the nuclei of Neuro2a, SH‐SY5Y cells, and primary neurons, and that it colocalizes with H4R3me2a (Figure 4D,E). Knockdown of PRMT3 in Neuro2a and SH‐SY5Y cells resulted in a significant decrease of H4R3me2a (Figure 4F). Conversely, in Neuro2a‐PRMT3 cells, H4R3me2a was significantly elevated (Figure 4G), whilst the catalytic inactive mutant of PRMT3 did not exhibit this effect (Figure S3E, Supporting Information). Following stimulation with SGC707, H4R3me2a in Neuro2a, SH‐SY5Y, and primary neurons decreased in a dose‐dependent manner (Figure 4H). We also observed increased levels of H4R3me2a in PS19 mice with AAV‐induced, neuron‐specific PRMT3 overexpression compared to vector‐injected controls (Figure S3F, Supporting Information). To clarify whether PRMT3 regulates tau phosphorylation through its impact on H4R3me2a, we conducted a dot blot analysis of H4R3me2a in the EC region of 45 PART and 41 AD patients. The results of Ponceau S staining demonstrated the consistency of sample loading in the dot blot assay and validated the reliability of the dot blot results (Figure S3G, Supporting Information). Correlation analysis with AT8 tau pathology revealed higher H4R3me2a levels in the EC region of PART patients compared to AD, with a positive correlation to tau phosphorylation level, suggesting that PRMT3‐mediated H4R3me2a is strongly associated with tau hyperphosphorylation in PART (Figure 4I,J).

### 2.5 H4R3me2a Facilitates Tau Hyperphosphorylation via the miR‐448/IGF1R/AKT/GSK3β Axis

**Figure 5:**
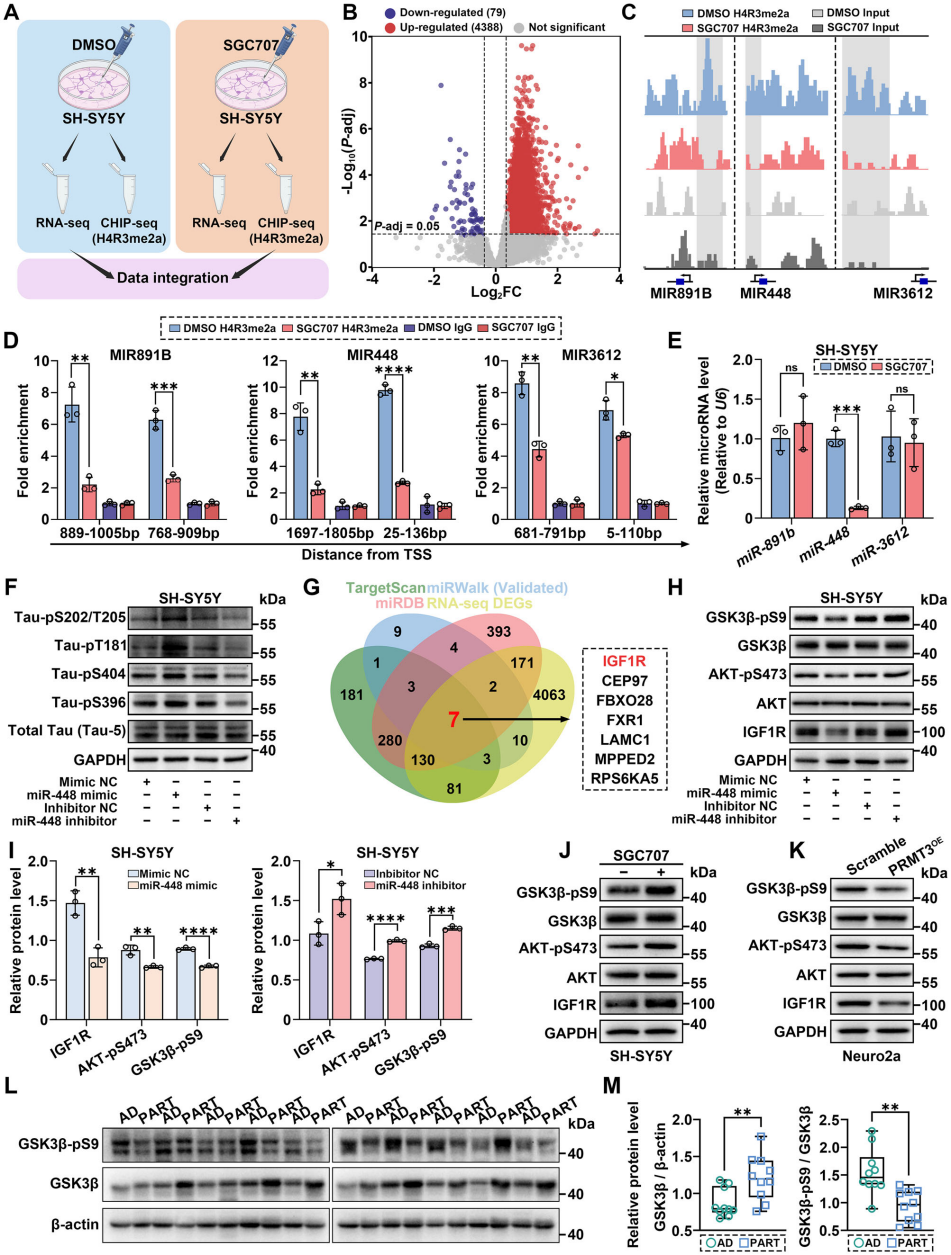
PRMT3‐mediated H4R3me2a drives tau hyperphosphorylation via miR‐448/IGF1R/AKT/GSK3β axis. A) Schematic of the experimental design. B) Volcano plot showing the DEGs between DMSO‐ and SGC707‐treated SH‐SY5Y cells, highlighting significantly down‐regulated (blue) and up‐regulated (red) genes. C) ChIP‐seq profiles for H4R3me2a at the promoters of MIR891B, MIR448, and MIR3612 in SGC707‐treated SH‐SY5Y cells. D) ChIP‐qPCR quantification of H4R3me2a enrichment at the promoter regions of MIR891B, MIR448, and MIR3612 under SGC707 conditions. E) qPCR analysis of miR‐891B, miR‐448, and miR‐3612 expression levels (normalized to U6) in SH‐SY5Y cells treated with SGC707. F) Western blot analysis of tau phosphorylation in SH‐SY5Y cells transfected with miR‐448 mimic or inhibitor. G) Venn diagram showing common targets of miR‐448 identified through TargetScan, miRWalk, miRDB, and RNA‐seq data, with IGF1R as a potential target. H,I) Western blot and quantification of IGF1R (normalized to GAPDH), AKT‐pS473 (normalized to AKT), and GSK3β‐pS9 (normalized to GSK3β) levels in SH‐SY5Y cells transfected with miR‐448 mimic or inhibitor. J) Western blot analysis of IGF1R, AKT, AKT‐pS473, GSK3β, and GSK3β‐pS9 in SGC707‐treated SH‐SY5Y cells. K) Western blot analysis of IGF1R, AKT, AKT‐pS473, GSK3β, and GSK3β‐pS9 in Neuro2a cells overexpressing PRMT3. L,M) Western blot and quantification of GSK3β‐pS9 (normalized to GSK3β) and total GSK3β (normalized to β‐actin) in EC lysates from PART and AD patients. *n* = 10 for AD, *n* = 10 for PART. Quantified data are presented as mean ± SD. *n* = 3 per group (D, E, and I). Unpaired two‐tailed Student's *t* test (D, E, I, and M). *p < 0.05, **p *<* 0.01, ***p < 0.001, ****p < 0.0001, ns =not significant.

To investigate the molecular mechanism by which H4R3me2a promote tau phosphorylation, we performed RNA‐seq and ChIP‐seq to identify the H4R3me2a‐binding genes that may regulate tau phosphorylation in SGC707‐treated SH‐SY5Y cells (**Figure** **5**A). Theoretically, the reduction in H4R3me2a levels caused by SGC707 should lead to the downregulation of a large number of genes, as H4R3me2a is generally considered a mark associated with transcriptional activation.^[^ ^21^ ^]^ However, surprisingly, RNA‐seq data revealed that 4,388 genes were upregulated, while 79 genes were downregulated following SGC707 treatment (Table S2, Supporting Information). This suggests that these gene expression changes may be indirectly regulated by H4R3me2a (Figure 5B). Kyoto encyclopedia of genes and genomes pathway analysis revealed that these DEGs were significantly enriched in gene sets associated with aging and neurodegenerative diseases where tau hyperphosphorylation is observed (Figure S4A, Supporting Information). Subsequently, we performed H4R3me2a ChIP‐seq. Correlation analysis revealed a significant difference between ChIP products and input DNA, confirming the credibility of the ChIP‐seq data, while the divergence between the SGC707 treatment and DMSO control highlights the specific effects of SGC707 on the genomic distribution of H4R3me2a (Figure S4B, Supporting Information). A total of 14 040 H4R3me2a‐binding peaks were identified, corresponding to 6978 enriched genes, of which 250 protein‐coding genes were localized to promoter regions. Comparing H4R3me2a binding before and after SGC707 treatment, we identified 5881 differential peaks with a |log_2_FC| > 0.5 threshold, which were associated with 4070 enriched genes. Notably, only 110 protein‐coding genes (≈2.7% of the total differential genes) were localized to promoter regions (Table S3, Supporting Information). Importantly, these 110 genes showed no overlap with the downregulated genes identified in the RNA‐seq data, suggesting that the regulation of gene expression by H4R3me2a occurs predominantly through indirect mechanisms (Figure S4C, Supporting Information). Histone methylation can regulate the expression of microRNAs.^[^ ^22^ ^]^ PRMT3 was reported to activate the expression of miR‐3648 by enhancing H4R3me2a levels at promoter region of the gene.^[^ ^23^ ^]^ Therefore, we hypothesized that SGC707 reduces H4R3me2a levels at the promoter regions of microRNAs, leading to a decrease in microRNA expression, which in turn results in the upregulation of their target mRNAs. Our study identified H4R3me2a enrichment at the promoter regions of MIR891B, MIR448, and MIR3612, with a significant reduction in H4R3me2a levels at these loci following SGC707 treatment (Figure 5C). This result was further validated by ChIP‐qPCR (Figure 5D). We detected the expression of these three microRNAs following SGC707 treatment and observed a significant downregulation of miR‐448 (Figure 5E). For the three microRNAs analyzed, only miR‐448 showed a decrease in expression corresponding to the reduction in H4R3me2a levels. We hypothesize that the expression of the other two microRNAs is maintained by alternative transcriptional activation mechanisms. For instance, the reduction in H4R3me2a could be compensated by other activating histone modifications, such as H3K4me3 or H3K27ac, thereby preserving their expression. Alternatively, these two microRNAs may rely on enhancer‐mediated regulation rather than promoter‐associated H4R3me2a modifications. Furthermore, in Neuro2a cells treated with SGC707, we observed a significant downregulation of miR‐448 (referred to as miR‐448‐3p in *Mus musculus*), whereas overexpression of PRMT3 in Neuro2a cells resulted in the opposite effect (Figure S4D,E). These results suggest that PRMT3 catalyzes H4R3me2a formation, leading to elevated H4R3me2a levels at the MIR448 promoter and subsequent activation of its expression.

To further investigate the functional role of miR‐448 in tau hyperphosphorylation, we modulated miR‐448 expression in SH‐SY5Y cells by overexpressing it with a miR‐448 mimic and inhibiting it with a miR‐448 inhibitor. The results showed that the miR‐448 mimic significantly increased tau phosphorylation, while the miR‐448 inhibitor led to a marked reduction in tau phosphorylation (Figure 5F and Figure S4F, Supporting Information). To identify the downstream targets of miR‐448 mediating its regulatory effects on tau phosphorylation, we compiled the predicted targets of miR‐448 using bioinformatic tools, including TargetScan, MiRWalk, and miRDB. These predicted targets were then overlapped with the DEGs from our RNA‐seq data, resulting in the identification of 7 genes (Figure 5G). Among these, IGF1R has been reported to be suppressed by miR‐448 through binding to an 8‐mer site within the 3′ untranslated region (3′UTR) of *IGF1R* mRNA, a mechanism validated by dual‐luciferase reporter assays and RNA pull‐down experiments.^[^ ^24^ ^]^ And it has also been shown to regulate tau phosphorylation, potentially through the activation of the PI3K/AKT/GSK3β signaling.^[^ ^25^ ^]^ Western blot analysis revealed that overexpression of miR‐448 in SH‐SY5Y cells significantly reduced the protein levels of IGF1R, AKT‐pS473, and GSK3β‐pS9, whereas inhibition of miR‐448 led to a marked increase in these proteins (Figure 5H,I). Treatment with SGC707 in SH‐SY5Y cells resulted in a significant upregulation of IGF1R at both the mRNA and protein levels, accompanied by the activation of the PI3K/AKT/GSK3β signaling pathway (Figure 5J and Figure S4G,H, Supporting Information). In contrast, in Neuro2a‐PRMT3 cells, IGF1R expression was reduced at both mRNA and protein levels, and PI3K/AKT/GSK3β signaling was suppressed (Figure 5K and Figure S4I,J, Supporting Information). We also observed a significant upregulation of miR‐448 in the EC region of PS19 mice with AAV‐induced neuron‐specific PRMT3 overexpression, accompanied by reduced IGF1R expression and suppression of the PI3K/AKT/GSK3β signaling pathway (Figure S4K–M, Supporting Information). In the EC region of PART patients, we observed an increase in GSK3β expression and a decrease in phosphorylated GSK3β at Ser9 (Figure 5L,M). Furthermore, miR‐448 was significantly upregulated in the EC region of PART patients compared to controls (Figure S4N, Supporting Information), likely reflecting its neuron‐specific expression, whereas no significant changes in IGF1R expression were detected, potentially due to the confounding and compensatory effects of non‐neuronal cell populations in bulk tissue analysis (Figure S4O, Supporting Information). These findings suggest that in the EC neurons of PART patients, PRMT3 elevates H4R3me2a levels at the promoter region of MIR448, thereby promoting miR‐448 expression. This leads to a reduction in the expression of its target gene, IGF1R, which, through the PI3K/AKT/GSK3β pathway, reduces GSK3β‐pS9 levels, enhancing GSK3β activity and ultimately resulting in tau hyperphosphorylation.

Based on the experimental findings, we propose that the PRMT3/H4R3me2a/miR‐448/IGF1R/PI3K/AKT/GSK3β axis plays a pivotal role in regulating tau phosphorylation. Although this signaling pathway is extensive and may raise concerns about specificity—given that PRMT3 methylates substrates beyond H4R3, miR‐448 targets multiple genes, and IGF1R could influence tau phosphorylation through alternative pathways—our results underscore its specificity and functional relevance. To validate the pathway's specificity, we first treated Neuro2a and SH‐SY5Y cells with SGC707, that broadly impacts asymmetric dimethylation of all PRMT3 substrates. On this background, transfection of the miR‐448 mimic significantly attenuated SGC707's protective effect against tau hyperphosphorylation (Figure S5A,B, Supporting Information). This finding suggests that PRMT3 modulates tau phosphorylation primarily through the H4R3me2a/miR‐448 axis rather than other substrates. Next, we analyzed the expression of the other six predicted miR‐448 target genes identified through the intersection of predicted targets and DEGs (Figure 5G). Upon miR‐448 mimic transfection in SH‐SY5Y cells, all six genes showed downregulation at the mRNA level, with *CEP97*, *FBXO28*, and *RPS6KA5* exhibiting significant reductions (Figure S5C, Supporting Information). However, knockdown of FBXO28 or RPS6KA5, which have potential roles in extensive cellular regulatory pathways, did not lead to significant changes in tau phosphorylation levels (Figure S5D,E, Supporting Information). We then generated Neuro2a cells overexpressing IGF1R and treated them with the miR‐448 mimic. Overexpression of IGF1R rescued the miR‐448‐induced increase in tau phosphorylation (Figure S5F, Supporting Information), further supporting the role of miR‐448 in regulating tau phosphorylation primarily through IGF1R rather than other targets. Lastly, we co‐treated SH‐SY5Y cells with the AKT agonist SC79 and the miR‐448 mimic. Activation of the AKT pathway by SC79 rescued the miR‐448‐induced inhibition of tau phosphorylation, indicating that IGF1R influences tau phosphorylation through the PI3K/AKT/GSK3β signaling cascade (Figure S5G, Supporting Information). In summary, our findings highlight the specificity of the mechanism by which PRMT3 regulates tau phosphorylation through the H4R3me2a/miR‐448/IGF1R/PI3K/AKT/GSK3β axis.

### 2.6 PRMT3 Inhibition Attenuates Tau Hyperphosphorylation and Reverses Resulting Neuron Damage and Mitochondrial Dysfunction in Tauopathy Cell Models

**Figure 6:**
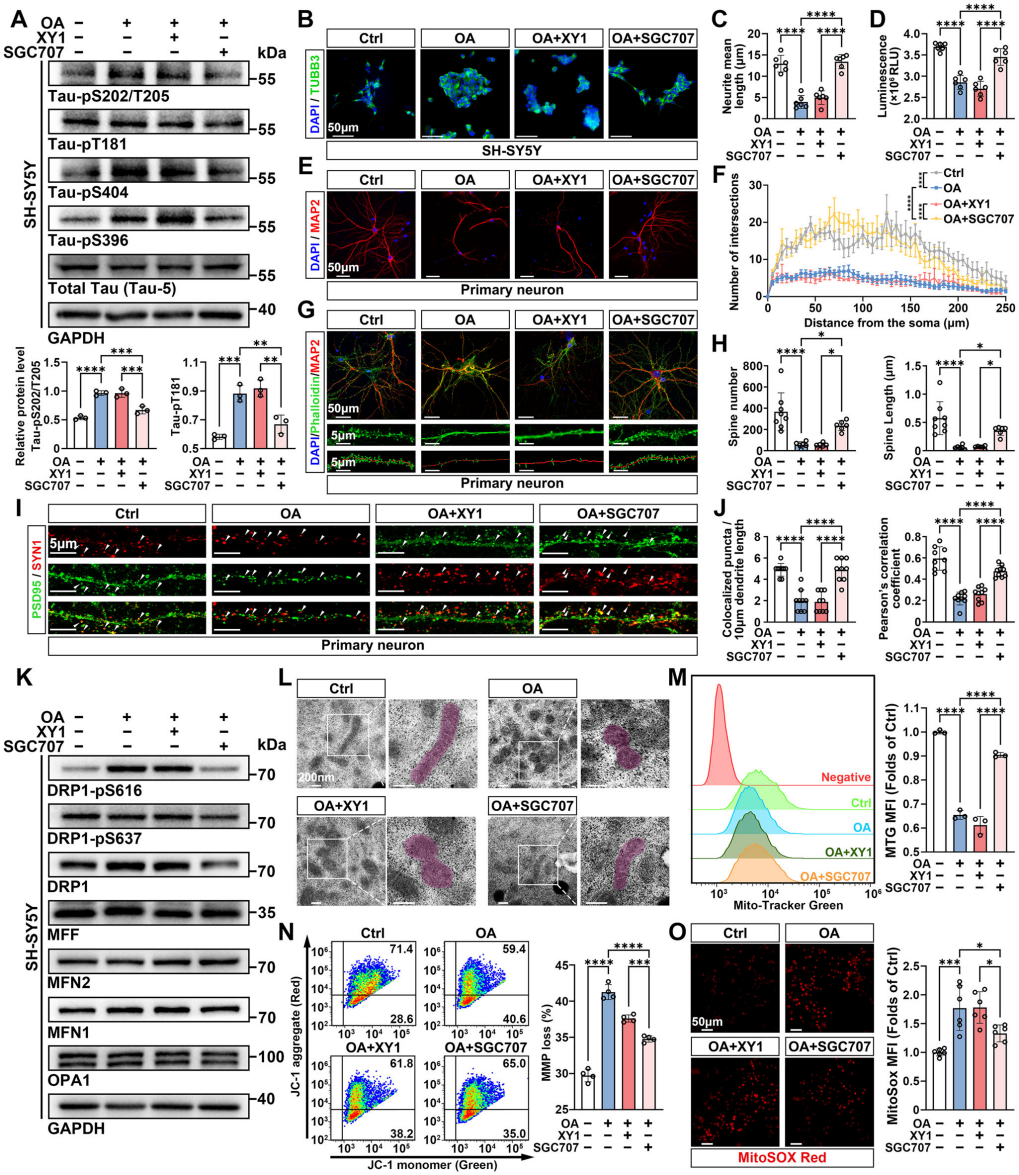
PRMT3 inhibition attenuates tau hyperphosphorylation and reverses resulting neuron damage and mitochondrial dysfunction in tauopathy cell models. A) Western blot and quantification of tau phosphorylation (normalized to total tau) in tauopathy SH‐SY5Y cells treated with SGC707 or XY1. *n* = 3 per group. B,C) IF analysis of neurites in tauopathy SH‐SY5Y cells treated with SGC707 or XY1. *n* = 6 per group. D) Cell viability analysis of tauopathy SH‐SY5Y cells treated with SGC707 or XY1. *n* = 6 per group. E) Representative images of MAP2 (red) staining in tauopathy primary neurons treated with SGC707 or XY1. Scale bar, 50 µm. F) Sholl analysis of dendritic complexity. *n* = 5–9 per group. G) Representative images of MAP2 (red) and Phalloidin (green) co‐staining in tauopathy primary neurons treated with SGC707 or XY1. Scale bar, 50, 5 µm. H) Dendritic spine density and morphology analysis in tauopathy primary neurons treated with SGC707 or XY1. *n* = 6–8 per group. I) High‐resolution confocal imaging of SYN1 (red) and PSD95 (green) synapses puncta in tauopathy primary neurons treated with SGC707 or XY1. Scale bar, 5 µm. J) Quantification of colocalized synapses puncta and Pearson correlation coefficients. *n* = 9 per group. K) Western blot analysis of mitochondrial dynamics proteins (DRP1, MFF, MFN2, MFN1, OPA1) and DRP1 phosphorylation at pS616 and pS637 in tauopathy SH‐SY5Y cells treated with SGC707 or XY1. L) Representative TEM images of mitochondrial morphology in tauopathy SH‐SY5Y cells treated with SGC707 or XY1. Scale bar, 200 nm. M) Quantification of mitochondrial mass using MitoTracker Green in tauopathy SH‐SY5Y cells treated with SGC707 or XY1. *n* = 3 per group. N) JC‐1 staining and flow cytometry analysis of mitochondrial membrane potential in tauopathy SH‐SY5Y cells treated with SGC707 or XY1. *n* = 4 per group. O) MitoSOX Red staining of mitochondrial superoxide levels in tauopathy SH‐SY5Y cells treated with SGC707 or XY1. *n* = 6 per group. Scale bar, 50 µm. Quantified data are presented as mean ± SD. One‐way ANOVA followed by Tukey's multiple comparisons test, two‐way ANOVA followed by Tukey's multiple comparisons test (F). *p < 0.05, **p *< *0.01, ***p < 0.001, ****p < 0.0001.

To further explore whether PRMT3 inhibition has potential for treating tau hyperphosphorylation and the resulting neuronal dysfunction, we used PRMT3 specific inhibitor SGC707 to treat in vitro tauopathy cell models induced by okadaic acid (OA). XY‐1, a close analogue of SGC707 that is completely inactive against PRMT3, was used as a negative control. After induction by OA, SH‐SY5Y cells exhibited tau hyperphosphorylation at S202/T205, T181, S404, and S396 residues. Following treatment with SGC707, the phosphorylation levels at these sites significantly decreased (**Figures** **6**A and S6A, Supporting Information). Furthermore, SGC707 significantly downregulated the expression of H4R3me2a and miR‐448, while markedly activating IGF1R/PI3K/AKT/GSK3β axis, whereas XY1 showed no such effect (Figure S6B–D, Supporting Information). Altered neuronal morphology is associated with functional changes during aging and neurodegenerative disease.^[^ ^15a^ ^]^ Thus, we further analyzed cell morphology and viability to assess the degree of damage induced by OA and the restorative effects of SGC707. In terms of cell morphology, after induction by OA, cell bodies adopted a round shape and neurites were shortened. The cell morphology in the SGC707 treatment group was restored and maintained (Figure 6B,C). In addition, SH‐SY5Y cells had higher viability after SGC707 treatment (Figure 6D). To assess the more detailed functional improvements of SGC707 on tauopathy neurons, OA‐induced primary tauopathy neurons were constructed. We first detected morphological changes in the primary tauopathy neurons after SGC707 treatment; MAP2 signals were used to outline neuronal morphology (Figure 6E). We found that SGC707 treatment restored the intersection number in the Sholl analysis (Figure 6F). The dendrite length, area, and branch number were also increased after SGC707 treatment (Figure S6E, Supporting Information). Dendritic spines are small, membranous protrusions found on the dendrites of neurons. They play a critical role in synaptic transmission and plasticity, serving as the postsynaptic site for most excitatory synapses in the brain.^[^ ^26^ ^]^ In this study, we observed significant dendritic spine loss in OA‐induced tauopathy neurons. SGC707 treatment significantly increased spine numbers, while XY1 had no effect (Figure 6G). Additionally, spine length and volume all recovered after SGC707 treatment, indicating improved dendritic spine morphology and potentially restored synaptic function (Figure 6H and Figure S6F, Supporting Information). Thus, we further assessed whether SGC707 had an effect on functional synapses in vitro. Co‐staining of the presynaptic membrane (SYN1) and the postsynaptic membrane (PSD95) revealed that SGC707 significantly alleviated OA‐induced synaptic loss, whereas XY1 had no such effect (Figure 6I,J).

Hyperphosphorylated tau can impair mitochondrial dynamics by regulating mitochondrial fission/fusion proteins, leading to mitochondrial dysfunction and neuronal damage.^[^ ^27^ ^]^ Moreover, the PI3K/AKT/GSK3β pathway plays a crucial role in maintaining mitochondrial function.^[^ ^28^ ^]^ Hence, we hypothesized that SGC707 could restore the neuron mitochondrial function and maintain the mitochondrial dynamic balance. In this study, we observed an upregulation of the mitochondrial fission protein DRP1 in the SH‐SY5Y tauopathy model, while the levels of fusion proteins MFN1, MFN2, and OPA1 remained unchanged. Additionally, we detected an increase in DRP1 phosphorylation at Ser616, which promotes mitochondrial fission, accompanied by a decrease in phosphorylation at Ser637, a modification that inhibits DRP1 activity. Following treatment with SGC707, DRP1 expression was downregulated, and phosphorylation of DRP1 at Ser616 was reduced, indicating a restoration of mitochondrial dynamics (Figure 6K and Figure S6G, Supporting Information). To further validate these changes in mitochondrial morphology, we employed transmission electron microscopy (TEM) to confirm the alterations in mitochondrial fission and fusion (Figure 6L). These findings suggest that in the context of tau hyperphosphorylation, mitochondrial dynamics shift toward increased fission, and treatment with SGC707 effectively reverses this shift.

Next, we investigated the effects of SGC707 on mitochondrial function. Mitochondrial mass, as indicated by the mean fluorescence intensity (MFI) of MitoTracker‐Green (MTG), showed a significant decrease in the SH‐SY5Y tauopathy model, which was markedly restored following SGC707 treatment (Figure 6M). We subsequently used JC‐1 to label SH‐SY5Y cells for testing the mitochondrial membrane potential (MMP). Flow cytometry analysis revealed that the MMP of SH‐SY5Y cells were significantly reduced in the OA group, while the MMP in the SGC707‐treated group achieved partial recovery (Figure 6N). Mitochondria are responsible for producing the energy required by cells through the process of oxidative phosphorylation, which is also accompanied by the generation of reactive oxygen species (ROS). We observed a significant accumulation of ROS in OA‐stimulated SH‐SY5Y cells, which was markedly alleviated following SGC707 treatment (Figure 6O). These results indicate that SGC707 effectively recover and rescue OA‐induced mitochondrial dysfunction in SH‐SY5Y cells.

### 2.7 PRMT3 Inhibition Suppresses Tau Pathology and Attenuates Cognitive Deficits in PS19 Tauopathy Mice

**Figure 7:**
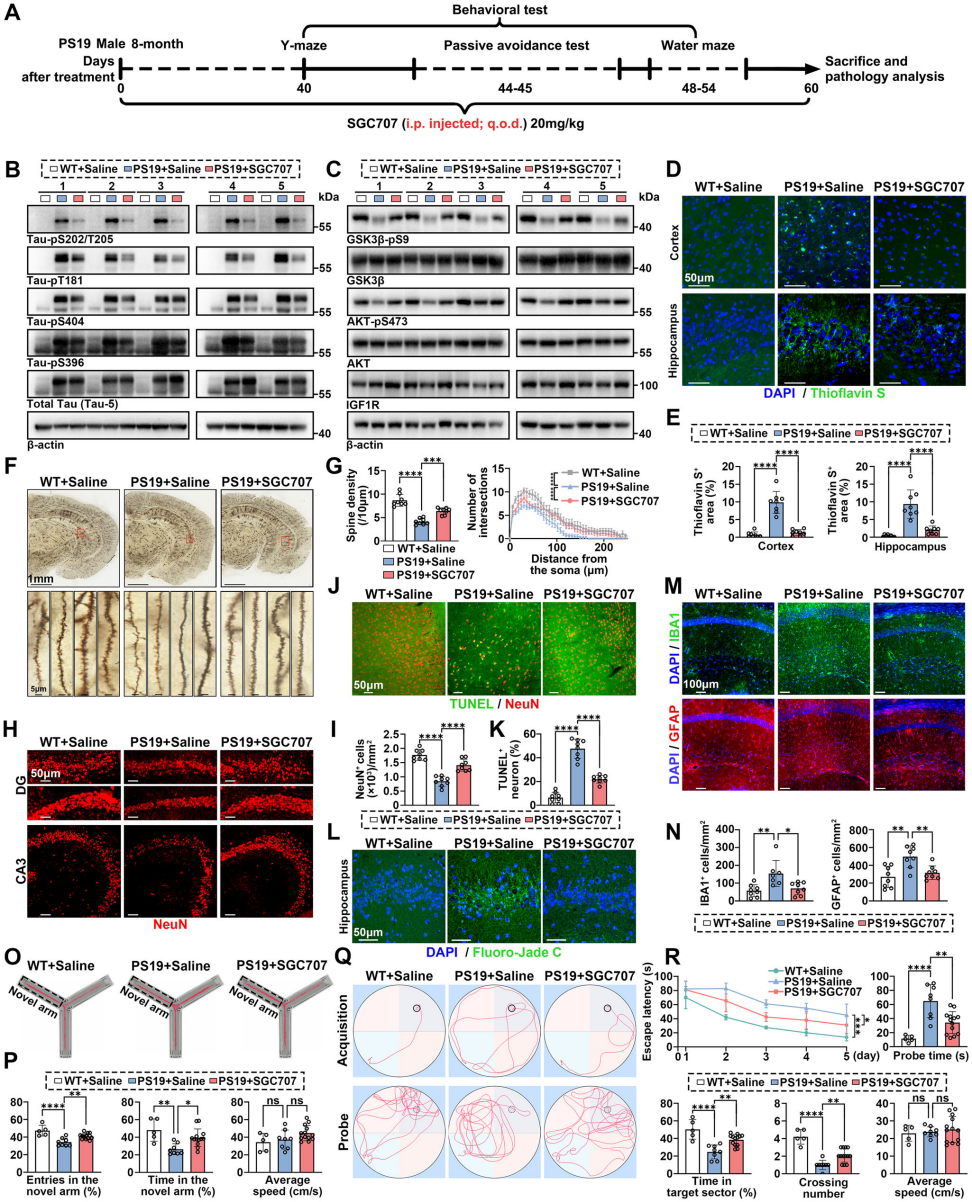
PRMT3 inhibition attenuates tau pathology and cognitive impairment in PS19 tauopathy mice. A) Schematic of the experimental timeline for treatment, behavioral testing, and pathological analysis in 8‐month‐old PS19 male mice. B) Western blot analysis of tau phosphorylation in the hippocampus from PS19 mice treated with SGC707 or saline. C) Western blot analysis of IGF1R, AKT, AKT‐pS473, GSK3β, and GSK3β‐pS9 in the hippocampus from PS19 mice treated with SGC707 or saline. D,E) Representative images and quantification of thioflavin S‐positive deposits (green) in the cortex and hippocampus of PS19 mice treated with SGC707 or saline. Scale bar, 50 µm. F) Representative images of Golgi staining brain in PS19 mice treated with SGC707 or saline. Scale bar, 1 mm, 5 µm. G) Quantification of spine density and Sholl analysis of dendritic complexity based on Golgi staining images. H,I) IF analysis of NeuN (red) in the hippocampus of PS19 mice treated with SGC707 or saline. Scale bar, 50 µm. J,K) Representative images and quantification of TUNEL (green) and NeuN (red) co‐staining in the cortex of PS19 mice treated with SGC707 or saline. Scale bar, 50 µm. L) Fluoro‐Jade C staining of degenerating neurons in the hippocampus of PS19 mice treated with SGC707 or saline. Scale bar, 50 µm. M,N) Representative images and quantification of IBA1 (green) and GFAP (red) co‐staining in the hippocampus of PS19 mice treated with SGC707 or saline. Scale bar, 100 µm. O,P) Representative path traces and quantification of the Y‐maze test performance of PS19 mice treated with SGC707 or saline. Q,R) Representative swim paths and quantification of the Morris water maze test performance of PS19 mice treated with SGC707 or saline. Quantified data are presented as mean ± SD. *n* = 8 fields per group (E, G, I, K, and N), *n* = 5 mice for WT+Saline, *n* = 8 mice for PS19+Saline, *n* = 13 mice for PS19+SGC707 (P, R). One‐way ANOVA followed by Tukey's multiple comparisons test, two‐way ANOVA followed by Tukey's multiple comparisons test (G). *p < 0.05, **p *<* 0.01, ***p < 0.001, ****p < 0.0001, ns = not significant.

To further investigate whether PRMT3 inhibition regulates tau phosphorylation in vivo, we administered i.p. injections of SGC707 at a dose of 20 mg kg^−1^ to eight‐month‐old male PS19 mice every other day for a period of two months (**Figure** **7**A). The mice were divided into three groups. Specifically, WT mice injected with saline were used as the normal baseline control group. PS19 mice injected with saline were designated as the vehicle treatment group, serving as the negative control for the disease model. PS19 mice injected with SGC707 constituted the treatment group. The mice were analyzed at ten months of age. As expected, injection of SGC707 significantly decreased H4R3me2a level in the hippocampus of PS19 mice, indicating the successful blood‐brain barrier penetration by SGC707 (Figure S7A,B, Supporting Information). The hyperphosphorylation of tau in the hippocampus of PS19 mice was attenuated following SGC707 administration, compared to saline‐treated controls (Figure 7B and Figure S7C, Supporting Information). Notably, miR‐448‐3p expression was significantly downregulated after SGC707 treatment (Figure S7D, Supporting Information). In addition, SGC707 administration led to an upregulation of IGF1R, AKT‐pS473, and GSK3β‐pS9 (Figure 7C and Figure S7E, Supporting Information). These findings suggest that SGC707 effectively reduces tau hyperphosphorylation in PS19 tauopathy mice brains by decreasing H4R3me2a levels at the MIR448 promoter, subsequently suppressing miR‐448‐3p expression, thereby upregulating its target gene IGF1R and activating the PI3K/AKT/GSK3β signaling pathway.

We assess the effect of SGC707 on tau pathology originates from abnormal tau hyperphosphorylation. Thioflavin S staining revealed a reduction of tau aggregates made up of hyperphosphorylated tau in the cortex and hippocampus of PS19 mice treated with SGC707 (Figure 7D,E). Golgi staining revealed that, compared to WT mice, PS19 mice exhibited a significant reduction in the complexity of neuronal dendritic arborization and the density of dendritic spines. Following SGC707 treatment, both dendritic complexity and spine density were significantly increased (Figure 7F,G). Dendritic spines were morphologically classified into four categories—mushroom, stubby, thin, and filopodium—each with distinct structural features and associated functions (Figure S7F, Supporting Information).^[^ ^29^ ^]^ Quantitative analysis revealed that, compared to WT mice, the density of stubby, thin, and mushroom spines was significantly reduced in PS19 mice. After SGC707 treatment, the density of mushroom spines was significantly restored, whereas no significant changes were observed in the other spine types (Figure S7G, Supporting Information). Due to their complex postsynaptic structures and high metabolic demands, they are particularly vulnerable to tau hyperphosphorylation‐induced synaptic dysfunction.^[^ ^30^ ^]^ By inhibiting PRMT3 activity, SGC707 reduces tau phosphorylation, alleviating tau‐associated synaptic toxicity. This preferential restoration of mushroom spines may result from their high susceptibility to tau‐induced dysfunction and suggests that SGC707 enhances synaptic stability and network integrity by targeting tau‐related toxicity.

Next, we evaluated neuronal loss in PS19 mice. There were pronounced reductions in neuronal densities in the hippocampus of saline‐treated PS19 mice, especially DG and CA3 region, yet no significant neuronal loss in SGC707‐treated PS19 mice (Figure 7H,I). Moreover, SGC707 treatment dramatically attenuated neuronal apoptosis and degeneration in the cortex and hippocampus of PS19 mice when compared with saline treatment by terminal deoxynucleotidyl transferase dUTP nick‐end labeling (TUNEL) assays and Fluoro‐Jade C staning, respectively (Figure 7J–L and Figure S7H,I). Beyond neurodegeneration, tau pathology is strongly associated with neuroinflammation, characterized by gliosis and elevated levels of pro‐inflammatory cytokines.^[^ ^31^ ^]^ Consistent with Zhang et al.,^[^ ^32^ ^]^ we observed hyperproliferation of glial fibrillary acidic protein (GFAP)‐positive astrocytes and ionized calcium binding adaptor molecule 1 (IBA1)‐positive microglia in the hippocampus of saline‐treated PS19 mice, whereas SGC707‐treated PS19 mice exhibited marked attenuation of both astrogliosis and microgliosis (Figure 7M,N). Analysis of hippocampal pro‐inflammatory cytokines IL‐1β and TNF‐α revealed significantly elevated expression levels in PS19 mice compared to WT controls. Notably, treatment with SGC707 markedly reduced the expression of both IL‐1β and TNF‐α in the hippocampus (Figure S7J, Supporting Information). Furthermore, no toxic effects were observed across all treatment groups, as indicated by body weight analysis and hematoxylin and eosin (HE) staining (Figure S7K,L).

To investigate the effect of SGC707 on cognitive function, we performed multiple behavioral assays. Mice injected with SGC707 entered the novel arm more frequently and spent more time in the novel arm during the Y‐maze test (Figure 7O,P). Consistently, in the water maze test, mice injected with SGC707 exhibited improved memory, demonstrated by a shorter latency to find the platform during the training phase, more crossings over the platform area, and more time spent in the target quadrant during the probe test compared to PS19 mice injected with saline (Figure 7Q,R). Additionally, SGC707 treatment effectively increased memory retention in the passive avoidance test (Figure S7M, Supporting Information). These results indicate that SGC707 reverses tau pathology and cognitive impairment in PS19 tauopathy mice.

## 3 Discussion

The tauopathies represent a clinically and pathologically heterogeneous group of neurodegenerative diseases characterized by the aberrant aggregation of hyperphosphorylated tau protein. This group includes primary tauopathies, such as progressive supranuclear palsy (PSP), corticobasal degeneration (CBD), Pick disease, frontotemporal dementia, and PART, as well as secondary tauopathies, including AD and chronic traumatic encephalopathy.^[^ ^33^ ^]^ Despite their diversity, these diseases are likely interconnected through shared underlying mechanisms that influence their manifestation.^[^ ^2a^ ^]^ Given tau pathology as a key common mechanism, tau has emerged as an attractive target for therapeutic development. Tau‐targeting antibody therapies have been the focus of most clinical trials to date.^[^ ^5^ ^]^ However, because tau is an endogenous protein, there exists a potential risk for adverse and possibly irreversible autoimmune responses. Identifying the upstream drivers of tau hyperphosphorylation and targeting them with small‐molecule inhibitors represents a promising clinical approach. Current efforts have largely focused on inhibiting GSK3β, which plays a major role in tau hyperphosphorylation.^[^ ^34^ ^]^ However, GSK3β is involved in various physiological pathways, including glucose metabolism, cell proliferation, and apoptosis, and its inhibition may lead to widespread off‐target effects.^[^ ^35^ ^]^ Therefore, targeting upstream regulatory mechanisms, such as specific molecular switches or epigenetic regulators involved in tau hyperphosphorylation, may provide a more natural means of restoring tau homeostasis with potentially fewer side effects compared to direct inhibition of downstream kinases.

Given the complexity of the human brain, studying tau pathology based on human brain tissue provides essential insights. However, the rarity of primary tauopathies, such as CBD, PSP, and Pick disease, presents substantial challenges in obtaining sufficient brain samples for research. Although AD is the most extensively studied tauopathy, its classification as a secondary tauopathy complicates efforts to clarify the independent mechanisms of tau hyperphosphorylation due to significant interactions with coexisting pathologies, particularly Aβ.^[^ ^13^ ^]^ These interdependent pathologies make AD less suitable for examining tau hyperphosphorylation as an independent process or for identifying common mechanisms shared across tauopathies. In contrast, PART presents a unique advantage. With a prevalence approaching 20% in individuals over 80,^[^ ^8a^ ^]^ PART brain samples are relatively accessible and provide an ideal model for studying tau hyperphosphorylation independently of confounding co‐pathologies.^[^ ^7^ ^]^ Although PART is associated with only mild cognitive decline, its lack of Aβ pathology suggests that PART could serve as a valuable model for identifying therapeutic targets that may have broader applicability across tauopathies.^[^ ^2a^ ^]^

Disease duration is a key variable in studying molecular mechanisms based on human samples. Both PART and AD are slow‐progressing diseases, with pathological and molecular changes likely occurring early and persisting for years. Given the challenges in accurately determining the specific disease duration for donors, we used Braak staging and ADNC scores as proxies to assess pathological severity. This approach helps reduce the confounding effects potentially arising from disease duration mismatches. Our study focuses on the mechanism underlying the regulation of tau phosphorylation level. By selecting samples free from other neurological conditions, with comparable neuropathological changes, and rigorously matching variables such as age and sex in the experimental design, we effectively minimized the impact of confounding factors.

In the present study, we focused on the EC region of PART patients, as it is the first brain region where tau hyperphosphorylation appears.^[^ ^36^ ^]^ More importantly, studies have reported that tau phosphorylation levels in the EC region are higher in PART patients than in AD patients—a surprising finding given that Aβ pathology is widely known to promote tau hyperphosphorylation, suggesting that tau phosphorylation should theoretically be more severe in AD, as is observed in the hippocampus.^[^ ^13^, ^14^ ^]^ This suggests the presence of unique drivers of tau hyperphosphorylation within the EC region in PART. Through transcriptomic analysis of human brain samples from both PART and AD patients, combined with in vivo and in vitro validation, we identified PRMT3 as a key driver of tau hyperphosphorylation in the EC region of PART patients. PRMT3 is a type I arginine methyltransferase that catalyzes the methylation of arginine residues, first generating a monomethyl arginine intermediate, which is subsequently converted to asymmetric dimethylarginine.^[^ ^37^ ^]^ PRMT3 has been extensively studied in oncology research, where it is recognized as a key regulator of metabolic reprogramming and gene expression in cancer.^[^ ^38^ ^]^ However, its role in tauopathies remains largely understudied.

H4R3me2a is one of the key modifications catalyzed by PRMT3, which is generally considered a mark associated with transcriptional activation.^[^ ^17^, ^21^, ^39^ ^]^ In this study, we observed an increase in nuclear localization of PRMT3 in the EC region of PART patients, accompanied by elevated levels of H4R3me2a catalyzed by PRMT3. Initially, our study aimed to explore whether H4R3me2a directly regulates the expression of an upstream gene involved in tau hyperphosphorylation. However, following SGC707 treatment, we observed a substantial upregulation of numerous genes, suggesting that the direct regulation of protein‐coding genes by H4R3me2a is limited. The widespread upregulation of mRNAs following PRMT3 inhibition reflects the complex regulatory network modulated by PRMT3 activity. While H4R3me2a is primarily associated with transcriptional activation, our ChIP‐seq data revealed that only a small fraction of H4R3me2a binding occurs at the promoter regions of protein‐coding genes. This limited promoter localization implies that the loss of H4R3me2a binding alone is insufficient to directly drive the extensive transcriptional changes observed. Instead, the upregulation of mRNAs is likely mediated by indirect mechanisms. Our study highlights the multifaceted role of miR‐448 in PRMT3/H4R3me2a‐mediated gene regulation. By intersecting three target prediction databases (TargetScan, miRDB, and miRWalk), we identified a subset of miR‐448 target genes and demonstrated its specific regulatory effect on IGF1R, which influences tau phosphorylation. This targeted analysis was instrumental in uncovering the H4R3me2a/miR‐448/IGF1R axis as a key pathway in the context of tauopathies. However, a broader analysis using the union of predicted miR‐448 targets revealed that miR‐448 likely participates in PRMT3/H4R3me2a‐induced gene dysregulation on a larger scale. Among 1275 predicted targets, 404 were significantly upregulated following SGC707 treatment, suggesting a broad regulatory role for miR‐448 in this context (Table S4, Supporting Information). These findings indicate that miR‐448 exerts both specific and widespread regulatory effects, depending on the context and the subset of target genes analyzed. Additionally, PRMT3's role in methylating non‐histone substrates, such as METTL14,^[^ ^40^ ^]^ c‐Myc,^[^ ^41^ ^]^ and HIF‐1α,^[^ ^19a^ ^]^ suggests that its inhibition can disrupt broader regulatory networks, leading to extensive mRNA expression changes. Among the 79 down‐regulated genes in RNA‐seq, many are associated with processes related to the cell cycle and mitochondrial function. Notably, RPS2, a known substrate of PRMT3,^[^ ^42^ ^]^ plays a critical role in various biological functions, including cell cycle regulation.^[^ ^43^ ^]^ The impact of PRMT3 enzymatic activity on RPS2 and other substrates may indirectly contribute to the observed downregulation of these genes. Collectively, these findings underscore the multifaceted roles of PRMT3 enzymatic activity in gene regulation, highlighting its impact on both direct transcriptional processes and indirect regulatory pathways. The widespread mRNA upregulation observed upon PRMT3 inhibition reflects the interplay of these mechanisms, extending beyond the direct effects of H4R3me2a binding. To establish the specificity of the PRMT3/H4R3me2a/miR‐448/IGF1R axis in regulating tau phosphorylation, this study employed a series of rescue experiments, including the use of SGC707 combined with a miR‐448 mimic and IGF1R overexpression coupled with a miR‐448 mimic. These experiments demonstrated that PRMT3 primarily modulates H4R3me2a, rather than other substrates, and that miR‐448 primarily targets IGF1R over alternative genes, serving as a key regulatory mechanism for tau phosphorylation. However, it is important to note that the potential secondary or synergistic effects of other substrates or target genes cannot be fully excluded and warrant further investigation in future studies.

Transcriptional and epigenetic factors are key regulators of microRNA expression. For instance, PRMT5 represses the transcription of miR‐99 family by symmetrical dimethylation of histone H4R3 (H4R3me2s).^[^ ^44^ ^]^ PRMT7 has been found to epigenetically repress the expression of miR‐24‐2 by upregulating H4R3me2s levels in the promoter region.^[^ ^22b^ ^]^ PRMT3 regulates osteogenesis in human mesenchymal stem cells by modulating H4R3me2a levels in the promoter region of MIR3648.^[^ ^23^ ^]^ Here, we demonstrate that in neurons within the EC region of PART brains, PRMT3 modulates H4R3me2a levels in the promoter region of MIR448, further activating the transcription of miR‐448. miR‐448 has been widely reported to play a significant role in tumorigenesis and cardiomyopathy.^[^ ^45^ ^]^ The present research revealed that miR‐448 facilitates tau hyperphosphorylation in PART by directly targeting the IGF1R. By binding to complementary sequences within the 3′ untranslated region of *IGF1R* mRNA, miR‐448 facilitates the formation of a silencing complex, leading to mRNA degradation and translational repression. This mechanism has been reported in both mouse and human studies.^[^ ^24^ ^]^ IGF1R is vital in the central nervous system, regulating neuronal growth, synaptic plasticity, and neuroprotection to support neuronal health and guard against neurodegeneration.^[^ ^46^ ^]^ Consistent with its neuroprotective role, research has reported that IGF1R deficiency leads to elevated levels of tau phosphorylation.^[^ ^25a^ ^]^ IGF1R exerts its effects by activating downstream pathways, including the PI3K/AKT pathway.^[^ ^47^ ^]^ As a well‐known downstream target of the PI3K/AKT branch, this signaling cascade inactivates GSK3β via phosphorylation at Ser9, preventing it from phosphorylating the Tau protein.^[^ ^48^ ^]^ We also found that miR‐448 suppresses IGF1R expression, consequently inhibiting the downstream PI3K/AKT/GSK3β pathway. This inhibition reduces phosphorylation of GSK3β at Ser9, leading to increased GSK3β activity and thereby promoting tau phosphorylation. This study offers a new perspective on therapies targeting tau hyperphosphorylation, including PRMT3 inhibition or miR‐448 inhibitors. Here, we used the PRMT3 inhibitor SGC707, which effectively suppressed tau phosphorylation and restored neuronal function in both cellular and mouse models of tau pathology. Notably, the PRMT3/H4R3me2a/miR‐448 axis identified in this study provides a novel mechanistic link between epigenetic regulation and tau hyperphosphorylation in PART, and its relevance may extend to other tauopathies, including PSP and CBD. Although these disorders exhibit distinct regional and cellular patterns of tau deposition—PSP predominantly affects the brainstem and basal ganglia, whereas CBD targets cortical neurons and glia—the upstream regulatory mechanisms driving tau pathology may converge on shared pathways involving PRMT3‐mediated histone arginine methylation and miR‐448‐dependent post‐transcriptional regulation.^[^ ^49^ ^]^ Specifically, PRMT3 may influence chromatin accessibility and transcriptional programs that govern tau phosphorylation and aggregation by mediating H4R3me2a, while miR‐448 may exacerbate tau pathology in PSP and CBD with its cell‐type and region‐specific regulatory effects. These potential shared mechanisms highlight the need for future investigations using PSP and CBD models or patient‐derived tissues to determine whether this axis represents a conserved regulatory pathway across tauopathies or exhibits disease‐specific differences, ultimately underscoring its potential as a broad‐spectrum therapeutic target.

Post‐translational modifications (PTMs) are key regulators of tau behavior and function, encompassing phosphorylation, glycosylation, ubiquitination, acetylation, methylation, glycation, nitration, polyamination, and SUMOylation.^[^ ^33^ ^]^ Among these, phosphorylation is the most extensively studied PTM and the central focus of this study. Other modifications, such as glycosylation and ubiquitination, have been implicated in tau pathology, but their exact roles remain unclear.^[^ ^50^ ^]^ Acetylation, however, has emerged as a critical modulator of tau function and pathology.^[^ ^51^ ^]^ Notably, hyperacetylated tau species are elevated during early Braak stages and enriched in NFTs.^[^ ^52^ ^]^ A compelling question arises as to whether PRMT3 might also regulate tau acetylation. As discussed above, the downstream effects of PRMT3 inhibition are broad. In our RNA‐seq analysis of SGC707‐treated SH‐SY5Y cells, we examined changes in the expression levels of enzymes associated with tau acetylation. Interestingly, we observed a notable upregulation of SIRT1, the primary deacetylase of tau, following SGC707 treatment (Table S3, Supporting Information). This finding suggests that PRMT3 inhibition could potentially reduce tau acetylation through SIRT1 upregulation, thereby alleviating tau pathology. While this hypothesis is intriguing, it is important to note that changes in protein expression do not necessarily translate to changes in enzymatic activity. Future studies are warranted to investigate the functional implications of SIRT1 upregulation and its role in PRMT3‐mediated regulation of tau acetylation.

In PS19 mice, we observed marked gliosis and elevated levels of pro‐inflammatory cytokines, hallmark indicators of neuroinflammation,^[^ ^31^ ^]^ which was alleviated following treatment with SGC707. This finding aligns with the growing recognition that tau pathology not only has direct neurotoxic effects but also acts as a key driver of neuroinflammation, a process that exacerbates disease progression.^[^ ^53^ ^]^ Activated glial cells play a pivotal role in this inflammatory response by releasing pro‐inflammatory cytokines such as IL‐1β and TNF‐α.^[^ ^54^ ^]^ These cytokines not only amplify tau pathology via feedback mechanisms but also impair synaptic function and plasticity, contributing to the cognitive deficits observed in tauopathy models and patients.^[^ ^55^ ^]^ The anti‐neuroinflammatory effects of SGC707 observed in this study are likely attributable to its impact on reducing tau hyperphosphorylation. Liao et al. reported that SGC707 does not influence glial cell proliferation, a feature commonly associated with gliosis, suggesting that its effects on neuroinflammation may be mediated indirectly through the modulation of tau pathology.^[^ ^19a^ ^]^ This finding underscores the robust cascade of downstream effects triggered by alleviating tau hyperphosphorylation, highlighting the therapeutic potential of targeting PRMT3 in tauopathies.

However, there were limitations in our study. In this study, postmortem interval (PMI) was not prioritized as a criterion for the inclusion of human brain tissue samples. This decision was based on our previous study, which utilized the same EC transcriptomic dataset as the current study and demonstrated, through PCA, that PMI contributed minimally to transcriptomic variation.^[^ ^15a^ ^]^ Instead, the primary source of variation was attributed to disease state. These findings suggest that while PMI may act as a potential confounding factor, its overall impact on data variability is limited. Nevertheless, future studies with expanded sample sizes should take PMI into consideration to ensure robustness and generalizability. We selected age‐ and gender‐matched AD patients as controls to compare with PART patients. While tau phosphorylation levels in the EC region of PART patients were notably higher than in AD patients, the complex pathology of AD brain tissue complicates the identification of factors specifically driving tau hyperphosphorylation. An ideal control group would consist of individuals matched in age and gender, without any AD neuropathological changes (Thal phase “A” = 0, Braak NFT stage “B” = 0, and CERAD neuritic plaque “C” = 0) or other brain pathology; however, such samples are exceedingly rare. With a sufficiently large pool of these samples, a single‐cell transcriptomic comparison with PART patients could reveal key regulatory elements that more accurately illuminate mechanisms underlying tau pathology. Moreover, we observed an increase in PRMT3 nuclear localization within the EC neurons of PART patients, yet PRMT3 lacks a nuclear localization sequence, indicating a need for further investigation into its nuclear entry mechanism. We employed PS19 mice as a model to simulate PART, given that tau pathology in these mice is concentrated in the hippocampus and EC region, which aligns with the early distribution of tau pathology in PART.^[^ ^7^, ^18^ ^]^ However, the aggressive and rapid disease progression observed in PS19 mice starkly contrasts with the slow, age‐related progression characteristic of PART. To provide a more comprehensive foundation for clinical translation, further validation using a diverse range of tauopathy mouse models and naturally aging models is warranted. Notably, SGC707 has not yet been investigated in clinical studies for any disease. Following cross‐validation across these models, long‐term follow‐up studies should be considered to assess the durability and translational potential of therapeutic effects. Furthermore, recent advances in PRMT3‐targeted protein degradation tools have demonstrated enhanced specificity compared to traditional methods, presenting a promising direction for the development of more precise therapeutic strategies.^[^ ^56^ ^]^ Applying these new tools in PS19, AD models, and related tauopathy animal models holds promise for advancing therapeutic strategies targeting tau pathology. In addition, overexpressing PRMT3 in tauopathy models could potentially exacerbate tau pathology, leading to a faster and more severe progression of disease. This approach may help establish a model that mimics an accelerated and intensified tauopathy phenotype, which could be valuable for investigating therapeutic interventions targeting tau hyperphosphorylation.

## 4 Conclusion

**Figure 8:**
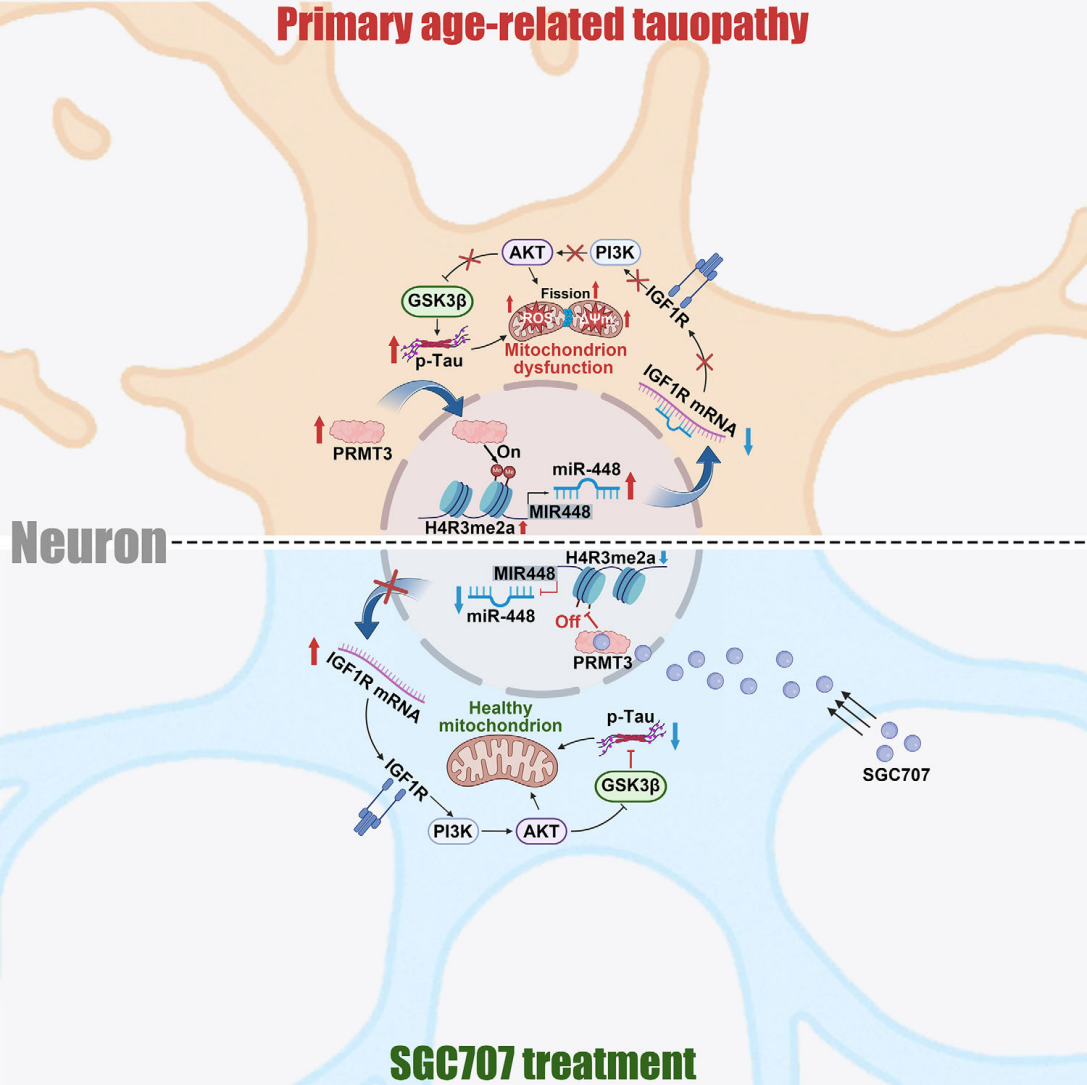
The mechanistic scheme of PRMT3 facilitates tau hyperphosphorylation in PART. PRMT3 directly mediates H4R3me2a at the promoter of MIR448, leading to increased miR‐448 expression. Elevated miR‐448 suppresses the expression of its target gene IGF1R, resulting in the dysregulation of the PI3K/AKT/GSK3β signaling pathway, ultimately driving GSK3β activation and tau hyperphosphorylation, and PRMT3 inhibition significantly reduced tau pathology and restored neuronal function both in vitro and in vivo.

In summary, we identified PRMT3 as a critical driver of tau hyperphosphorylation in PART. This study elucidates a new epigenetic mechanism in which PRMT3 directly mediates the asymmetric dimethylation of H4R3 (H4R3me2a) at the promoter of MIR448, leading to increased miR‐448 expression. Elevated miR‐448 suppresses the expression of its target gene IGF1R, resulting in the dysregulation of the PI3K/AKT/GSK3β signaling pathway, ultimately driving GSK3β activation and tau hyperphosphorylation. Notably, inhibition of PRMT3 with SGC707 significantly reduced tau pathology and restored neuronal function both in vitro and in vivo (**Figure** **8**). These findings highlight PRMT3 as a promising therapeutic target for PART and potentially other tauopathies.

## 5 Experimental Section

### Ethical Statement

All animal procedures conducted in this study were reviewed and approved by the Institutional Animal Care and Use Committee in the Chinese Academy of Medical Sciences, Institute of Basic Medical Sciences (Approval Number: ACUC‐XMSB‐2024‐016). The present study was approved by the Institutional Review Board of the Institute of Basic Medical Sciences, Chinese Academy of Medical Sciences (Approval Number: 009‐2014, 031‐2017, and 2022125).

### Reagents

The reagents used in this study, including but not limited to antibodies, primers, and siRNA, are all listed in Table S5 (Supporting Information).

### Quantification and Statistical Analysis

Images were analyzed and quantified using ImageJ and Imaris. All data were presented as the mean ± SD and analyzed using GraphPad Prism 8.0 software. Statistical analysis was performed using either Two‐tailed *t* test (two‐group comparison), one‐way ANOVA followed by Dunnett's or Tukey's multiple comparisons test (more than two groups), or two‐way ANOVA followed by Tukey's multiple comparisons test (two categorical independent variables). All statistical tests were two‐sided. Differences with *P* values less than 0.05 were considered significant. Additional details of the materials and methods can be found in the Supporting Information.

## Conflict of Interest

The authors declare no conflict of interest.

## Author Contributions

H.L. and X.L. contributed equally to this work. W.G. and C.M. conceived the project and provided guidance throughout the study. H.L. designed the study and wrote the manuscript. X.W. revised the manuscript. H.L., X.L., F.T., Y.C., J.L., and X.W. performed experiments. H.L. and X.L. analyzed the data. W.Q. and X.W. were responsible for human brain dissection and staining. All authors read and approved the final manuscript.

## Supporting information



Supporting Information

Supporting Information

Supporting Information

Supporting Information

Supporting Information

Supporting Information

## Data Availability

The data that support the findings of this study are available from the corresponding author upon reasonable request.
