# Supplementary material for: PRMT3‐Mediated H4R3me2a Promotes Primary Age‐Related Tauopathy by Driving Tau Hyperphosphorylation in Neuron
Source: Adv Sci (Weinh). 2025 May 8;12(28):2506044. doi: 10.1002/advs.202506044 (PMC12302536; doi:10.1002/advs.202506044)
Supplement: Supplementary file 1 — Supporting Information [file ADVS-12-2506044-s003.pdf]

ADVANCED SCIENCE Open Access

Supporting Information

for *Adv. Sci.*, DOI 10.1002/advs.202506044

PRMT3-Mediated H4R3me2a Promotes Primary Age-Related Tauopathy by Driving Tau Hyperphosphorylation in Neuron

Haotian Liu, Xinnan Liu, Fengyuan Tian, Yashuang Chen, Jingying Li, Xue Wang, Wenying Qiu, Xia Wang, Chao Ma* and Wei Ge**

Supporting Information

PRMT3-mediated H4R3me2a promotes primary age-related tauopathy by driving tau hyperphosphorylation in neuron

Haotian Liu, Xinnan Liu, Fengyuan Tian, Yashuang Chen, Jingying Li, Xue Wang, Wenying Qiu, Xia Wang, Chao Ma*, Wei Ge**

Dr. H. Liu, Dr F Tian, Dr Y Chen, Dr. J. Li, Prof. X. Wang, Prof. W. Ge

Department of Immunology, State Key Laboratory of Complex, Severe, and Rare Diseases, Institute of Basic Medical Sciences Chinese Academy of Medical Sciences, School of Basic Medicine Peking Union Medical College, Beijing, 100005, China

E-mail: wangxia@ibms.pumc.edu.cn; gewei@ibms.cams.cn

Dr. H. Liu, Dr. X. Liu, Dr. X. Wang, Prof. W. Qiu, Prof. C. Ma

Department of Human Anatomy, Histology and Embryology, Neuroscience Center, National Human Brain Bank for Development and Function, Institute of Basic Medical Sciences Chinese Academy of Medical Sciences, School of Basic Medicine Peking Union Medical College, Beijing, 100005, China

E-mail: machao@ibms.cams.cn

Table of Contents

Supplementary Figures 3

Supplementary Methods 13

The orginal uncropped images and normalized gray values of western blot

....21

Supplementary Figures

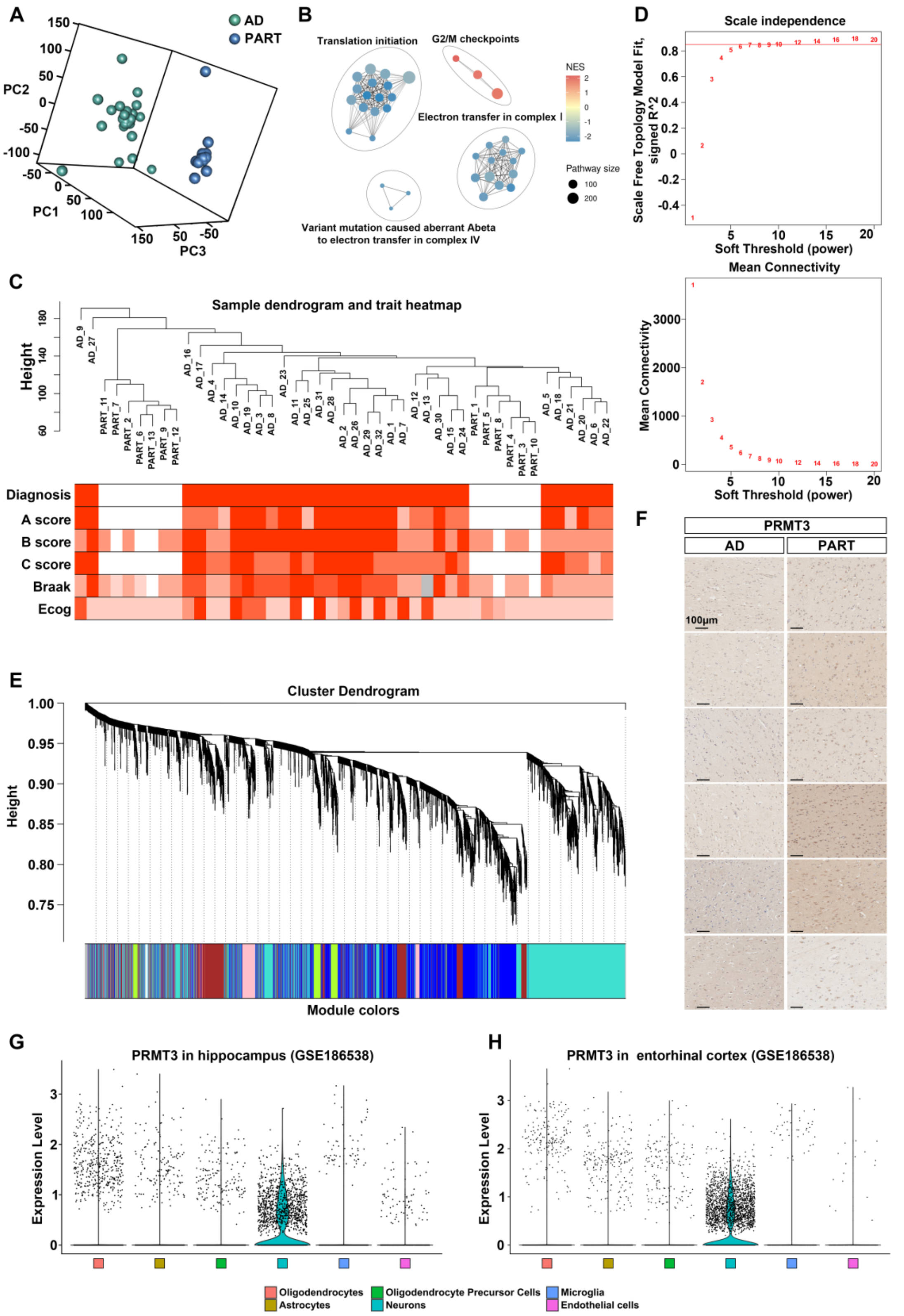

Figure S1. Transcriptomic profiling of the entorhinal cortex in patients with PART and AD, related to Figure 1. A) Principal component analysis plot of EC region RNA-Seq data. n = 32 for AD, n = 13

for PART. Each dot represents a sample. B) Network analysis of the Gene Set Enrichment Analysis results. C) Soft-thresholding power analysis was used to obtain the scale-free fit index of network topology. D) Hierarchical clustering dendrogram of samples and trait heat map. E) Hierarchical cluster analysis was conducted to detect co-expression clusters with corresponding color assignments. F) IHC analysis of PRMT3 in the EC region from PART and AD patients, related to Figure 1M. Scale bar, 100 μm. G-H) Single-nucleus RNA sequencing (snRNA-seq) data from dataset GSE186538 illustrate the expression of PRMT3 across major brain cell types in the hippocampus (G) and entorhinal cortex (H).

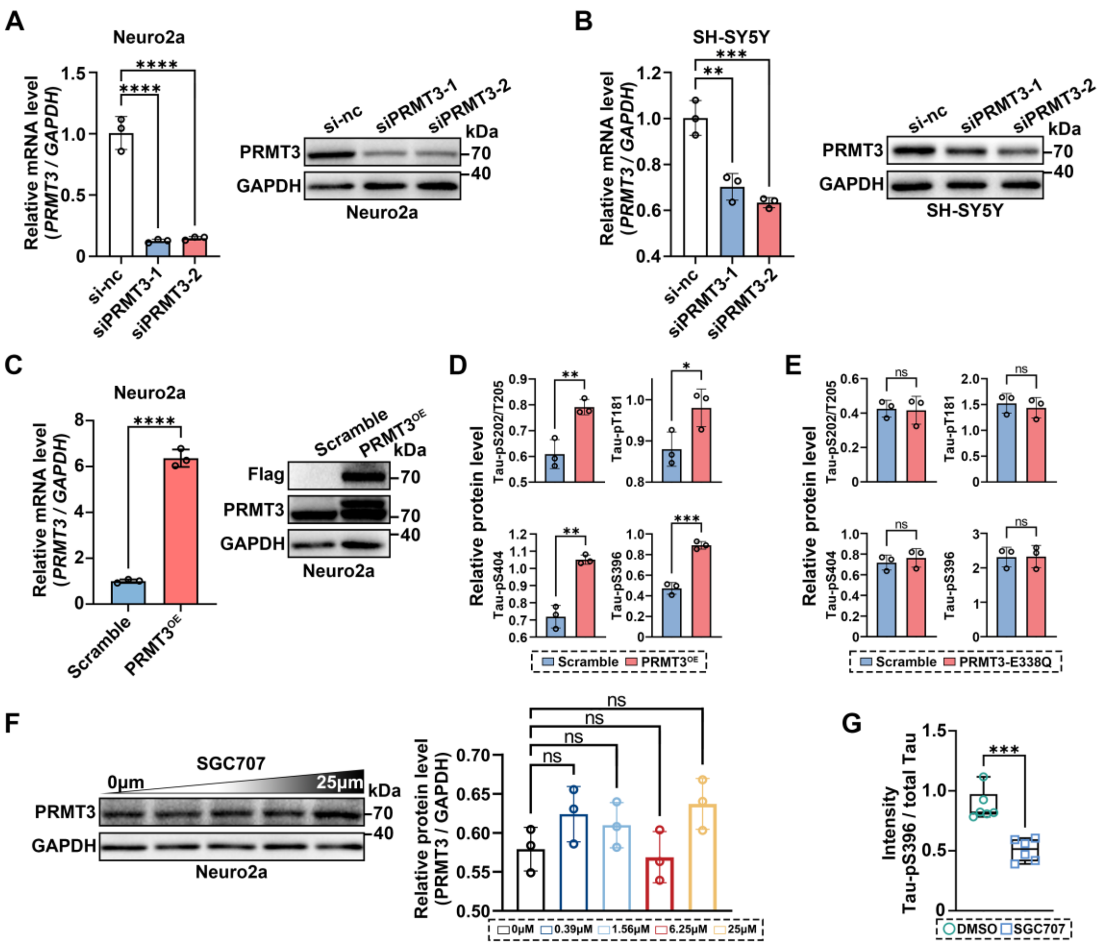

Figure S2. Construction of the cell lines with altered PRMT3 expression, related to Figure 2. A) qPCR and western blot analysis of PRMT3 (normalized to GAPDH) in negative control and siPRMT3 transfected Neuro2a cells. B) qPCR and western blot analysis of PRMT3 (normalized to GAPDH) in negative control and siPRMT3 transfected SH-SY5Y cells. C) qPCR and western blot analysis of PRMT3 (normalized to GAPDH) in Neuro2a cells transfected with a lentiviral vector for PRMT3 overexpression and a scramble control. D-E) Quantification of the relative level of tau phosphorylation (normalized to total tau) in Neuro2a cells overexpressing PRMT3 and PRMT3-E338Q. F) Western blot and quantification of PRMT3 (normalized to GAPDH) in Neuro2a cells treated with a concentration gradient of SGC707. G) IF analysis of tau-pS396 in mouse primary neurons. Quantified data are presented as mean ± SD. n = 3 per group (A-F), n = 6 per group (G). Unpaired Student's *t* test (C, D, and E) or one-way ANOVA followed by Dunnett's multiple comparisons test (A, B, and F), *$P < 0.05$, **$P < 0.01$, ***$P < 0.001$, ****$P < 0.0001$, ns = not significant.

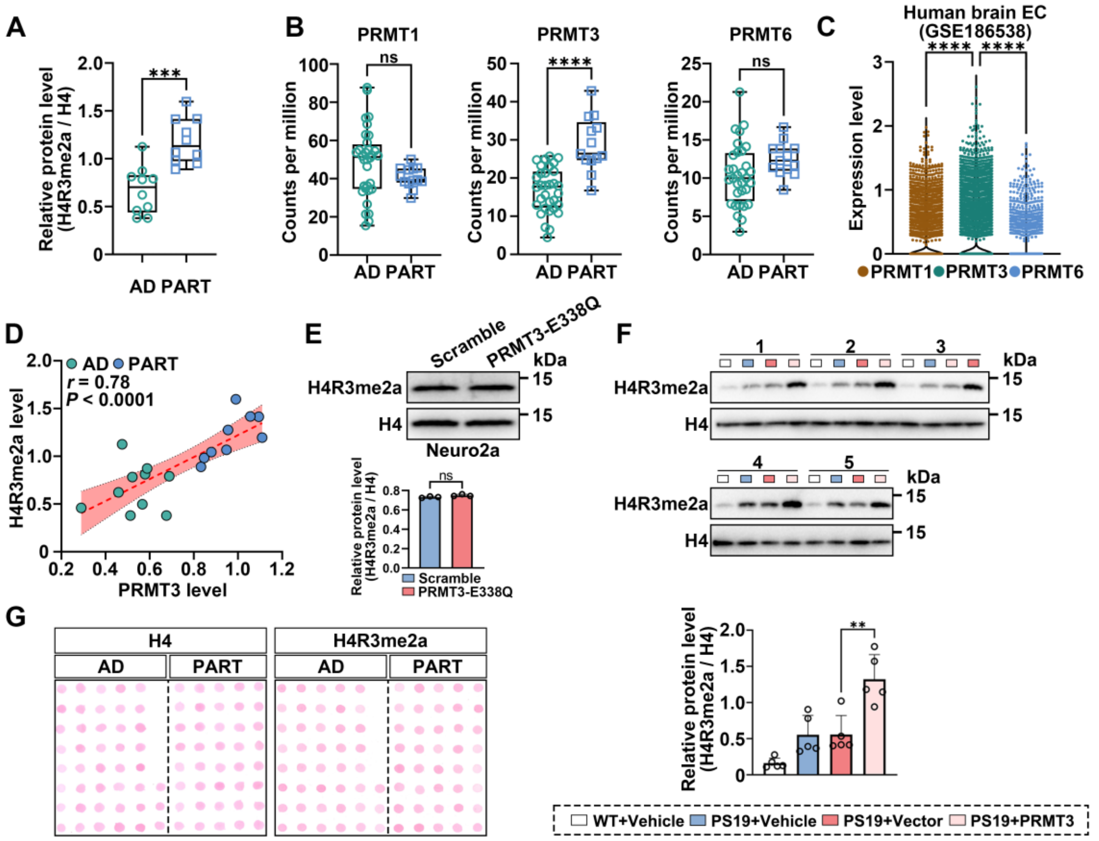

Figure S3. PRMT3 mediates H4R3me2a in the EC region of PS19 mice and PART patients, related to Figure 4. A) Quantification of relative H4R3me2a level (normalized to H4) in EC lysates from PART and AD patients. n = 10 for AD, n = 10 for PART. B) Transcript levels of PRMT1, PRMT3, and PRMT6 in EC lysates from PART and AD patients. n = 32 for AD, n = 13 for PART. C) Expression levels of PRMT1, PRMT3, and PRMT6 in the human brain EC region from the GEO dataset (GSE186538). D) Correlation analysis between PRMT3 and H4R3me2a levels in the EC region of PART and AD patients. n = 10 for AD, n = 10 for PART. E) Western blot and quantification of H4R3me2a (normalized to H4) in Neuro2a cells overexpressing PRMT3-E338Q. n = 3 per group. F) Western blot and quantification of H4R3me2a levels (normalized to H4) in the EC region of PS19 mice with AAV-induced neuron-specific PRMT3 overexpression. n = 5 per group. G) Ponceau S staining of dot blot membrane. n = 43 for AD, n = 40 for PART. Quantified data are presented as mean ± SD. Unpaired two-tailed Student's *t* test (A, B, and E) or one-way ANOVA followed by Dunnett's multiple comparisons test (C and F). $^{**}P < 0.01$, $^{***}P < 0.001$, $^{****}P < 0.0001$, ns = not significant.

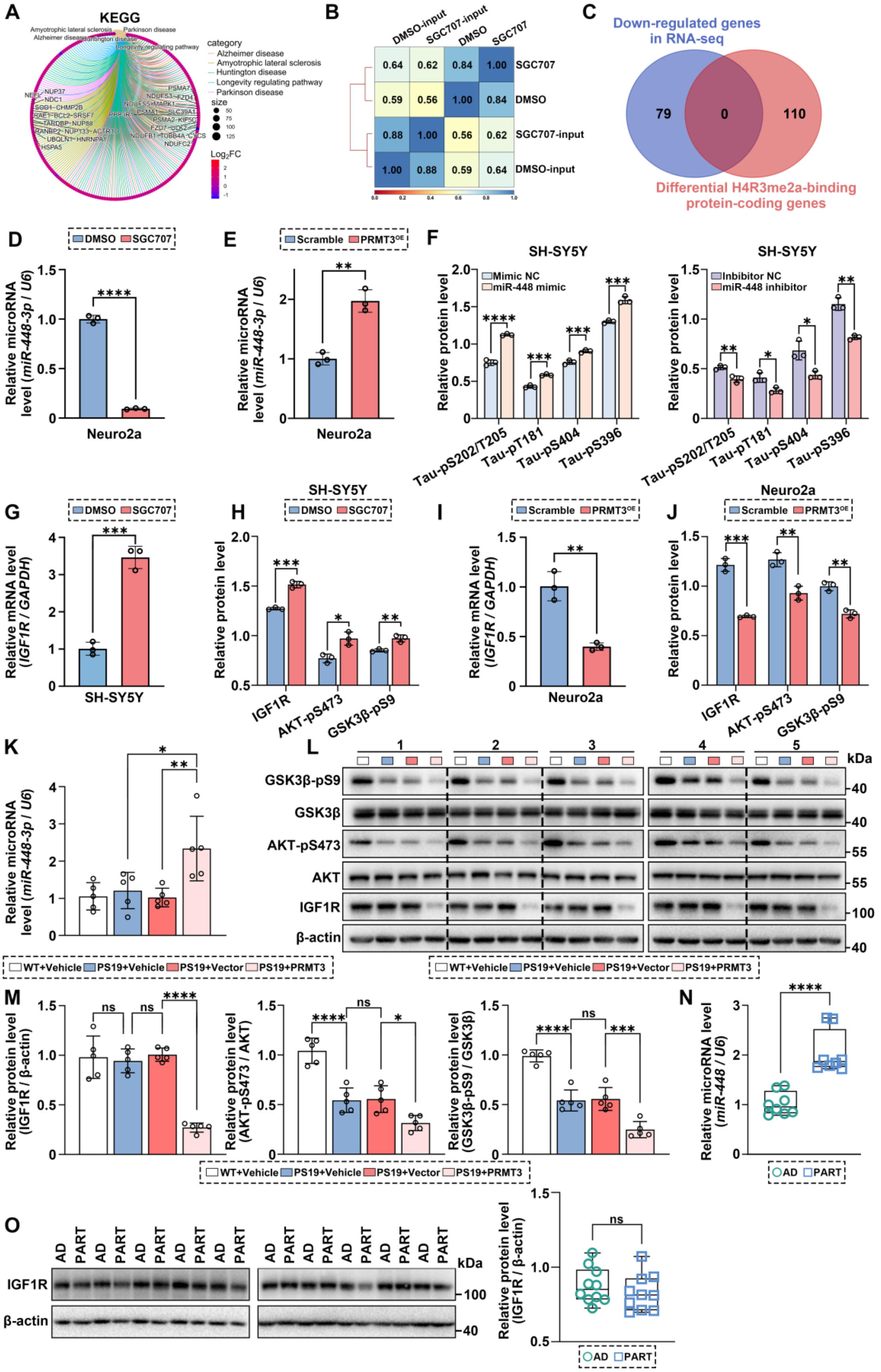

Figure S4. Verification of PRMT3-mediated regulation of the miR-448/IGF1R/AKT/GSK3β axis, related to Figure 5. A) KEGG pathway enrichment analysis of the DEGs in SGC707-treated SH-SY5Y

cells. B) Heatmap showing correlation matrix of ChIP-seq data from SGC707-treated SH-SYSY cells. C) Venn diagram illustrating the absence of overlap between genes down-regulated in RNA-seq and protein-coding genes with differential H4R3me2a binding identified from ChIP-seq. D) qPCR analysis of miR-448-3p (normalized to U6) in Neuro2a cells treated with SGC707. E) qPCR analysis of miR-448-3p (normalized to U6) in Neuro2a cells overexpressing PRMT3. F) Quantification of the relative level of tau phosphorylation (normalized to total tau) in SH-SY5Y cells transfected with miR-448 mimic or inhibitor. G) qPCR analysis of IGF1R (normalized to GAPDH) in SGC707-treated SH-SY5Y cells. H) Quantification of the relative level of IGF1R (normalized to GAPDH), AKT-pS473 (normalized to AKT), and GSK3β-pS9 (normalized to GSK3β) levels in SGC707-treated SH-SY5Y cells. I) qPCR analysis of IGF1R (normalized to GAPDH) in Neuro2a cells overexpressing PRMT3. J) Quantification of the relative level of IGF1R (normalized to GAPDH), AKT-pS473 (normalized to AKT), and GSK3β-pS9 (normalized to GSK3β) levels in Neuro2a cells overexpressing PRMT3. K) qPCR analysis of miR-448-3p (normalized to U6) in PS19 mice with AAV-induced neuron-specific PRMT3 overexpression. n = 5 per group. L-M) Western blot and quantification of IGF1R (normalized to β-actin), AKT-pS473 (normalized to AKT), and GSK3β-pS9 (normalized to GSK3β) levels in the EC region of PS19 mice with AAV-induced neuron-specific PRMT3 overexpression. n = 5 per group. N) qPCR analysis of miR-448 (normalized to U6) in the EC region of AD and PART patients (n = 8 per group). O) Western blot and quantification showing IGF1R protein levels in the EC region of AD and PART patients (n = 10 per group). Quantified data are presented as mean ± SD. n = 3 per group unless otherwise specified. Unpaired two-tailed Student's *t* test or one-way ANOVA followed by Dunnett's multiple comparisons test (K and M). *$P < 0.05$, **$P < 0.01$, ***$P < 0.001$, ****$P < 0.0001$, ns = not significant.

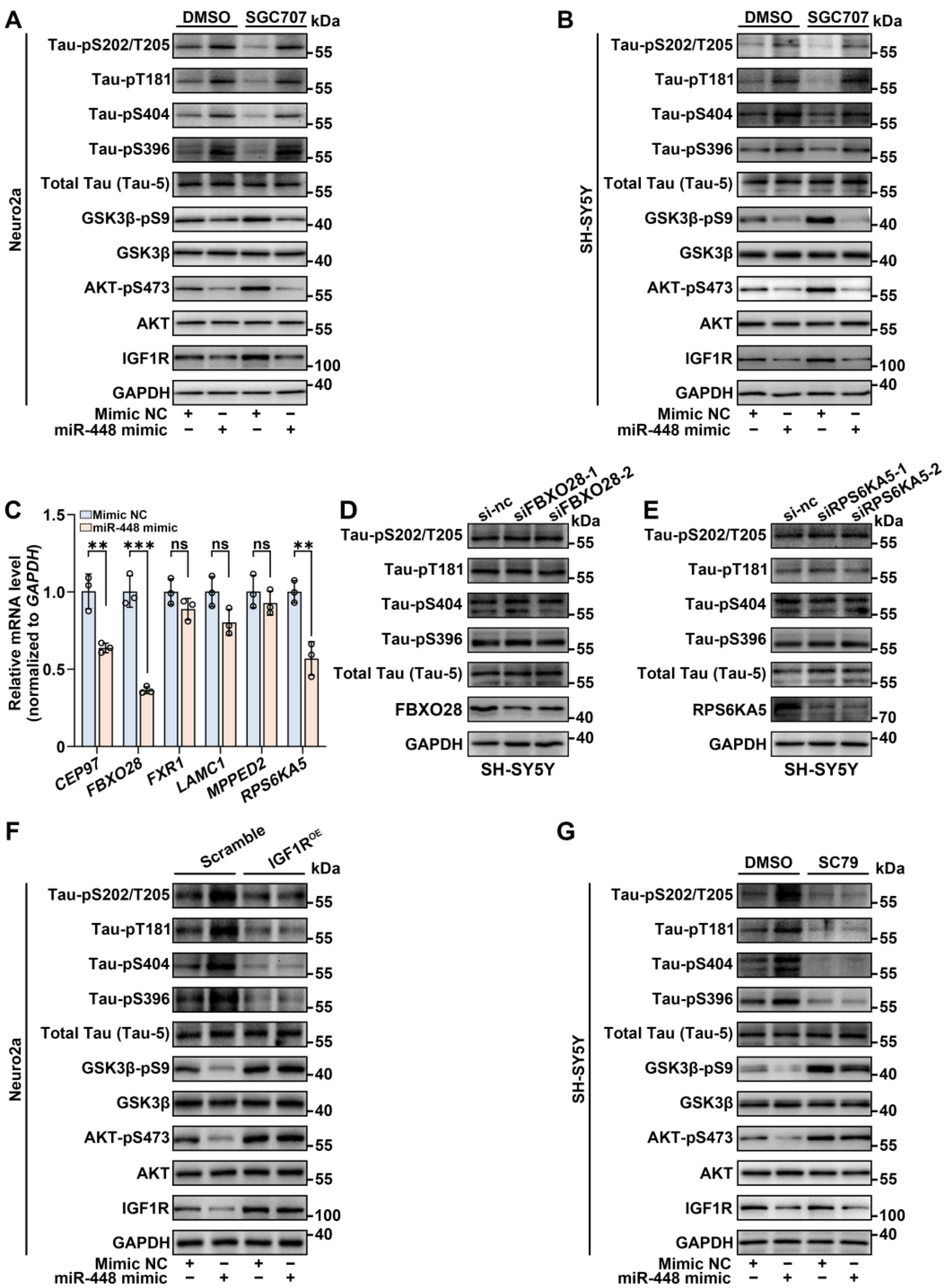

Figure S5. Verification the specificity of PRMT3-mediated regulation of the H4R3me2a/miR-448/IGF1R/AKT/GSK3β axis, related to Figure 5. A-B) Validation of the specificity of PRMT3/H4R3me2a/miR-448 axis in regulating tau phosphorylation using SGC707 combined with miR-448 mimic in Neuro2a and SH-SY5Y cells. C) qPCR quantification of the expression of six potential miR-448 target genes following miR-448 mimic treatment in SH-SY5Y cells. D-E) Tau phosphorylation status following FBXO28 or RPS6KA5 knockdown in SH-SY5Y cells. F) Validation of the specificity of miR-448/IGF1R axis in regulating tau phosphorylation using miR-448 mimic in IGF1R-overexpressing Neuro2a cells. G) Validation of the specificity of IGF1R/PI3K/AKT/GSK3β axis in regulating tau phosphorylation using SC79 combined with miR-448 mimic in SH-SY5Y cells. Quantified data are presented as mean ± SD. n = 3 per group. Unpaired two-tailed Student's *t* test. $**P < 0.01$, $***P < 0.001$, ns = not significant.

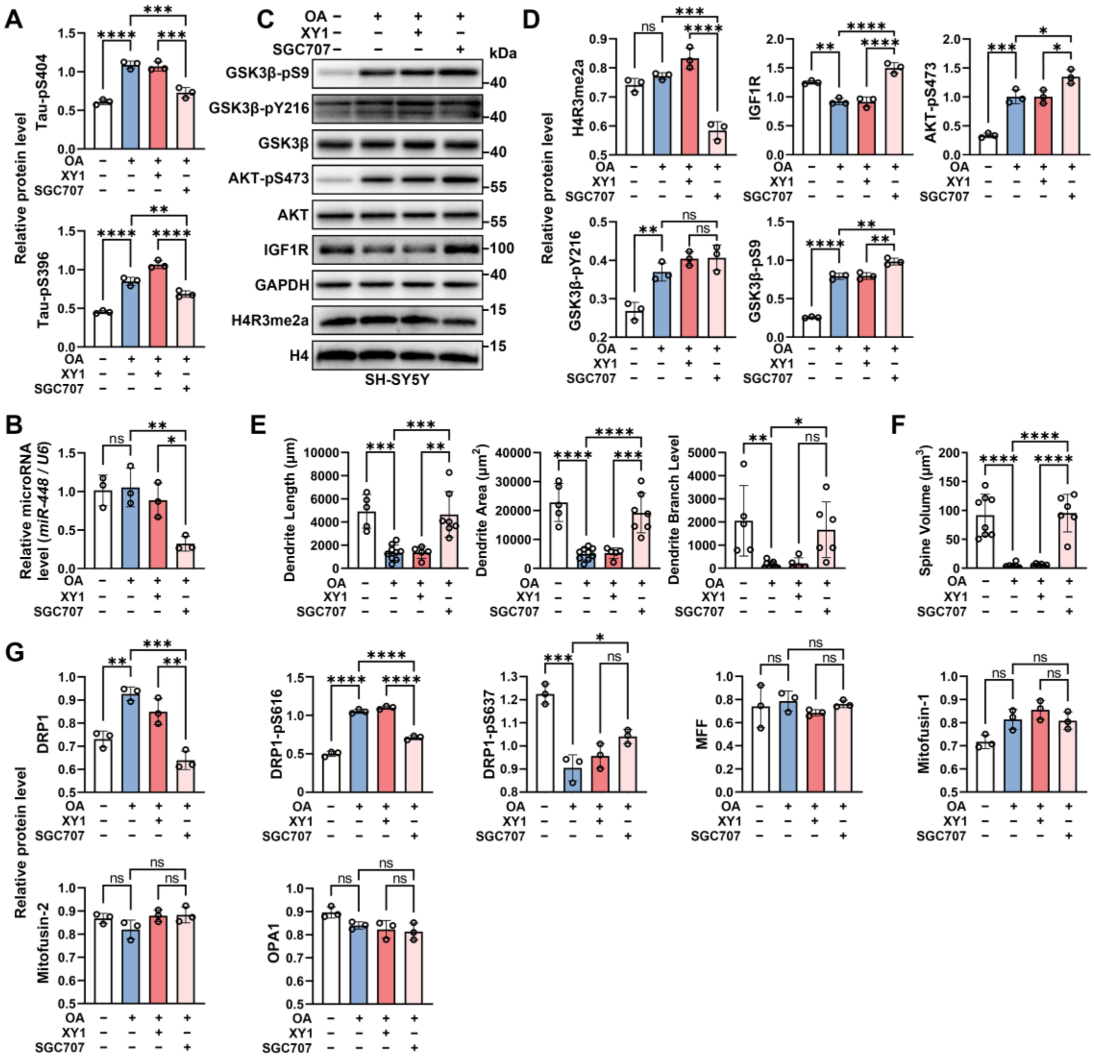

Figure S6. Effect of PRMT3 inhibition by SGC707 on tau hyperphosphorylation, neuronal function, and mitochondrial function in SH-SY5Y cells, related to Figure 6. A) Quantification of relative tau phosphorylation level (normalized to total tau) in tauopathy SH-SY5Y cells treated with SGC707 or XY1. B) qPCR analysis of miR-448 expression levels (normalized to U6) in tauopathy SH-SY5Y cells treated with SGC707 or XY1. C-D) Western blot and quantification of H4R3me2a (normalized to H4), IGF1R (normalized to GAPDH), AKT-pS473 (normalized to AKT), GSK3β-pY216 (normalized to GSK3β), and GSK3β-pS9 (normalized to GSK3β) levels in tauopathy SH-SY5Y cells treated with SGC707 or XY1. E-F) Dendritic morphology and spine analysis of tauopathy SH-SY5Y cells treated with SGC707 or XY1. n = 5-9 per group. G) Quantification of relative level of mitochondrial dynamics proteins (DRP1, MFF, MFN2, MFN1, OPA1, normalized to GAPDH) and DRP1 phosphorylation at pS616 and pS637 (normalized to DRP1) in tauopathy SH-SY5Y cells treated with SGC707 or XY1. n = 3 per group unless otherwise specified. Quantified data are presented as mean ± SD. One-way ANOVA followed by Tukey's multiple comparisons test. **P* < 0.05, ***P* < 0.01, ****P* < 0.001, *****P* < 0.0001, ns = not significant.

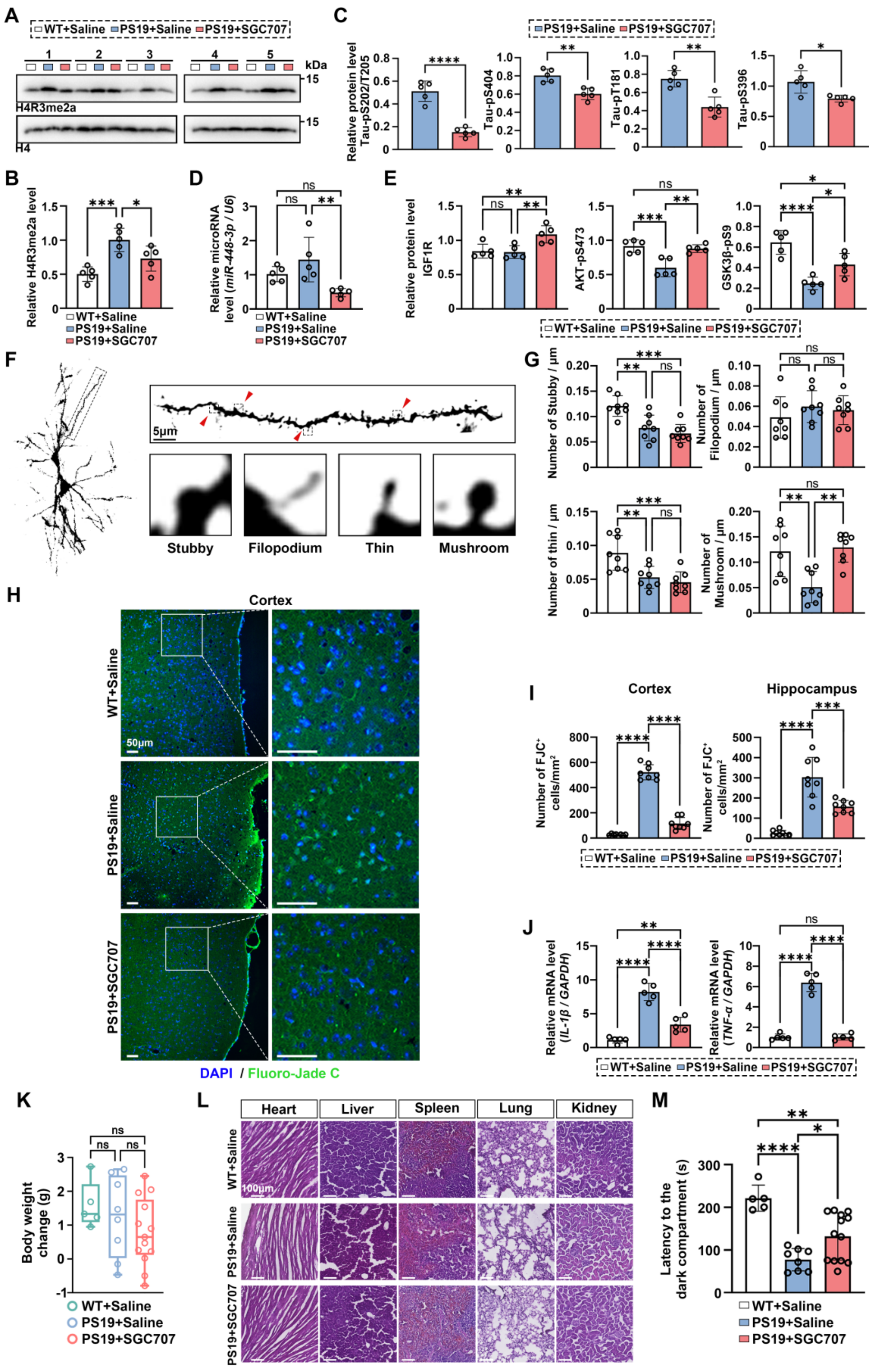

Figure S7. Effect of PRMT3 inhibition by SGC707 on tau pathology and cognitive function in PS19 tauopathy mice, related to Figure 7. A-B) Western blot and quantification of H4R3me2a (normalized

with H4) in the hippocampus from PS19 mice treated with SGC707 or saline. C-D) Quantification of the relative level of tau phosphorylation (normalized to total tau) and qPCR analysis of miR-448-3p (normalized to U6) in the hippocampus from PS19 mice treated with SGC707 or saline. E) Quantification of the relative level of IGF1R (normalized to β-actin), AKT-pS473 (normalized to AKT), and GSK3β-pS9 (normalized to GSK3β) levels in the hippocampus from PS19 mice treated with SGC707 or saline. F) Representative Golgi staining image of a hippocampus neuron in the PS19 mouse brain for spine classification. Scale bar, 5 μm. G) Quantification of the number of different types of spines before and after SGC707 treatment. H-I) Representative images and quantification of Fluoro-Jade C staining showing degenerating neurons in the cortex of PS19 mice treated with SGC707 or saline. Scale bar, 50 μm. J) Quantification of the relative level of IL-1β and TNF-α (normalized to GAPDH) in the hippocampus from PS19 mice treated with SGC707 or saline. K-L) Body weight changes and histopathological analysis of major organs (heart, liver, spleen, lung, kidney) of PS19 mice treated with SGC707 or saline. Scale bar, 100 μm. M) Latency to enter the dark compartment of PS19 mice treated with SGC707 or saline in passive avoidance test. Quantified data are presented as mean ± SD. n = 5 mice per group (B-E, and J), n = 8 fields per group (G), n = 5 mice for WT+Saline, n = 8 mice for PS19+Saline, n = 13 mice for PS19+SGC707 (K and M). Unpaired two-tailed Student's *t* test (C), One-way ANOVA followed by Tukey's multiple comparisons test (B, D, E, G, I, J, K, and M). $*P < 0.05$, $**P < 0.01$, $***P < 0.001$, $****P < 0.0001$, ns = not significant.

Supplementary Methods

Primary neuronal cultures

Primary neuronal cultures were prepared as described previously.[1] Briefly, primary neurons were extracted from the hippocampus and cortex of 1-day-old C57BL/6 mice and coverslips were coated with 0.01% poly-L-lysine (Sigma-Aldrich, P4707). Brain tissues were dissociated using 0.25% trypsin (Hyclone, SH30042.01) and 5 mg/mL DNase (Roche, 10104159001). Following digestion and centrifugation, cell supernatant was collected and filtered through a 70 μm cell strainer (Falcon, 352350). Neurons were then seeded on poly-L-lysine-coated glass coverslips in 24-well plates and cultured in Neurobasal Medium (Gibco, 21103049) supplemented with 2% B27 (Gibco, 12587010), 0.25% L-glutamine (Gibco, 25030149), and 0.5% penicillin-streptomycin (Gibco, 15140122). Neurons were cultured for 14 d before treatment.

Cell lines and culture

The mouse neuroblastoma cell line Neuro2a and the human neuroblastoma cell line SH-SY5Y were provided by the Cell Resource Center, Peking Union Medical College (PCRC). All cells were examined to determine the absence of mycoplasma contamination. Neuro2a cells were cultured in a MEM/EBSS medium (Hyclone, SH30024.01), supplemented with 10% fetal bovine serum (FBS; Gibco, 10091148). The SH-SY5Y cells were maintained in DMEM/F12 Medium (HyClone, SH30023.01) with 10% FBS.

Drug treatment for cells

Neuro2a cells were treated with a gradient concentration of SGC707 (MedChemExpress, HY-19715) ranging from 0 to 25 μM for 6 h. SH-5Y5Y cells were treated with SGC707 (10 μM in DMSO) for 6 h. Primary neurons were treated with SGC707 (10 μM in DMSO) for 6 h. SH-5Y5Y cells were treated with SC79 (MedChemExpress, HY-18749, 10 μM in DMSO) for 1 h.

To establish the *in vitro* model of tauopathy which was hyperphosphorylation of tau, SH-SY5Y cells were induced by incubation with okadaic acid (OA; J&K Scientific, 288967) at a final concentration of 20 nM for 12 h and primary neurons were induced by incubation with OA at a final concentration of 10 nM for 4 h, according to the different sensitivity to OA. For cell treatment, OA-damaged SH-SY5Y cells or neurons were replaced with medium containing SGC707 or XY1 (MedChemExpress, HY-19714) at 10 μM for 6 h.

Transfection

Specific siRNA, as well as miR-448 mimic and inhibitor (RiboBio, Guangzhou, China), were transfected into Neuro2a and SH-SY5Y cells using Lipofectamine RNAiMAX (Invitrogen, 13778075) in accordance with the manufacturer's protocol. The target sequences of the siRNA are listed in Table S5.

Flag-tagged IGF1R, Flag-tagged PRMT3 and its E338Q mutant were cloned into the 3×flag-LvCP06 vector (Yile Biotechnology, Shanghai, China) for use as lentiviral expression vectors. Lentivirus production was carried out by co-transfecting HEK293T cells with the lentiviral expression vectors and a lentiviral packaging plasmid mix (Gag-Pol, Rev, VSVG) following standard protocols. Transfections were performed using Lipofectamine 3000 (Invitrogen, L3000015).

Animals

Tau P301S (PS19; 008169; n = 36) mice and littermate wild-type (WT) mice (n = 10) obtained from Jackson Labs were used in this study. Because male PS19 mice show earlier and more pronounced alterations in brain function and tau phosphorylation compared to female mice,[2] only male mice were evaluated in this study. All animals were housed in groups of 3-5 per cage, maintained on a regular 12 h light/dark cycle with *ad libitum* access to food and water.

Two independent animal experiments were conducted in this study: (1) stereotaxic injection of AAV for neuron-specific overexpression of PRMT3, and (2) systemic administration of the PRMT3 inhibitor SGC707.

For the AAV injection experiment, vectors were constructed as previously described.[3] 5-month-old mice received bilateral injections targeting the medial entorhinal cortex (MEC) using the following stereotaxic coordinates relative to bregma: anteroposterior (AP) −4.85 mm, mediolateral (ML) ±3.45 mm, and dorsoventral (DV) −3.30 mm. A total volume of 0.4 μL was delivered per hemisphere. The WT+Vehicle group (n = 5) and PS19+Vehicle group (n = 5) received bilateral MEC injections of sterile PBS (0.4 μL) to control for surgical and injection-related variables in WT and disease model mice, respectively. The PS19+Vector group (n = 5) received AAV9-hSyn-GFP (1.44×10^{13} vg/mL) to control for potential effects of AAV delivery without transgene expression. The treatment group, PS19+PRMT3 (n = 5), received AAV9-hSyn-*Prmt3*-GFP (1.61×10^{13} vg/mL) to achieve neuron-specific overexpression of PRMT3 in the MEC.

For the SGC707 treatment experiment, the 8-month-old mice were distributed into three groups: WT+Saline group (n = 5), PS19+Saline group (n = 8) and PS19+SGC707 group (n = 13). Specifically, WT mice injected with saline were used as the normal baseline control group. PS19 mice injected with saline were designated as the vehicle treatment group, serving as the negative control for the disease model. PS19 mice injected with SGC707 constituted the treatment group. The vehicle group was essential for assessing the specific effects of SGC707 treatment in the context of the disease. SGC707 was prepared according to the manufacturer’s protocol, and mice were injected intraperitoneally (i.p.) with SGC707 at a dose of 20 mg/kg every other day (q.o.d.) for 60 d. Saline served as a control.

Following the behavioral tests, mice were anesthetized with 0.1% pentobarbital solution and perfused with PBS. Each brain was bisected into two hemispheres. The left hemisphere was fixed for subsequent immunofluorescence (IF) or Golgi staining, while the right hemisphere was lysed for protein and RNA extraction. All animal procedures conducted in this study were reviewed and approved by the Institutional Animal Care and Use Committee in the Chinese Academy of Medical Sciences, Institute of Basic Medical Sciences (China).

Human brain samples

All postmortem human brain tissues were obtained from the National Human Brain Bank for Development and Function, housed at the Chinese Academy of Medical Sciences and Peking Union Medical College, Beijing, China. Donor information specific to each experiment involving human brain tissues is summarized in Table S1. All procedures involving human tissues were conducted in strict accordance with the Standardized Operational Protocol of the National Human Brain Bank for

Development and Function to minimize the impact of sample handling on data integrity.[4] Specifically, frozen brain tissues were used for transcriptomic, WB, and qPCR analyses, while formalin-fixed tissues were utilized for IHC and IF experiments. By adhering to rigorous preservation and handling standards, we maximized data quality and consistency, thereby minimizing potential biases arising from variations in tissue preservation methods. The present study was approved by the Institutional Review Board of the Institute of Basic Medical Sciences, Chinese Academy of Medical Sciences (Approval Number: 009-2014, 031-2017 and 2022125). Specifically, the classification of primary age-related tauopathy (PART) and Alzheimer's disease (AD) groups was based on neuropathological examination, independent of clinical features. PART was defined according to the criteria proposed by Crary et al.,[5] characterized by phosphorylated tau pathology in the form of neurofibrillary tangles (NFTs) and/or pathologic neurites in the absence of Aβ neuritic plaques or with only focal diffuse Aβ plaques. PART cases were further subdivided into "definite" PART, defined as having no Aβ deposition (Thal phase "A" = 0, Braak stage ≤ IV, and CERAD neuritic plaque "C" = 0), and "possible" PART, defined as having diffuse Aβ plaques (Thal phase "A" = 1-2, Braak stage ≤ IV, and CERAD neuritic plaque "C" = 0). In this study, only cases classified as "definite" PART were included.[6]

For AD brains, the inclusion criteria were based on the National Institute on Aging-Alzheimer's Association (NIA-AA) guidelines for AD neuropathologic change.[6]

H&E staining

To assess the toxicity of SGC707, frozen sections of hearts, livers, spleens, lungs, kidneys from mice were stained with Hematoxylin-Eosin (HE) stain Kit (Solarbio, G1120) following the manufacturer's instructions and evaluated using an optical microscope.

IHC staining

Paraffin-embedded human brain tissue sections were subjected to heating at 60 °C for 20 min, followed by deparaffinization in xylene and dehydration through a gradient series of ethanol solutions. Antigen retrieval was performed using heated citrate buffer for 10 min. After blocking endogenous peroxidase activity, the sections were incubated with anti-PRMT3 primary antibody (1:50, Abcam, ab191562) or anti-phospho-Tau primary antibody (Ser202, Thr205) (AT8; 1:250, Invitrogen, MN1020) at 4 °C for 16 h. Subsequently, the sections were treated with a reaction enhancer reagent and goat anti-rabbit immunoglobulin G (IgG) polymer (ZSGB-BIO, PV-9001). The signal was then detected using diaminobenzidine. The slides were rinsed with PBS three times prior to each incubation step. After dehydration, clearing, and mounting, images of the sections were captured.

IF staining

For paraffin-embedded sections (15 μm) of the human brain EC region, the sections were first deparaffinized in xylene and rehydrated through a graded ethanol series. Membrane permeabilization was achieved by incubating the sections with 0.3% Triton X-100 for 30 min to facilitate antibody access to intracellular antigens. Antigen retrieval was performed by heating the sections to 98 °C for 10 min in 0.01M trisodium citrate, followed by incubation with 0.5% sodium borohydride to reduce autofluorescence. After rinsing with PBS, the sections were blocked in 10% goat serum containing 1% BSA for 30 min at room temperature, and then incubated overnight at 4 °C with the primary antibodies. The next day, sections were incubated for 1 h at room temperature with AlexaFluor488-conjugated goat

anti-rabbit IgG (1:200, Invitrogen, A-11034) and AlexaFluor594-conjugated goat anti-mouse IgG (1:200, Invitrogen, A-11037). To minimize lipid autofluorescence, sections were sequentially treated with ethanol and Sudan Black. After removing Sudan Black with 70% ethanol, sections were mounted in fluorescent mounting medium containing DAPI (ZSGB-BIO, ZLI-9557), and fluorescence signals were captured using a Leica DMi8 microscope.

For IF staining of cells, SH-SY5Y cells or mouse primary neurons were fixed with 4% paraformaldehyde and permeabilized with 0.3% Triton X-100 in PBS. The cells were then incubated overnight at 4 °C with the primary antibodies. Following primary antibody incubation, the samples were treated with species-specific fluorescent secondary antibodies. For dendritic spine visualization, Alexa Fluor™ 488 phalloidin (1:400, Thermo Fisher, A12379) was applied. Finally, the samples were mounted with fluorescence mounting medium containing DAPI and imaged using a Leica DMi8 or Stellaris 5 confocal microscope.

For mouse brain cryosections, the hemispheres were embedded in Optimal Cutting Compound (O.C.T., Sakura Finetek, 4583) following dehydration through a graded sucrose solution. Sections of 15 μm thickness were cut using a cryostat. The sections were fixed in 4% paraformaldehyde for 30 min, permeabilized with 0.3% Triton X-100 for 5 min, and blocked with 5% BSA for 1 h. Primary antibodies were incubated with the sections overnight at 4 °C. After incubation with fluorescent secondary antibodies and a 5-min DAPI staining, the sections were imaged using a Leica DMi8 microscope. To stain tau aggregates, a 0.05% Thioflavin S (MedChemExpress, HY-D0972) solution prepared in 50% ethanol was applied for 8 min, followed by 15 s in 80% ethanol, and washed three times with ddH_2O. The samples were mounted and imaged using a Leica DMi8 microscope. Neuronal apoptosis was detected using a terminal deoxynucleotidyl transferase-mediated dUTP-biotin nick end labeling (TUNEL) assay (Beyotime, C1086), followed by staining with anti-NeuN (1:200, Abcam, ab104224) and fluorescent secondary antibodies. The sections were mounted and visualized using a Leica DMi8 microscope. To assess degenerating mature neurons, Fluoro-Jade C (FJC) staining was performed using a ready-to-dilute staining kit (Biosensis, BSS-TR-100-FJT), according to the manufacturer's protocol.

Golgi staining

Golgi staining was conducted using the FD Rapid GolgiStain Kit (FD Neurotechnology, PK401) following the manufacturer's protocol. The left hemisphere of the mouse brain was immersed in a 1:1 mixture of Solutions A and B for 24 h, after which the tissue was transferred to fresh AB solution and incubated for 2 weeks. The brain was then soaked in Solution C for 3 d. Subsequently, the tissue was sectioned into 100 μm thick slices using a cryotome. The sections were air-dried in the dark, rinsed twice with double-distilled water (ddH2O) for 4 min each, and stained with a mixture of Solutions D, E, and ddH2O (1:1:2) for 10 min. After another two 4-minute rinses in ddH2O, the sections were dehydrated through graded ethanol series (50%, 75%, and 95%) for 4 min each, followed by immersion in anhydrous ethanol for 4 cycles of 4 min. Finally, the sections were cleared in xylene for 30 min. Images were captured using a Leica DM6B microscope. For the analysis of dendritic spine morphology, 8 random fields of view were selected per group, and 5 dendrites were randomly chosen from each field. Images were processed using ImageJ software by converting them to grayscale and adjusting the contrast to enhance the visualization of dendritic spines. Spines were classified into four categories—mushroom, stubby, thin, and filopodium—based on established morphological criteria.[7]

Immunoblotting assays

Western Blot and Dot Blot were utilized to detect protein expression level. Protein samples were extracted using RIPA buffer (Solarbio, R0010) supplemented with 1% protease inhibitors (Roche, 04693132001) and phosphatase inhibitors (Roche, 04906837001), and their concentrations were determined using BCA Protein Assay Kit (Thermo Scientific, 23225).

For western blot analysis, 15 μg of each protein sample was separated by SDS-PAGE on a 10-15% polyacrylamide gel under standard conditions. Following electrophoresis, proteins were transferred onto polyvinylidene fluoride (PVDF) membranes with pore sizes of 0.2 or 0.45 μm, selected based on the molecular weights of the target proteins. To ensure accurate normalization, internal control blots were probed at room temperature for 1 h with HRP-conjugated β-actin antibody (Proteintech, HRP-60008, 1:10000) or HRP-conjugated GAPDH antibody (Proteintech, HRP-60004, 1:10000). Detection was performed using the Immobilon Western Chemiluminescent HRP Substrate (Millipore, WBKLS0500) and visualized on a ChemiDoc XRS+ Gel Imaging System (BIO-RAD). Blots targeting specific proteins were incubated overnight at 4 °C with the primary antibodies. Subsequently, membranes were washed and incubated with HRP-conjugated secondary antibodies for 1 h at room temperature. Protein bands were detected using the Immobilon Western Chemiluminescent HRP Substrate and visualized using the ChemiDoc XRS+ Gel Imaging System. The full length uncropped original western blots and gray value were supplied in Supporting Information.

Cell viability analysis

Cell viability was measured using CellTiter-Glo 3D Viability Assay (Promega, G9681), which quantifies ATP levels as an indicator of metabolically active cells. SH-SY5Y cells were cultured on in opaque-walled multiwell plates, and the viability analysis was conducted according to the manufacturer's protocol. The assay was performed using a microplate reader, with cell viability determined based on an ATP standard curve.

Mitochondrial Mass Assessment

MitoTracker-Green (MTG; Beyotime, C1048) is a mitochondrial membrane potential (MMP)-independent dye used to monitor mitochondria. After treatment, cells were stained with MTG (100 nM) and analyzed using a Cytoflex flow cytometer (Beckman Coulter).

MMP Measurement

JC-1 (MedChemExpress, HY-15534) is a lipophilic cationic dye that accumulates in the inner mitochondrial membrane in response to MMP. In healthy mitochondria, JC-1 forms red fluorescent aggregates, while in damaged mitochondria, it remains as green fluorescent monomers. SH-SY5Y cells were stained with JC-1 for 30 min at 37 °C and analyzed via flow cytometry.

Mitochondrial Morphology Assessment

SH-SY5Y cells from various treatment groups were collected and fixed with 2.5% glutaraldehyde. After post-fixation in osmium tetroxide, the samples were dehydrated through a gradient series and embedded in epoxy resin. Ultrathin sections were prepared, stained, and imaged using a JEM-1400Plus transmission electron microscope (JEOL) to assess mitochondrial morphology.

Cellular ROS Analysis

Mitochondrial superoxide production in damaged mitochondria was quantified using MitoSOX Red (Beyotime, S0061) and visualized under a Leica DMi8 microscope.

Y-Maze Test

The Y-maze test is used to evaluate short-term spatial memory in mice. The apparatus consists of a Y-shaped structure with three identical arms (300×50×120 mm each) arranged at a 120° angle. The arms are labeled as the start arm, the other arm, and the novel arm. The test consists of two phases: a training phase and a testing phase, separated by a 1-hour intertrial interval. During the training phase, mice were placed in the start arm, facing the central zone, and allowed to explore two arms for 10 min, while the novel arm was blocked. In the testing phase, mice had full access to all three arms for 8 min. The number of arm entries, time spent in the novel arm, and velocity were recorded and analyzed. To ensure consistency, the maze was cleaned with 75% ethanol between trials.

Step-Through Passive Avoidance Test

The step-through passive avoidance test assesses non-spatial memory retention in mice by evaluating their avoidance behavior in a two-compartment box (light and dark). During the acquisition phase, mice were confined to the light compartment for 60 s. After this adaptation period, the barrier separating the compartments was removed, allowing the mice to freely explore both compartments for 5 min. As mice naturally prefer darker environments, they would enter the dark compartment, at which point an electric foot shock (5 s) was administered when all four paws were inside. The retention phase occurred 24 h later, during which the mouse was placed back in the light compartment. The latency to re-enter the dark compartment was recorded, and no shock was administered during the retention phase.

Morris Water Maze Test

The Morris water maze test was conducted to evaluate spatial memory and learning in mice. The test utilized a circular pool (150 cm in diameter) divided into four quadrants. Mice underwent training three times daily for five consecutive days to locate a submerged escape platform (1 cm below the water surface), placed in the center of quadrant I. During each training session, mice were released from different quadrants, facing the wall, and were allowed to search for the platform for up to 90 s. If a mouse failed to find the platform within the time limit, it was guided to the platform and remained there for 30 s. On the sixth day, a probe test was conducted, during which the platform was removed and the mouse, starting from quadrant III, swam freely for 90 s. Data collected included the path taken, the number of platform zone crossings, time spent in the target quadrant, and swimming velocity

Molecular docking

The Colabfold was used to predict the structures of PRMT3-H4 protein complex. The input protein sequences spaning from F221 to Q530 of PRMT3 and from S1 to G9 of H4. MMseqs2-based homology search server was used to build diverse MSAs and to find templates. The pair_mode was unpaired_paired, which meant pair sequences from same species and unpaired MSA. Alphafold2 multimer v2 was used for complex prediction. The advanced settings were set as follows: num_recycles=3, recycle_early_stop_tolerence=0.5, relax_max_iterations=200, pairing_strategy= greedy. The docking results were visualized using PyMOL.

RNA-seq and ChIP-seq

RNA-seq was performed by Shanghai Biotechnology Corporation (Shanghai, China). Total RNA was extracted using the MJzol Animal RNA Isolation Kit (Majorivd) following the manufacturer's standard protocol. The extracted RNA was then purified using the RNAClean XP Kit (Beckman Coulter) and treated with RNase-Free DNase to remove residual genomic DNA. RNA integrity was assessed using either the Agilent 2100 Bioanalyzer or Agilent 4200 TapeStation (Agilent Technologies). RNA concentration and purity were quantified using the Qubit 2.0 Fluorometer (Thermo Fisher Scientific) and the NanoDrop ND-2000 Spectrophotometer (Thermo Fisher Scientific). Subsequent mRNA isolation and fragmentation were performed on the purified total RNA, followed by first- and second-strand cDNA synthesis. The cDNA was subjected to end-repair, 3′-end adenylation, adaptor ligation, and enrichment to construct the mRNA sequencing libraries. Library concentrations were quantified using the Qubit 2.0 Fluorometer (Thermo Fisher Scientific), and fragment size distributions were assessed using the Agilent 4200 TapeStation (Agilent Technologies). Sequencing was performed on an Illumina NovaSeq 6000 platform in paired-end 150 bp mode (PE150).

ChIP-seq was performed by Seqhealth Technology Co., Ltd (Wuhan, China). SH-SY5Y Cells were fixed in 1% formaldehyde for 10 minutes at room temperature, followed by the addition of 0.125 M glycine to quench the crosslinking reaction, allowing the mixture to stand for 5 min. The cells were then treated with a cell lysis buffer, and the nuclei were collected by centrifugation at 2,000 g for 5 min. Subsequently, the nuclei were treated with nuclear lysis buffer and sonicated to fragment the chromatin DNA. DNA from both input and immunoprecipitated (IP) samples was extracted using the phenol-chloroform method. High-throughput DNA sequencing libraries were prepared using the VAHTS Universal DNA Library Prep Kit for Illumina V3. Library fragments of 200-500 base pairs were enriched, quantified, and sequenced on a DNBSEQ-T7 sequencer.

Bioinformatics analysis

The standard bioinformatics analyses, including differential expression analysis, GO analysis, GSEA analysis, and KEGG pathway analysis, were all performed using the SRplot online tool (http://www.bioinformatics.com.cn/srplot). Based on RNA-seq data, differentially expressed genes were identified using the following criteria: For human brain samples, genes with $|\log_2 FC| > 0.5$ and adjusted P-value < 0.0001 were considered significant. For cell RNA-seq data, differentially expressed genes were defined by a fold change greater than 1.3 or less than 0.76, with an adjusted P-value of less than 0.05. Differential expression analysis was performed using DESeq2, with default parameters for both human brain and cell RNA-seq datasets. Differential peak analysis of H4R3me2a ChIP-seq data between conditions was performed using the csaw package, which is based on the edgeR framework. This approach allows for accurate identification of differential peaks by accounting for biases such as library size and sequencing depth. For each peak, a threshold of $|\log_2 FC| > 0.5$ applied to define significant differences in H4R3me2a binding.

The RNA-Seq dataset from 45 EC samples was analyzed using the Weighted Gene Co-expression Network Analysis (WGCNA) algorithm. The WGCNA::blockwiseModules() function was applied with the following parameters for network construction: a soft-threshold power of 10, a minimum module size of 15, a signed network using partitioning around medoids while preserving the dendrogram structure, and a reassignment threshold of $P < 0.05$. Clustering was performed within a single block. Modules were

arbitrarily assigned colors for visualization, and genes that were not assigned to a specific module were represented in gray. Cell-type-specific marker genes were obtained from the PanglaoDB database.[8] The number of identified genes was counted for each module, and Fisher's exact test was employed to assess whether module genes were significantly enriched for specific cell-type terms. Enrichment results, along with the WGCNA correlation results, were visualized using the circlize R package. Cytoscape v3.10.1 was utilized to construct a PPI network for analyzing candidate gene-encoded proteins, with the MCODE plug-in employed using default parameters to visualize significant gene clusters and identify the hub gene.

Quantitative real-time PCR (qRT-PCR)

Total RNA was extracted from cells or brain tissues using TRIzol reagent (Invitrogen, 15596018). For mRNA analysis, reverse transcription and cDNA amplification were performed using the One Step TB Green® PrimeScript™ PLUS RT-PCR Kit (Takara, RR096A), with GAPDH serving as the internal control. Relative gene expression changes were calculated using the $2^{-\Delta\Delta Ct}$ method, normalized to GAPDH mRNA levels. For microRNA analysis, reverse transcription and qRT-PCR were conducted using the Bulge-Loop™ miRNA qRT-PCR Starter Kit (RiboBio, C10211-2) on a Bio-Rad CFX96 system. Relative quantification was performed using the ΔΔCt method, where $\Delta Ct = Ct_{\text{targeted miRNA}} - Ct_{U6}$. Bulge-loop miRNA primers were designed and purchased from RiboBio Corporation (Guangzhou, China), and their sequences are proprietary. The qRT-PCR primers used in this study are listed in Table S5.

[1] a)D. Luo, J. Li, H. Liu, J. Wang, Y. Xia, W. Qiu, N. Wang, X. Wang, X. Wang, C. Ma, W. Ge, *Adv Sci (Weinh)* **2023**, 10, e2300876; b)H. Liu, J. Li, X. Wang, S. Luo, D. Luo, W. Ge, C. Ma, *J Neuroinflammation* **2024**, 21, 84.

[2] Y. Sun, Y. Guo, X. Feng, M. Jia, N. Ai, Y. Dong, Y. Zheng, L. Fu, B. Yu, H. Zhang, J. Wu, X. Yu, H. Wu, W. Kong, *J Neuroinflammation* **2020**, 17, 72.

[3] F. Liu, L. Zhang, S. Su, Y. Fang, X. S. Yin, H. Cui, J. Sun, Y. Xie, C. Ma, *Adv Sci (Weinh)* **2023**, 10, e2205397.

[4] W. Qiu, H. Zhang, A. Bao, K. Zhu, Y. Huang, X. Yan, J. Zhang, C. Zhong, Y. Shen, J. Zhou, X. Zheng, L. Zhang, Y. Shu, B. Tang, Z. Zhang, G. Wang, R. Zhou, B. Sun, C. Gong, S. Duan, C. Ma, *Neuroscience bulletin* **2019**, 35, 270.

[5] J. F. Crary, J. Q. Trojanowski, J. A. Schneider, J. F. Abisambra, E. L. Abner, I. Alafuzoff, S. E. Arnold, J. Attems, T. G. Beach, E. H. Bigio, N. J. Cairns, D. W. Dickson, M. Gearing, L. T. Grinberg, P. R. Hof, B. T. Hyman, K. Jellinger, G. A. Jicha, G. G. Kovacs, D. S. Knopman, J. Kofler, W. A. Kukull, I. R. Mackenzie, E. Masliah, A. McKee, T. J. Montine, M. E. Murray, J. H. Neltner, I. Santa-Maria, W. W. Seeley, A. Serrano-Pozo, M. L. Shelanski, T. Stein, M. Takao, D. R. Thal, J. B. Toledo, J. C. Troncoso, J. P. Vonsattel, C. L. White, 3rd, T. Wisniewski, R. L. Woltjer, M. Yamada, P. T. Nelson, *Acta Neuropathol* **2014**, 128, 755.

[6] T. J. Montine, C. H. Phelps, T. G. Beach, E. H. Bigio, N. J. Cairns, D. W. Dickson, C. Duyckaerts, M. P. Frosch, E. Masliah, S. S. Mirra, P. T. Nelson, J. A. Schneider, D. R. Thal, J. Q. Trojanowski, H. V. Vinters, B. T. Hyman, A. National Institute on, A. Alzheimer's, *Acta Neuropathol* **2012**, 123, 1.

[7] W. C. Risher, T. Ustunkaya, J. Singh Alvarado, C. Eroglu, *PLoS One* **2014**, 9, e107591.

[8] O. Franzén, L. M. Gan, J. L. M. Björkegren, *Database : the journal of biological databases and curation* **2019**, 2019.

The orginal uncropped images and normalized gray values of western blot

Figure 1K

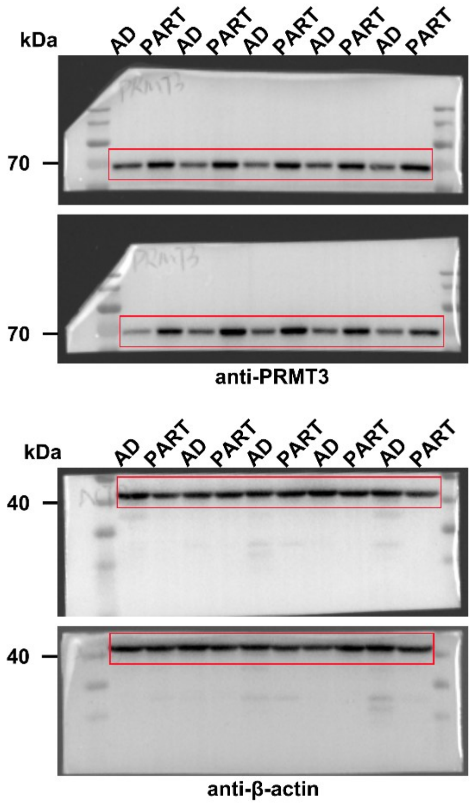

Figure 1K

PRMT3/β-actin	
**AD**	**PART**
0.4971	0.9826
0.6224	0.9514
1.1254	1.5967
0.3793	0.8884
0.3797	1.0441
0.4584	1.0676
0.8126	1.1960
0.7910	1.4146
0.8718	1.2756
0.7825	1.4176

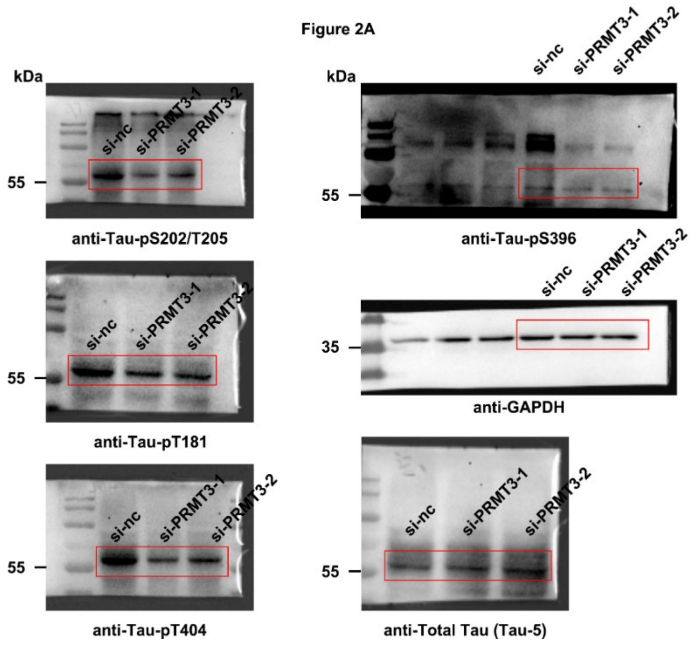

Figure 2A

p-tau S202 T205/total tau		
**si-nc**	**si-PRMT3-1**	**si-PRMT3-2**
1.8186	0.9604	1.1649
1.8043	0.9490	1.1521
1.9139	0.9385	0.9986
p-tau pT181/total tau		
**si-nc**	**si-PRMT3-1**	**si-PRMT3-2**
2.0066	1.2950	1.0263
1.9613	1.3543	1.1636
1.8806	1.0294	0.9662
p-tau pS404/total tau		
**si-nc**	**si-PRMT3-1**	**si-PRMT3-2**
2.1438	1.2571	1.3246
2.2711	1.1794	1.1335
2.2802	1.3248	1.2761
p-tau pS396/total tau		
**si-nc**	**si-PRMT3-1**	**si-PRMT3-2**
0.9461	0.6251	0.3727
0.8819	0.6735	0.4270
0.8239	0.7091	0.4473

Figure 2C

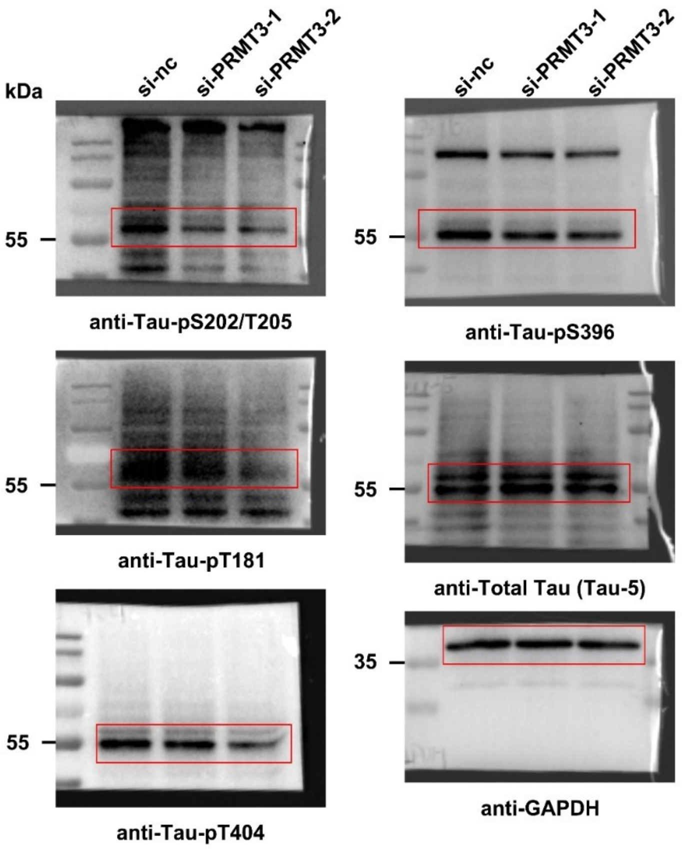

Figure 2C

p-tau S202 T205/total tau		
**si-nc**	**si-PRMT3-1**	**si-PRMT3-2**
1.3052	0.9090	0.9100
1.2180	0.8269	0.8931
1.4134	0.6100	1.0154
p-tau pT181/total tau		
**si-nc**	**si-PRMT3-1**	**si-PRMT3-2**
1.4416	1.1338	0.8682
1.2939	0.9796	0.7312
1.3537	1.0870	0.9978
p-tau pS404/total tau		
**si-nc**	**si-PRMT3-1**	**si-PRMT3-2**
1.2478	0.8953	0.3903
1.2787	0.8307	0.4121
1.3558	0.7342	0.4686
p-tau pS396/total tau		
**si-nc**	**si-PRMT3-1**	**si-PRMT3-2**
1.5081	1.1215	0.8928
1.7150	1.2982	1.0840
1.5193	1.2211	0.9930

Figure 2E

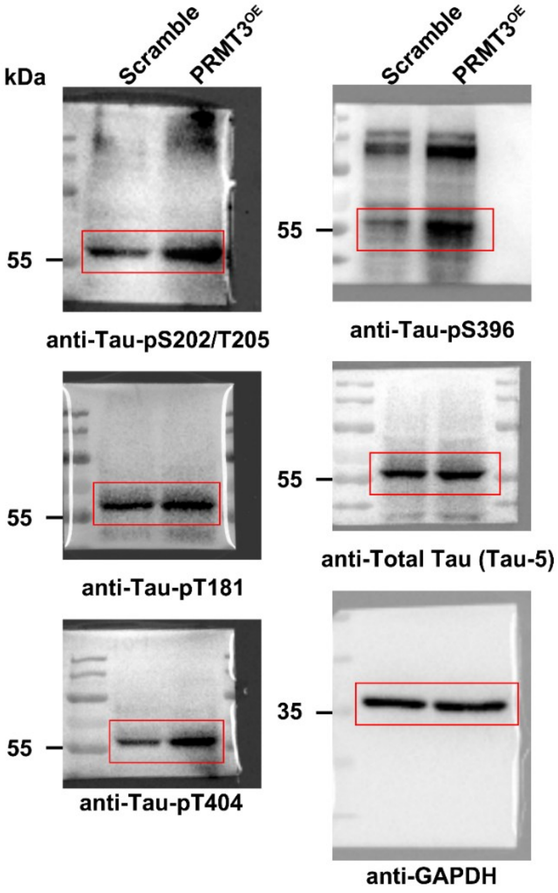

Figure 2E

p-tau S202 T205/total tau	
**Scramble**	**PRMT3^{OE}**
0.6716	0.8257
0.5918	0.7723
0.5630	0.7761
p-tau pT181/total tau	
**Scramble**	**PRMT3^{OE}**
0.8470	0.9277
0.9268	1.0096
0.8665	1.0036
p-tau pS404/total tau	
**Scramble**	**PRMT3^{OE}**
0.7301	1.0301
0.7772	1.0817
0.6477	1.0413
p-tau pS396/total tau	
**Scramble**	**PRMT3^{OE}**
0.5126	0.8891
0.4076	0.9270
0.4969	0.8539

Figure 2F

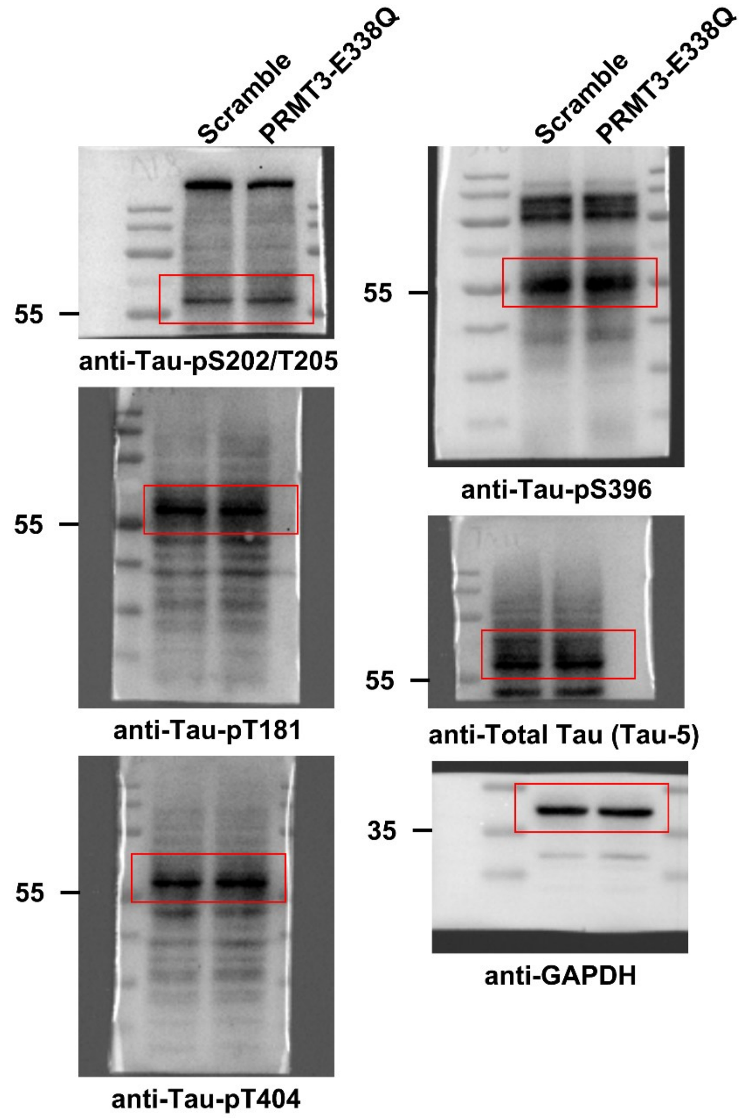

Figure 2F

Scramble	PRMT3-E338Q
p-tau S202 T205/total tau	
**Scramble**	**PRMT3-E338Q**
0.4645	0.4591
0.4406	0.4666
0.3700	0.3230
p-tau pT181/total tau	
**Scramble**	**PRMT3-E338Q**
1.6271	1.5194
1.6396	1.5800
1.3063	1.2142
p-tau pS404/total tau	
**Scramble**	**PRMT3-E338Q**
0.7696	0.8108
0.7483	0.8183
0.6354	0.6592
p-tau pS396/total tau	
**Scramble**	**PRMT3-E338Q**
2.4864	2.4333
2.4660	2.5806
1.9818	1.9633

Figure 2G

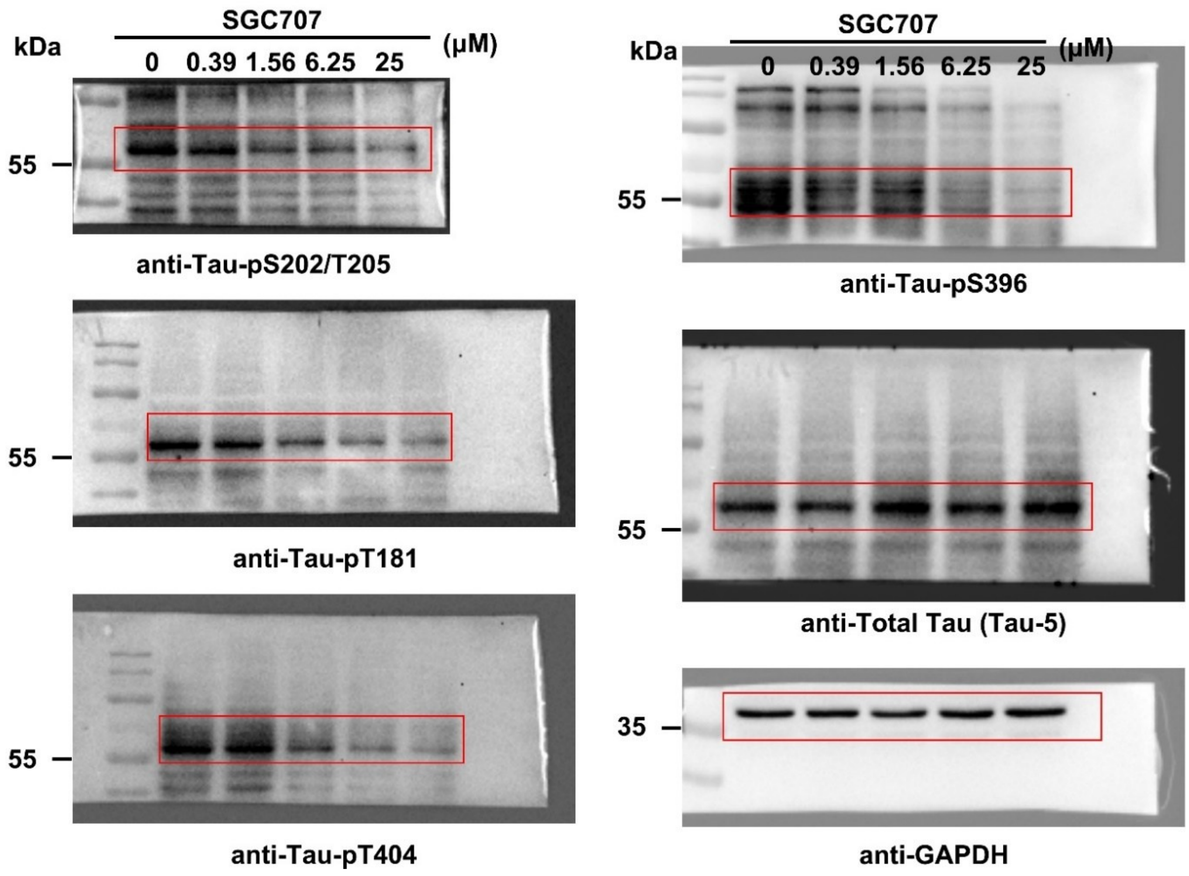

Figure 2G

p-tau S202 T205/total tau				
**0μM**	**0.39μM**	**1.56μM**	**6.25μM**	**25μM**
2.6569	2.4897	1.6744	1.6839	1.2099
2.6347	2.4612	1.6509	1.6763	1.1925
2.7345	2.5569	1.7233	1.7310	1.2091
p-tau pT181/total tau				
**0μM**	**0.39μM**	**1.56μM**	**6.25μM**	**25μM**
1.4717	1.2800	0.6275	0.5577	0.4830
1.5306	1.4350	0.6017	0.6034	0.4933
1.5220	1.4200	0.6120	0.6145	0.4110
p-tau pS404/total tau				
**0μM**	**0.39μM**	**1.56μM**	**6.25μM**	**25μM**
1.3671	1.3666	0.9190	0.5635	0.3888
1.4194	1.2895	0.8846	0.5354	0.4180
1.4636	1.3659	0.8916	0.5533	0.4066
p-tau pS396/total tau				
**0μM**	**0.39μM**	**1.56μM**	**6.25μM**	**25μM**
1.6314	0.9229	0.7838	0.5035	0.1763
1.7710	1.1736	0.7484	0.5171	0.1627
1.7160	1.0718	0.7250	0.4826	0.1673

Figure 2I

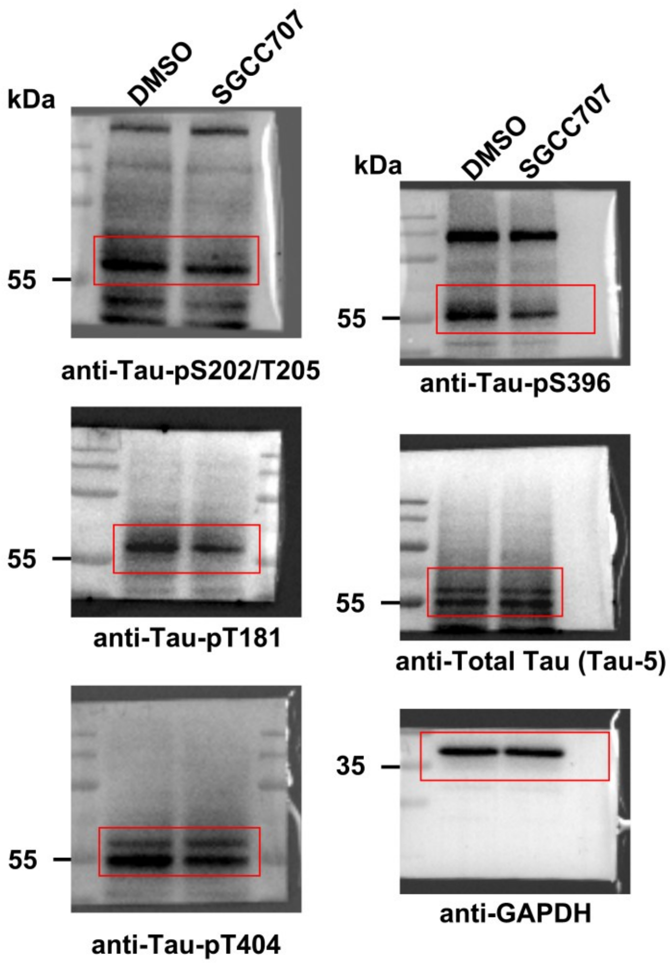

Figure 2I

p-tau S202 T205/total tau	
**DMSO**	**SGC707**
1.3139	0.8906
1.2788	0.8889
1.2955	0.9160
p-tau pT181/total tau	
**DMSO**	**SGC707**
1.3490	0.9681
1.3825	0.9121
1.4413	1.0303
p-tau pS404/total tau	
**DMSO**	**SGC707**
1.5066	0.8887
1.3863	0.9184
1.4474	0.9312
p-tau pS396/total tau	
**DMSO**	**SGC707**
1.2825	0.6213
1.2788	0.5913
1.2958	0.6490

Figure 2K

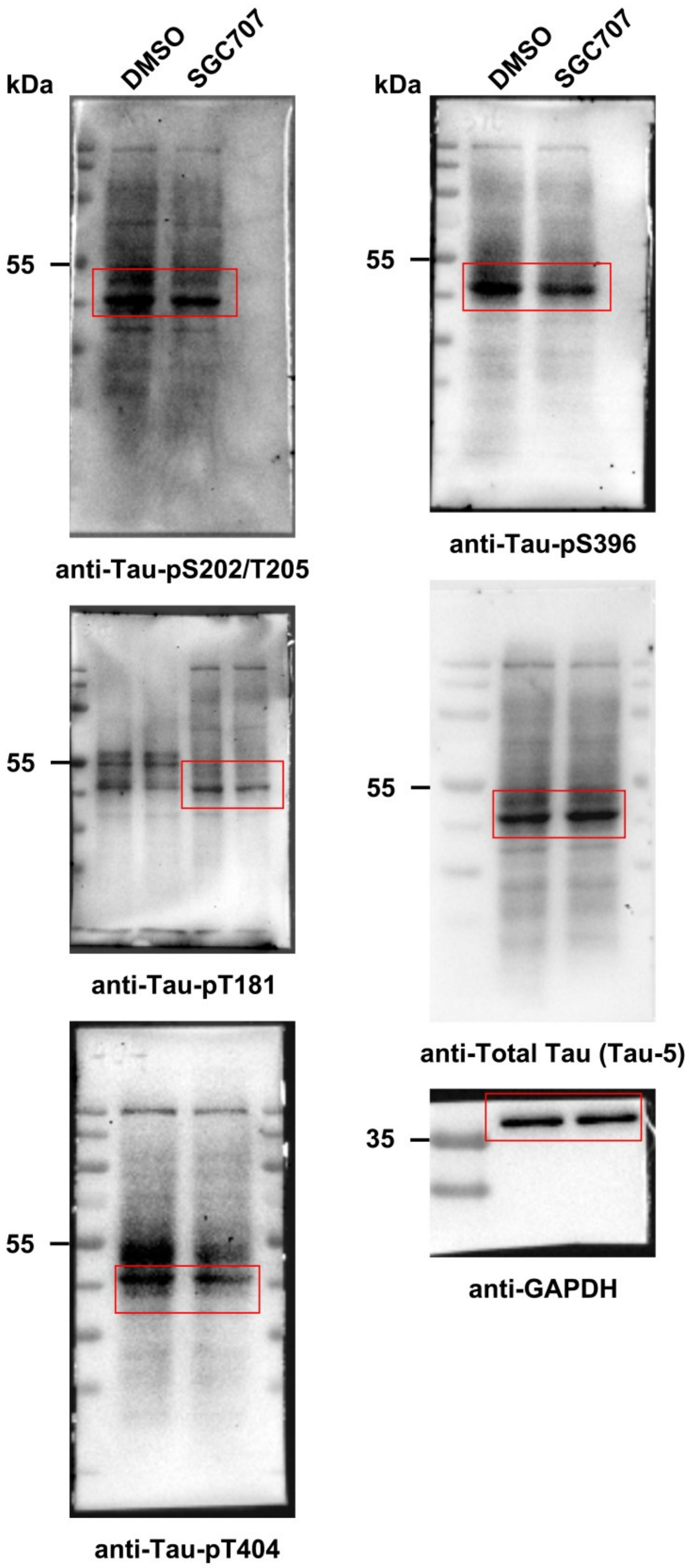

Figure 2K

p-tau S202 T205/total tau	
**DMSO**	**SGC707**
0.9800	0.7356
1.0364	0.6310
1.0053	0.6263
p-tau pT181/total tau	
**DMSO**	**SGC707**
0.8918	0.5620
0.9533	0.5473
0.8856	0.5206
p-tau pS404/total tau	
**DMSO**	**SGC707**
0.8080	0.6608
0.8655	0.7215
0.8909	0.7103
p-tau pS396/total tau	
**DMSO**	**SGC707**
0.9154	0.7220
0.8725	0.6675
0.9134	0.7133

Figure S2A and S2C

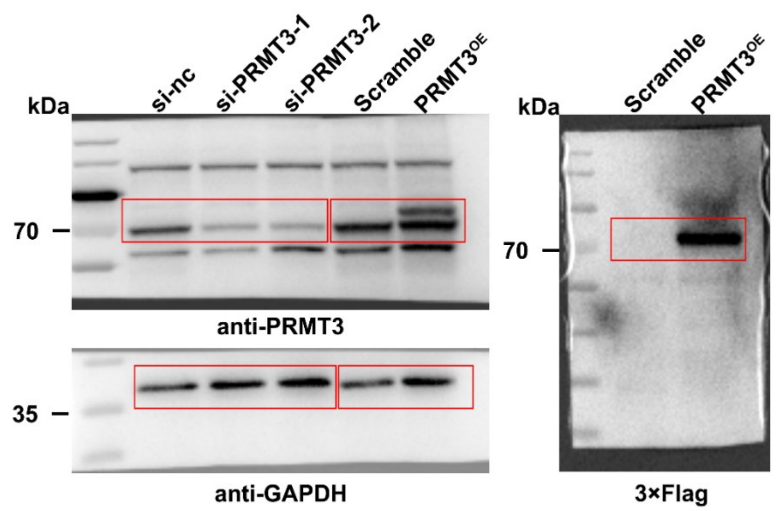

Figure S2B

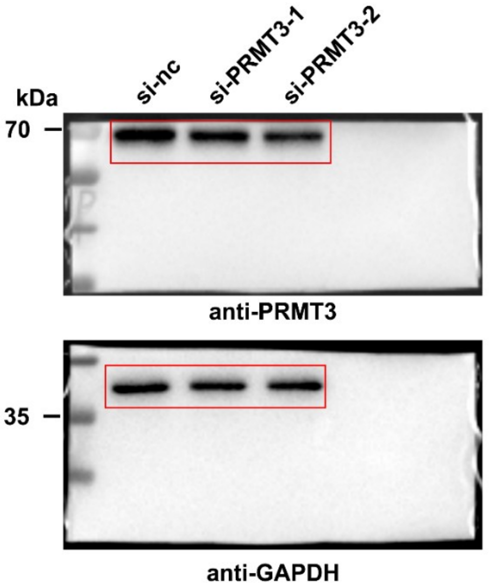

Figure S2F

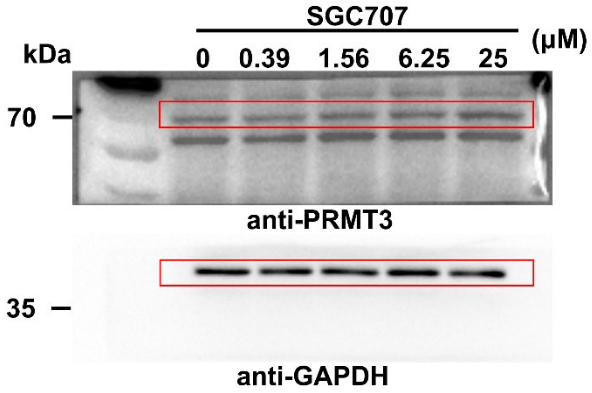

Figure S2F

PRMT3/GAPDH				
**0μM**	**0.39μM**	**1.56μM**	**6.25μM**	**25μM**
0.5838	0.6306	0.6079	0.5632	0.6402
0.6044	0.6554	0.6396	0.6039	0.6679
0.5489	0.5857	0.5817	0.5392	0.6030

Figure 3C

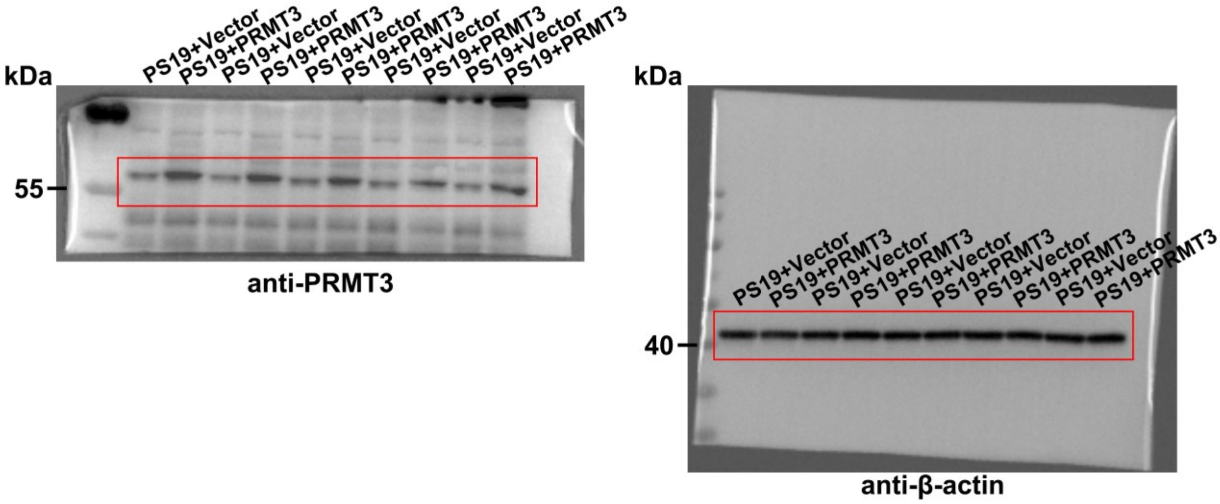

Figure 3C

PRMT3/β-actin	
**PS19+Vector**	**PS19+PRMT3**
0.1450	0.3087
0.1161	0.3014
0.1384	0.2453
0.1201	0.1933
0.1215	0.2908

Figure 3D

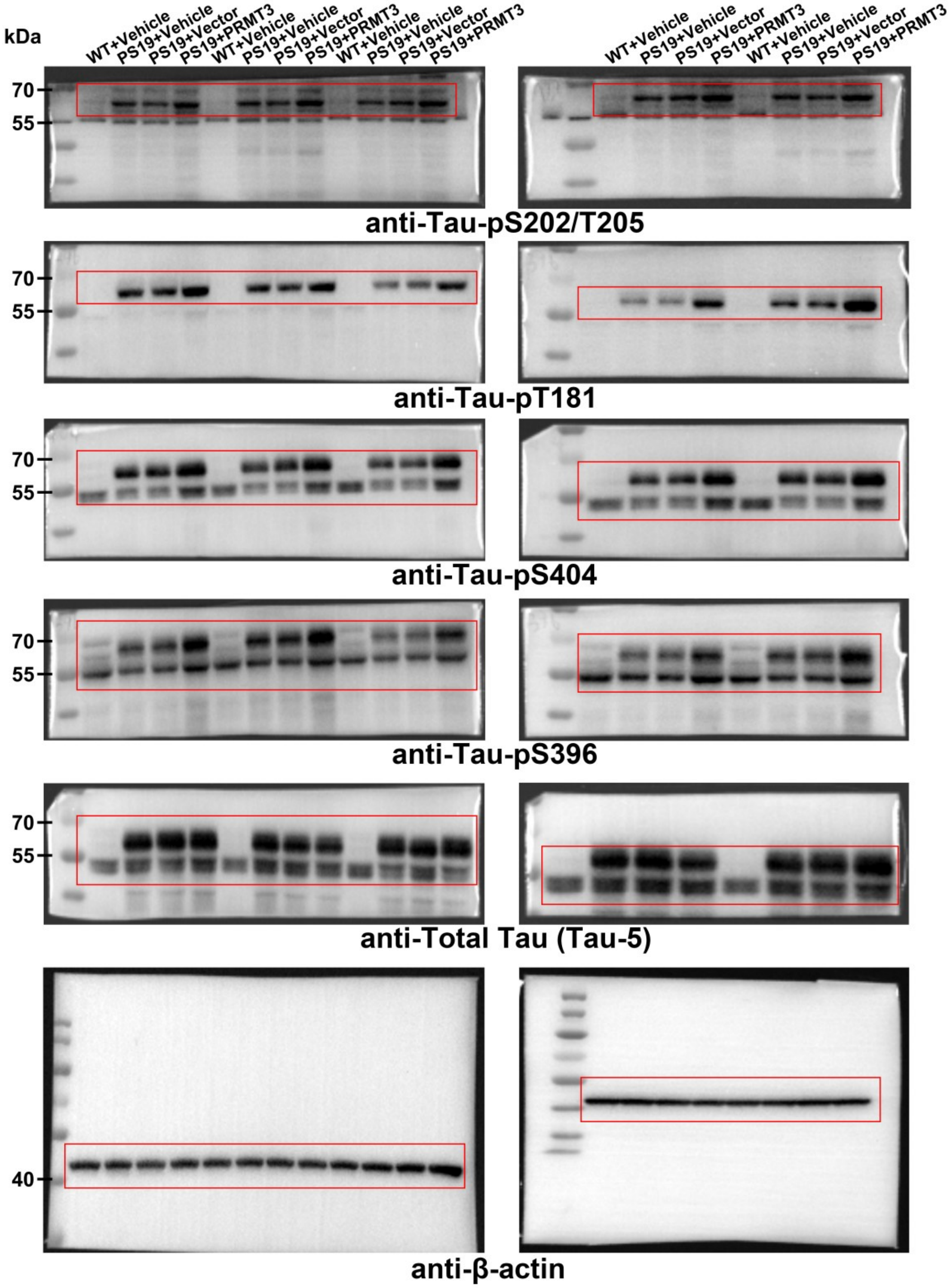

Figure 3D

p-tau S202 T205/total tau			
**WT+Vehicle**	**PS19+Vehicle**	**PS19+Vector**	**PS19+PRMT3**
0.0000	0.3322	0.2540	0.4626
0.0000	0.3520	0.3693	0.5212
0.0000	0.2591	0.2854	0.3702
0.0000	0.4214	0.4054	0.6435

0.0000	0.4477	0.3526	0.4760

p-tau pT181/total tau			
**WT+Vehicle**	**PS19+Vehicle**	**PS19+Vector**	**PS19+PRMT3**
0.0000	0.5682	0.5532	0.7793
0.0000	0.5633	0.5993	0.9003
0.0000	0.3470	0.3996	0.6273
0.0000	0.2842	0.2830	0.9372
0.0000	0.5539	0.5906	0.9106

p-tau pS404/total tau			
**WT+Vehicle**	**PS19+Vehicle**	**PS19+Vector**	**PS19+PRMT3**
0.0000	0.7460	0.7200	0.9909
0.0000	0.6751	0.8401	1.2024
0.0000	0.5989	0.5626	0.9167
0.0000	0.6462	0.6718	1.3727
0.0000	0.8569	0.8474	1.1434

p-tau pS396/total tau			
**WT+Vehicle**	**PS19+Vehicle**	**PS19+Vector**	**PS19+PRMT3**
0.0000	0.5617	0.5245	0.8812
0.0000	0.7154	0.7483	1.1336
0.0000	0.4223	0.3752	0.6199
0.0000	0.5570	0.5398	1.1310
0.0000	0.7455	0.6730	0.9722

Figure 4B

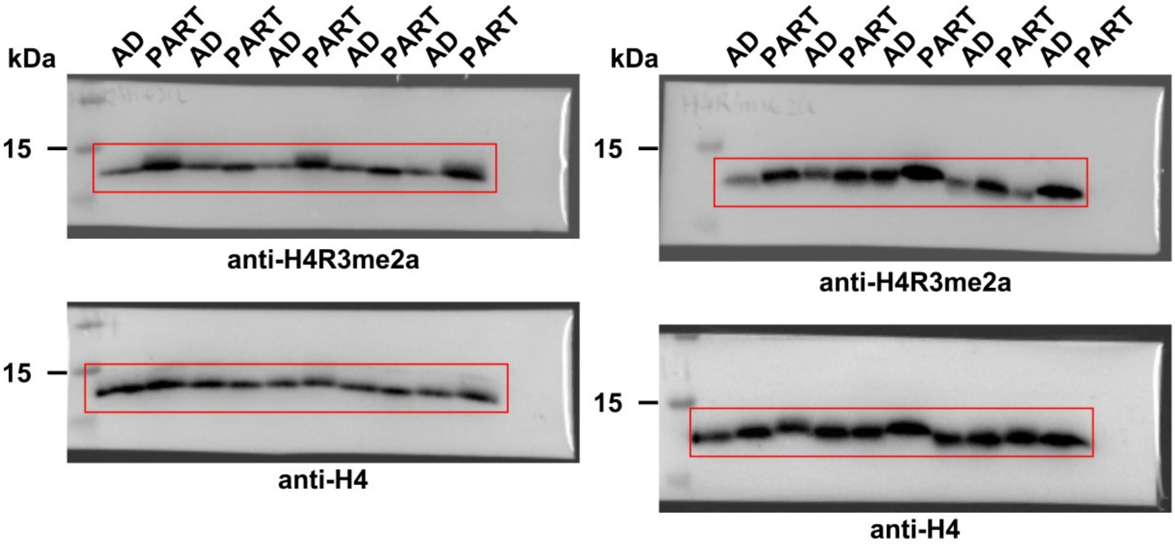

Figure 4B

H4R3me2a/H4	
**AD**	**PART**
0.4971	0.9826
0.6224	0.9514
1.1254	1.5967
0.3793	0.8884
0.3797	1.0441
0.4584	1.0676
0.8126	1.1960
0.7910	1.4146
0.8718	1.2756
0.7825	1.4177

Figure 4D

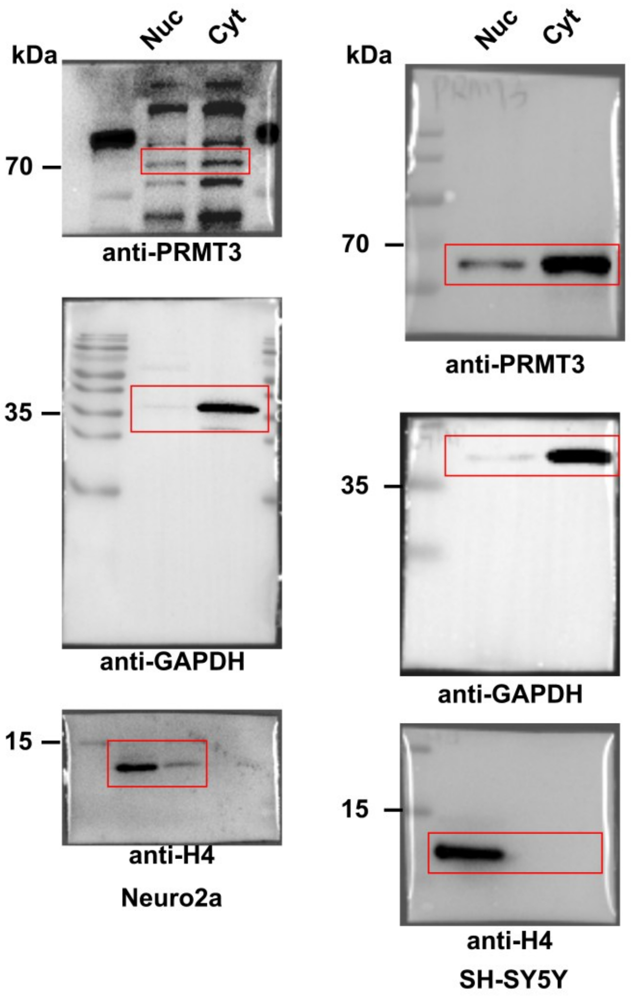

Figure 4F

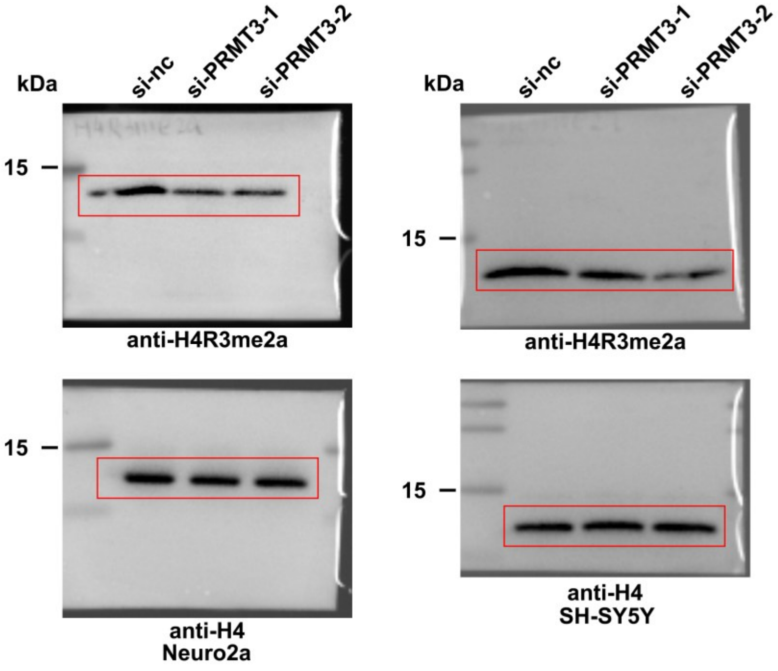

Figure 4F Neuro2a

H4R3me2a/H4		
**si-nc**	**si-PRMT3-1**	**si-PRMT3-2**
1.0481	0.6221	0.5976
0.9769	0.6050	0.5278
1.0968	0.6196	0.6047

Figure 4F SH-SY5Y

H4R3me2a/H4		
**si-nc**	**si-PRMT3-1**	**si-PRMT3-2**
1.2396	0.7212	0.4407
1.2771	0.6959	0.4855
1.2228	0.6731	0.4827

Figure 4G

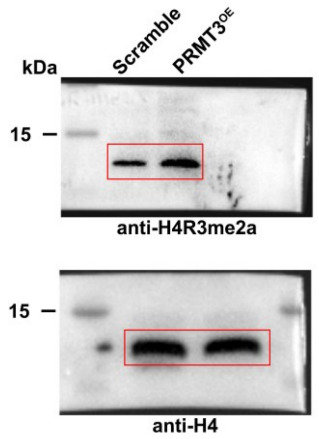

Figure 4G

H4R3me2a/H4	
**Scramble**	**PRMT3^{OE}**
0.5398	1.0171
0.5304	1.0377
0.5171	1.0958

Figure 4H

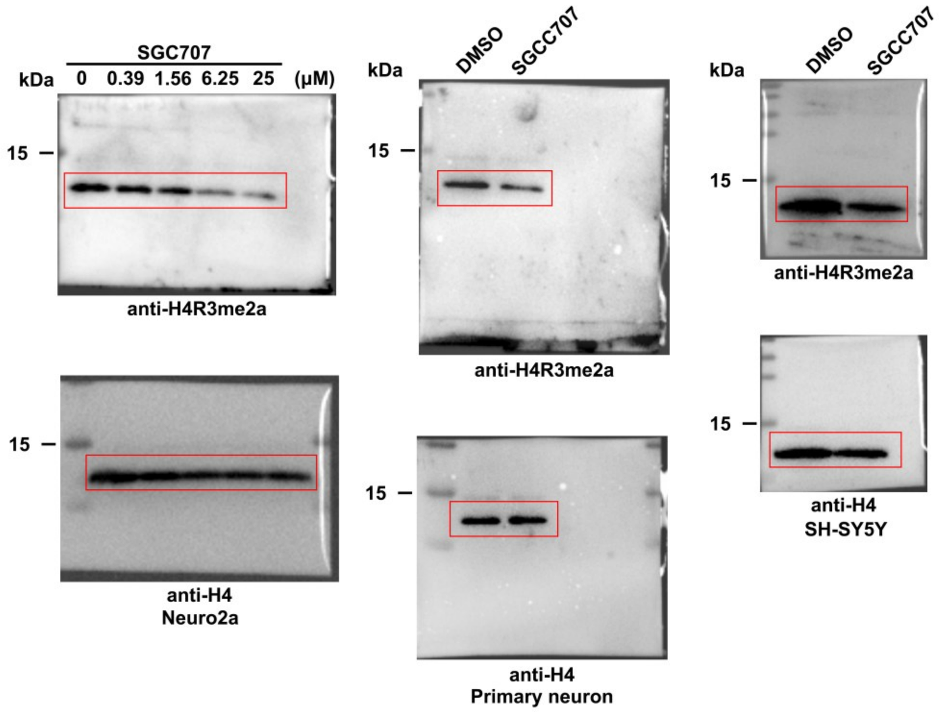

Figure 4H (Neuro2a)

H4R3me2a/H4				
**0µM**	**0.39µM**	**1.56µM**	**6.25µM**	**25µM**
0.8380	0.7957	0.8020	0.4391	0.3361
0.8376	0.7707	0.7145	0.5125	0.3958
0.8815	0.7202	0.6893	0.4661	0.3849

Figure 4H (primary neuron)

H4R3me2a/H4	
**DMSO**	**SGC707**
1.1212	0.6694
1.1313	0.6694
1.0930	0.7273

Figure 4H (SH-SY5Y)

H4R3me2a/H4	
**DMSO**	**SGC707**
0.9550	0.7672
0.9181	0.6763
0.9240	0.6597

Figure S3E

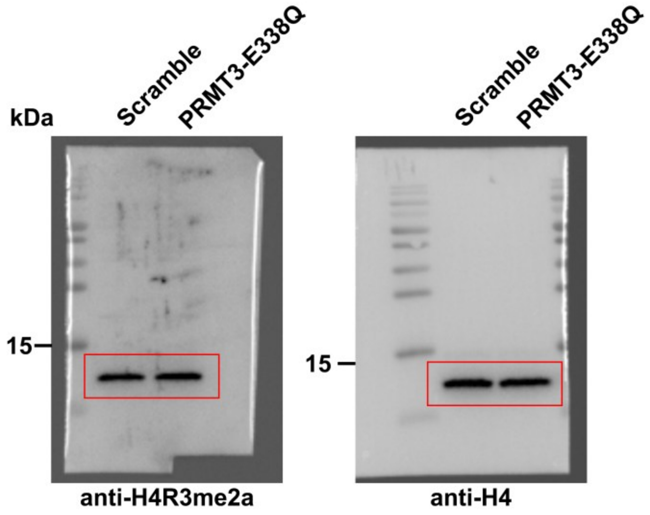

Figure S3E

H4R3me2a/H4	
**Scramble**	**PRMT3-E338Q**
0.7318	0.7436
0.7390	0.7620
0.7243	0.7432

Figure S3F

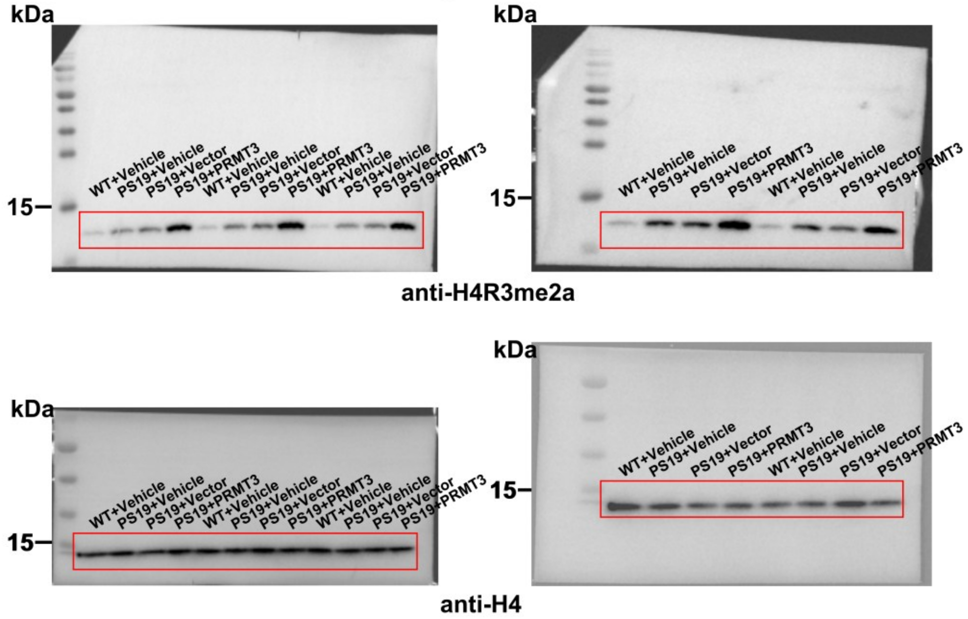

Figure S3F

H4R3me2a/H4			
**WT+Vehicle**	**PS19+Vehicle**	**PS19+Vector**	**PS19+PRMT3**
0.1207	0.3926	0.4142	0.9401
0.1274	0.3678	0.4167	1.1464
0.1093	0.3263	0.4023	1.1814
0.1762	0.7811	1.0201	1.7736
0.2768	0.9048	0.5337	1.5709

Figure 5F

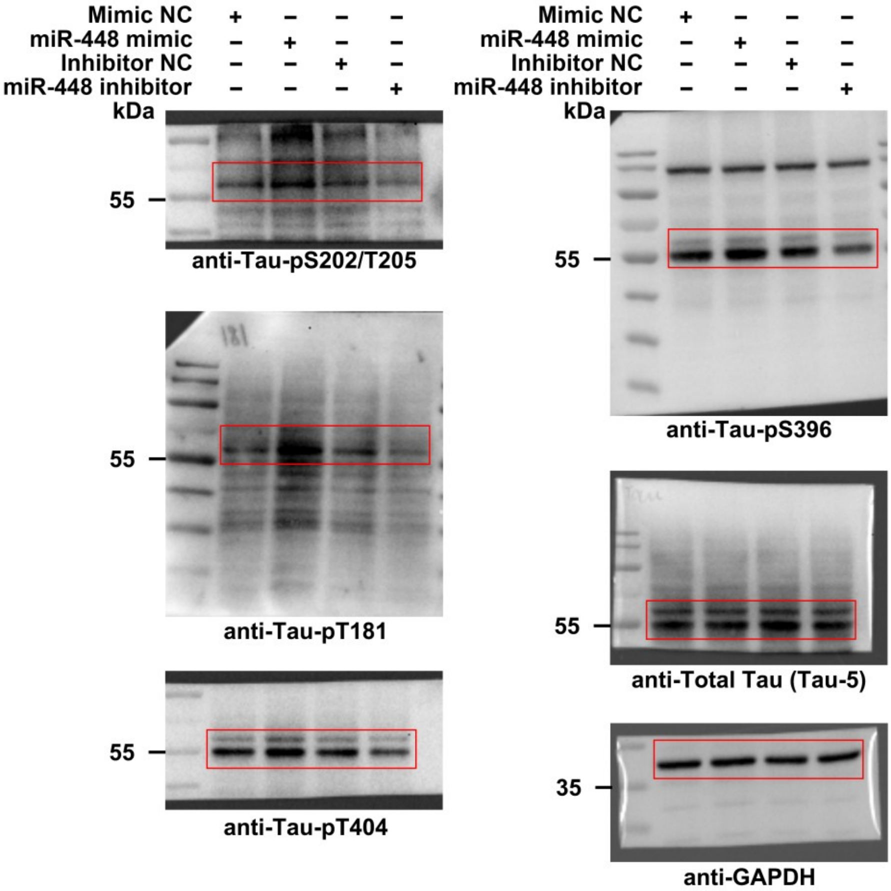

Figure 5F

p-tau S202 T205/total tau			
**Mimic NC**	**miR-448 mimic**	**Inhibitor NC**	**miR-448 inhibitor**
0.7116	1.1382	0.7071	0.5609
0.7862	1.1187	0.7399	0.6116
0.7541	1.1105	0.7201	0.5560
p-tau pT181/total tau			
**Mimic NC**	**miR-448 mimic**	**Inhibitor NC**	**miR-448 inhibitor**
0.4143	0.5990	0.3863	0.2680
0.4200	0.5275	0.3821	0.2603
0.4505	0.5789	0.4676	0.3153
p-tau pT404/total tau			
**Mimic NC**	**miR-448 mimic**	**Inhibitor NC**	**miR-448 inhibitor**
0.7814	0.9216	0.7361	0.5816
0.7581	0.9049	0.7231	0.5892
0.7246	0.8819	0.7404	0.6131
p-tau pT396/total tau			
**Mimic NC**	**miR-448 mimic**	**Inhibitor NC**	**miR-448 inhibitor**
1.3191	1.6447	1.1146	0.7927
1.2773	1.4308	1.1152	0.8364
1.3029	1.5640	1.2261	0.8200

Figure 5H

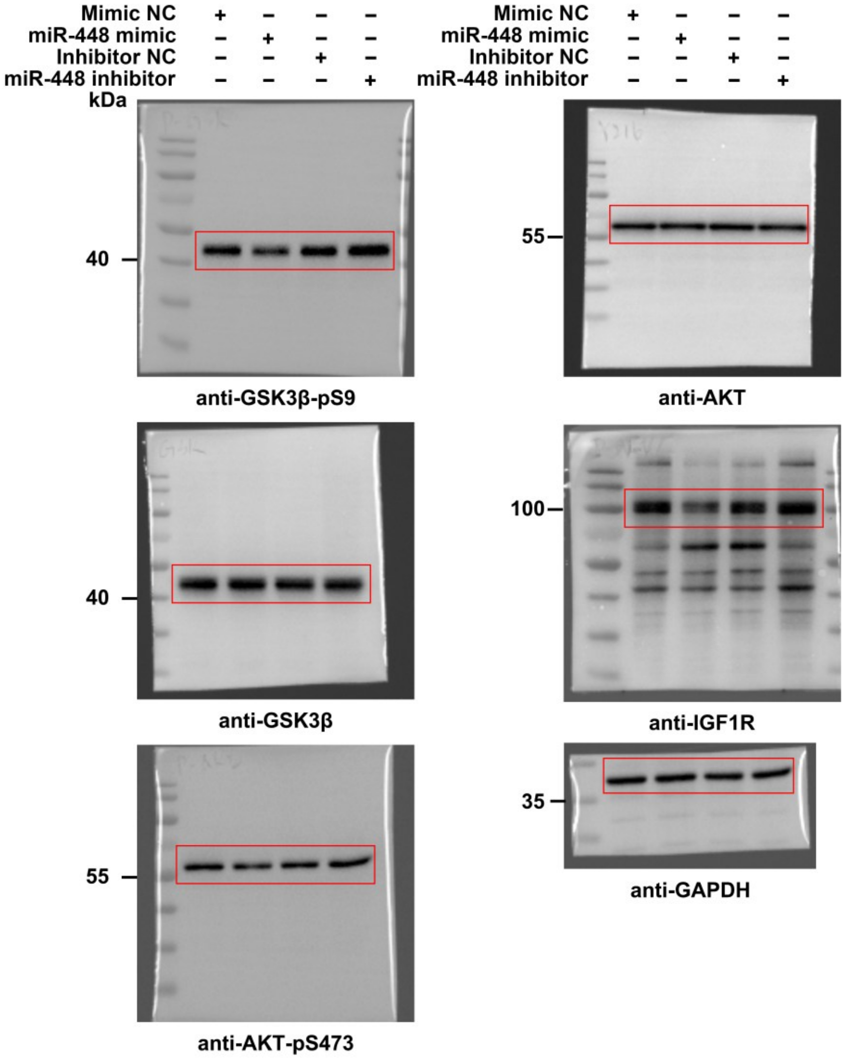

Figure 5H

p-GSK3β pS9/GSK3β			
**Mimic NC**	**miR-448 mimic**	**Inhibitor NC**	**miR-448 inhibitor**
0.8784	0.6537	0.9120	1.1291
0.9109	0.6817	0.9568	1.1772
0.8880	0.6798	0.9203	1.1474
p-AKT pS473/AKT			
**Mimic NC**	**miR-448 mimic**	**Inhibitor NC**	**miR-448 inhibitor**
0.9182	0.6827	0.7686	1.0079
0.9094	0.6578	0.7681	0.9871
0.8082	0.6524	0.7560	0.9801
IGF1R/GAPDH			
**Mimic NC**	**miR-448 mimic**	**Inhibitor NC**	**miR-448 inhibitor**
1.3106	0.6910	0.9473	1.3208
1.4876	0.7465	1.0660	1.5226
1.6144	0.9204	1.2410	1.7144

Figure 5J

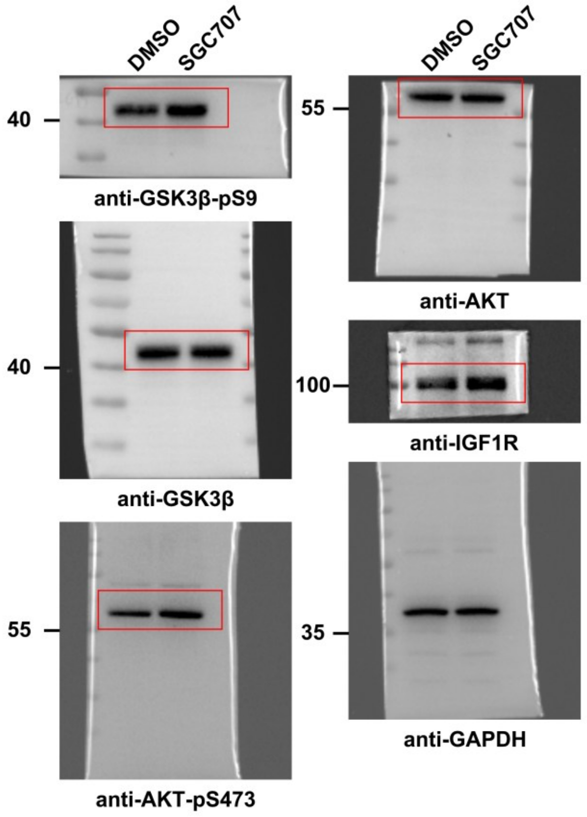

Figure 5J

p-GSK3β pS9/GSK3β	
**DMSO**	**SGC707**
0.8503	1.0016
0.8679	0.9342
0.8390	0.9838
p-AKT pS473/AKT	
**DMSO**	**SGC707**
0.7819	0.9659
0.7242	0.9069
0.8084	1.0408
IGF1R/GAPDH	
**DMSO**	**SGC707**
1.2865	1.5430
1.2677	1.5217
1.2645	1.4803

Figure 5K

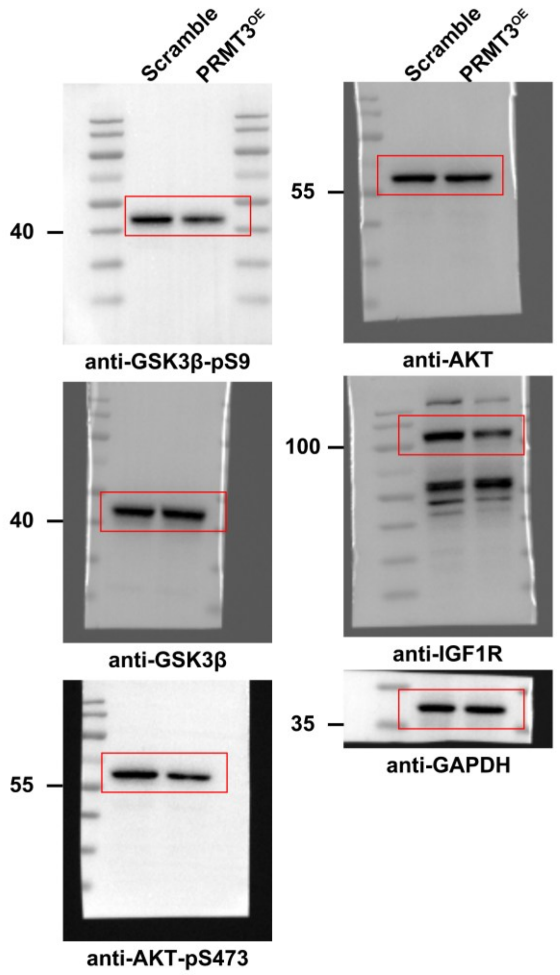

Figure 5K

p-GSK3β pS9/GSK3β	
**Scramble**	**PRMT3^{OE}**
1.0339	0.7573
1.0155	0.7256
0.9495	0.6762
p-AKT pS473/AKT	
**Scramble**	**PRMT3^{OE}**
1.2457	0.9246
1.2095	0.8624
1.3482	1.0024
IGF1R/GAPDH	
**Scramble**	**PRMT3^{OE}**
1.2164	0.6841
1.2792	0.6911
1.1485	0.7075

Figure 5L

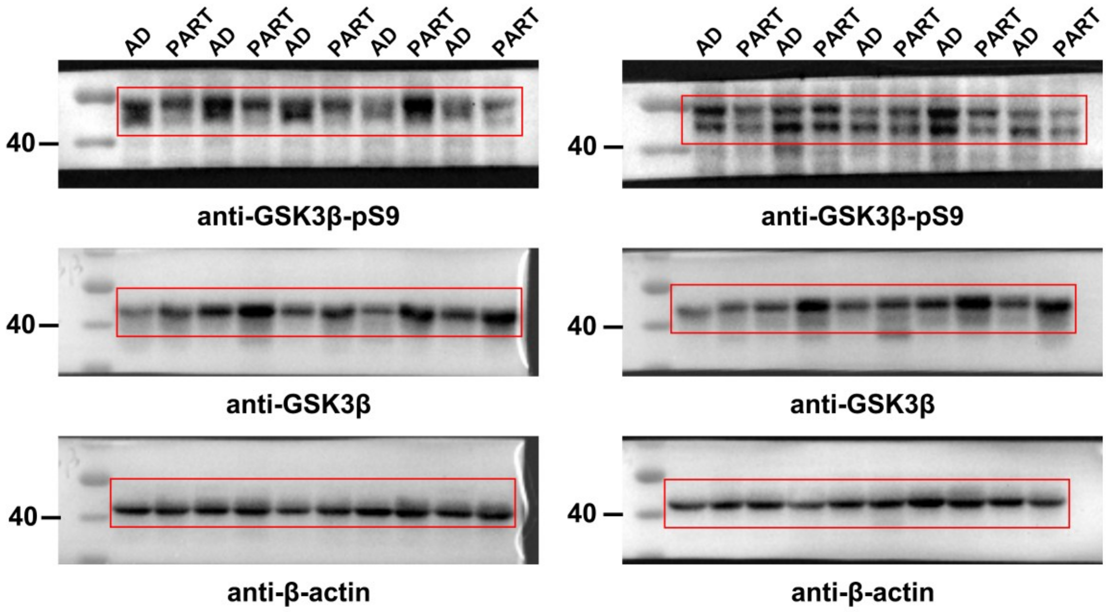

Figure 5L

p-GSK3β pS9/GSK3β	
**AD**	**PART**
2.1502	1.3196
1.7122	0.9317
1.3170	1.1947
1.5322	0.6856
1.3783	0.5850
2.2931	1.1670
1.3388	0.7421
1.5886	0.9979
1.3356	1.2102
0.8902	0.5499

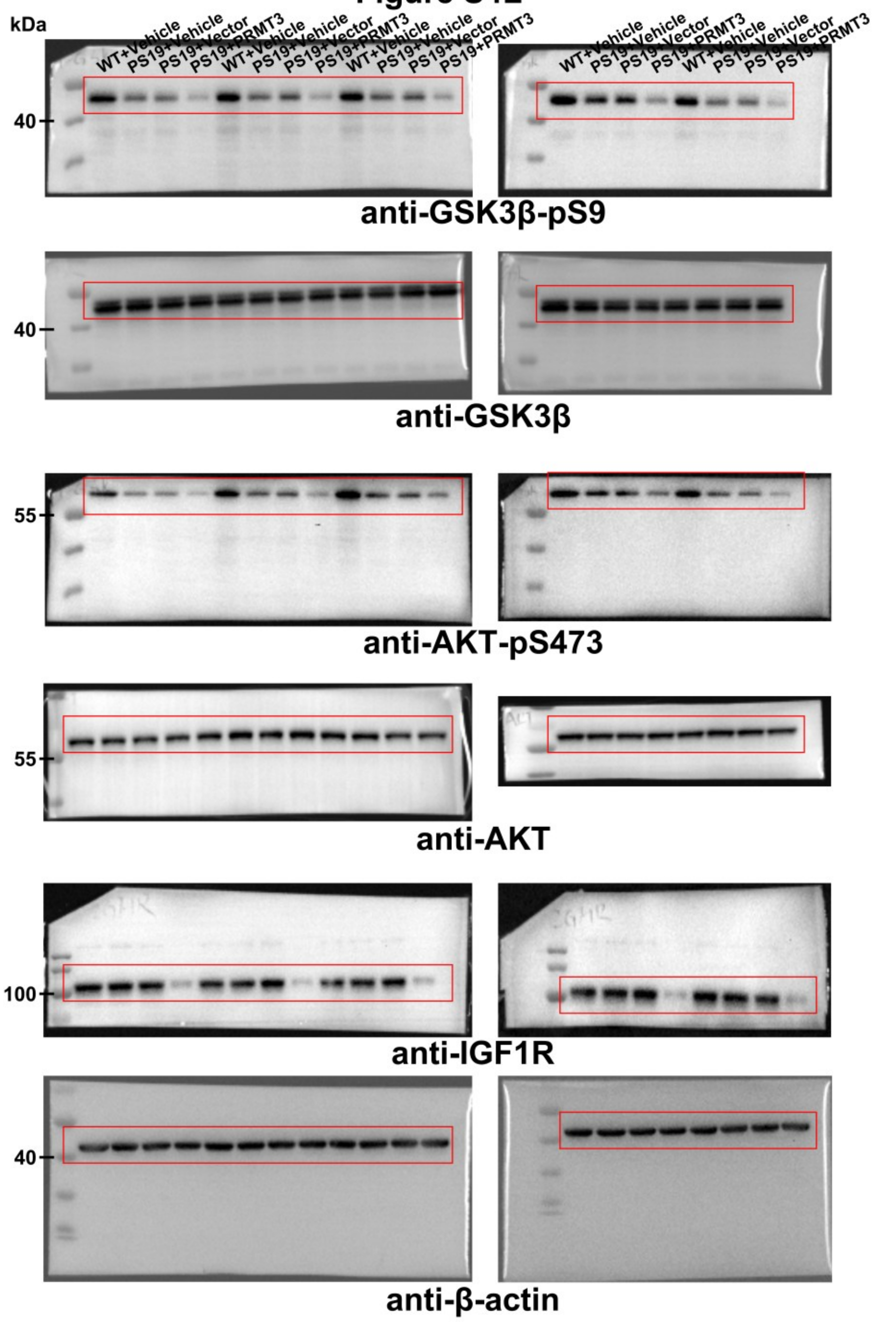

Figure S4L

p-GSK3β pS9/GSK3β			
**WT+Vehicle**	**PS19+Vehicle**	**PS19+Vector**	**PS19+PRMT3**
0.8869	0.4519	0.4501	0.2125
1.0367	0.5200	0.5762	0.2593
1.0221	0.5227	0.5155	0.1975
1.0066	0.7252	0.7461	0.3863
1.0012	0.4908	0.4956	0.1848

p-AKT pS473/AKT			
**WT+Vehicle**	**PS19+Vehicle**	**PS19+Vector**	**PS19+PRMT3**
0.8865	0.4449	0.4436	0.2615
1.1416	0.5307	0.7242	0.3192
1.1591	0.6020	0.5450	0.3886

1.0980	0.7248	0.6567	0.3872
0.9242	0.4199	0.4095	0.2183

IGF1R/GAPDH			
**WT+Vehicle**	**PS19+Vehicle**	**PS19+Vector**	**PS19+PRMT3**
1.2911	1.0299	0.9744	0.2843
0.8025	0.7941	0.9778	0.1933
0.7604	0.8557	1.0820	0.2817
0.9941	0.9585	1.0702	0.3113
1.0568	1.0819	0.9258	0.2877

Figure S4O

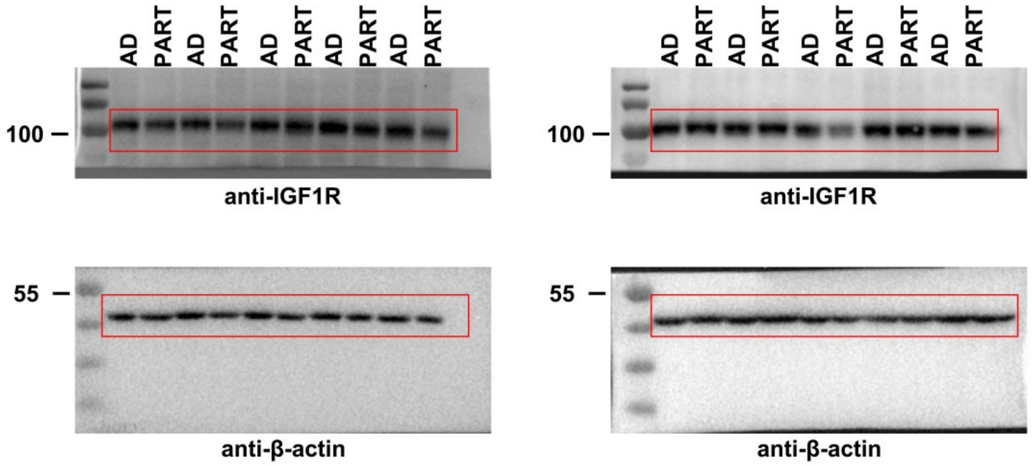

Figure S4O

IGF1R/β-actin	
**AD**	**PART**
0.9700	0.9357
0.8209	0.8402
1.0230	0.7612
1.0958	1.0717
0.8798	0.7090
0.7839	0.6981
0.7829	0.7120
0.8004	0.8148
0.8875	0.9206
0.7249	0.8116

Figure S5A

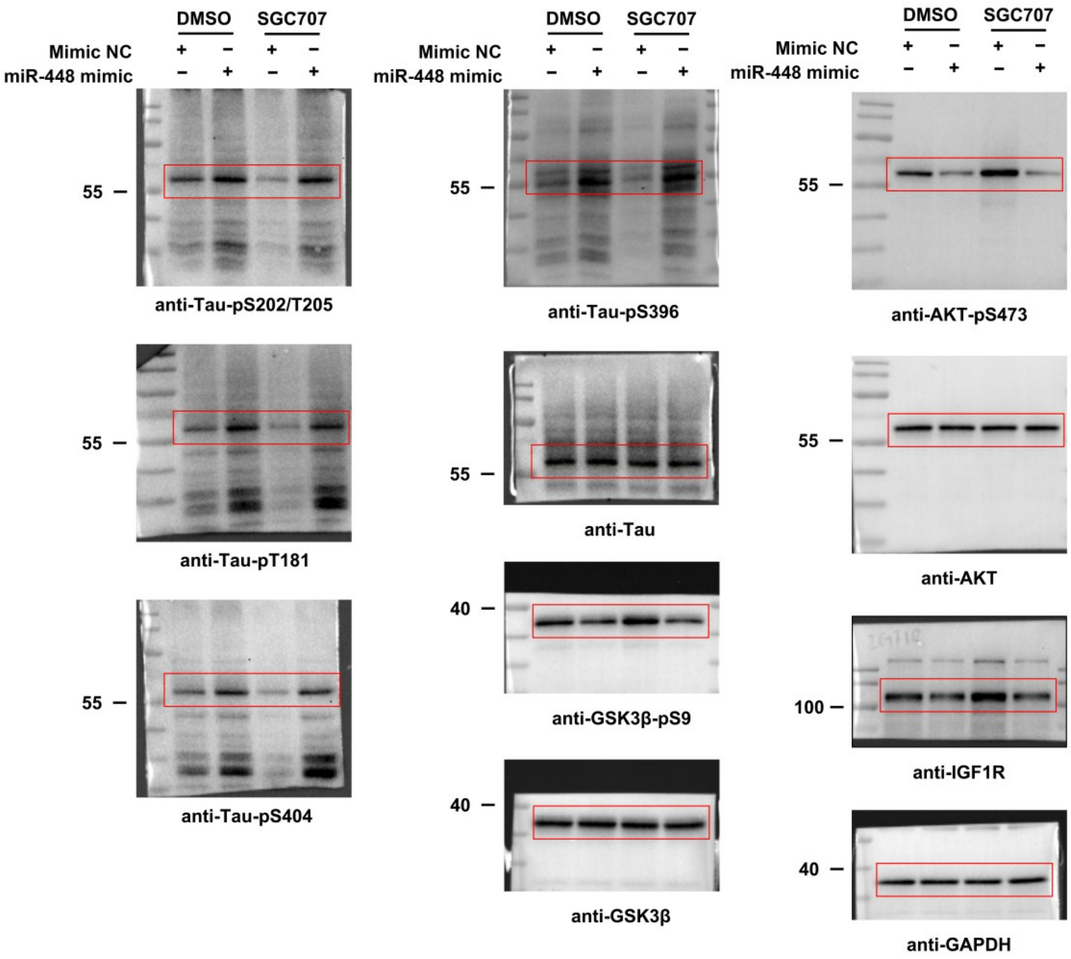
DMSO
SGC707
Mimic NC
miR-448 mimic
55
anti-Tau-pS202/T205
anti-Tau-pT181
anti-Tau-pS404
anti-Tau-pS396
anti-Tau
40
anti-GSK3β-pS9
anti-GSK3β
anti-AKT-pS473
anti-AKT
100
anti-IGF1R
anti-GAPDH

Figure S5B

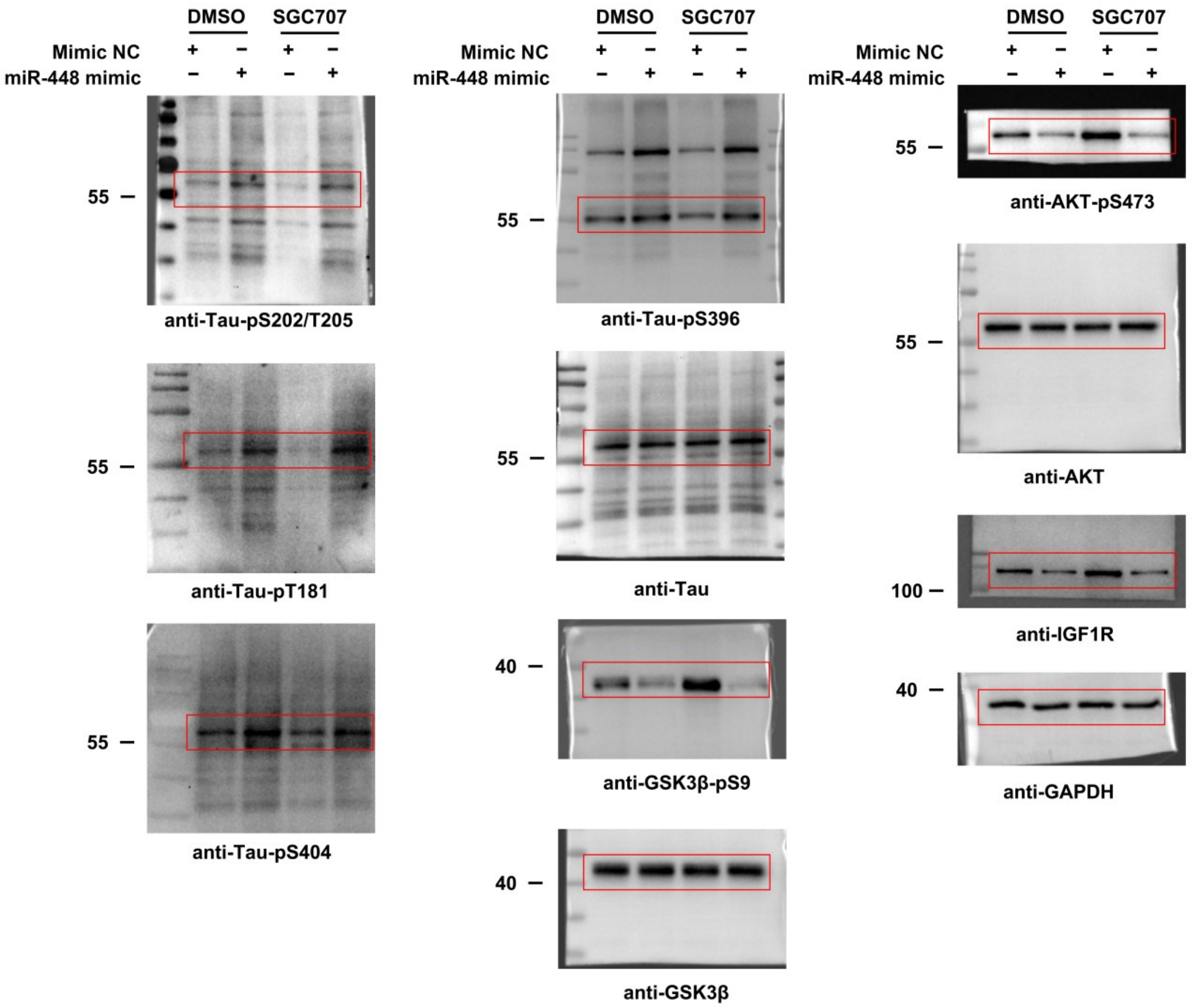

DMSO
SGC707
Mimic NC + − + −
miR-448 mimic − + − +
55
anti-Tau-pS202/T205
55
anti-Tau-pT181
55
anti-Tau-pS404
55
anti-Tau-pS396
55
anti-Tau
40
anti-GSK3β-pS9
40
anti-GSK3β
55
anti-AKT-pS473
55
anti-AKT
100
anti-IGF1R
40
anti-GAPDH

Figure S5D

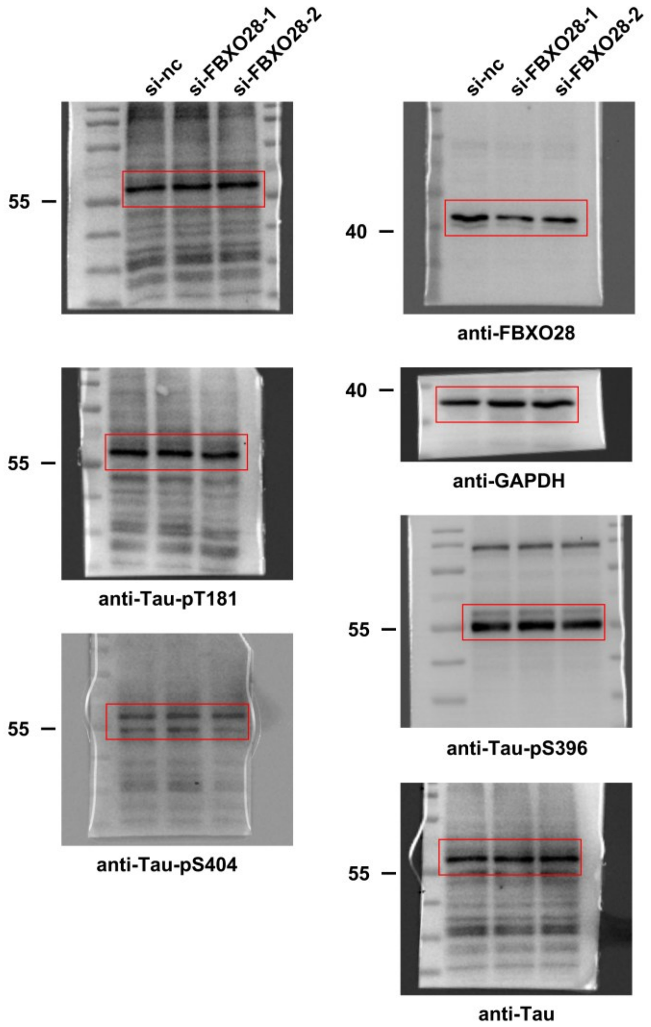

Figure S5E

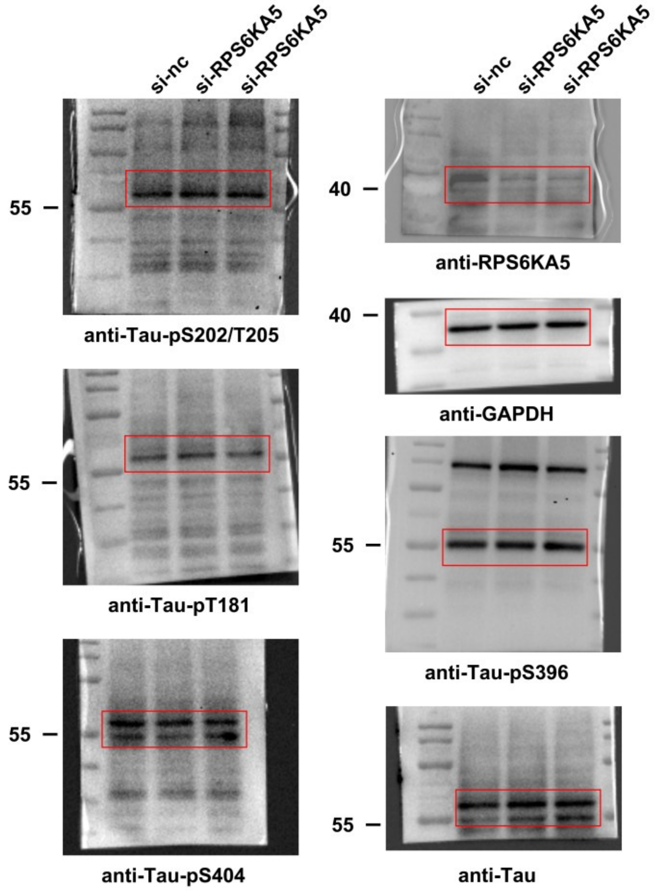

Figure S5F

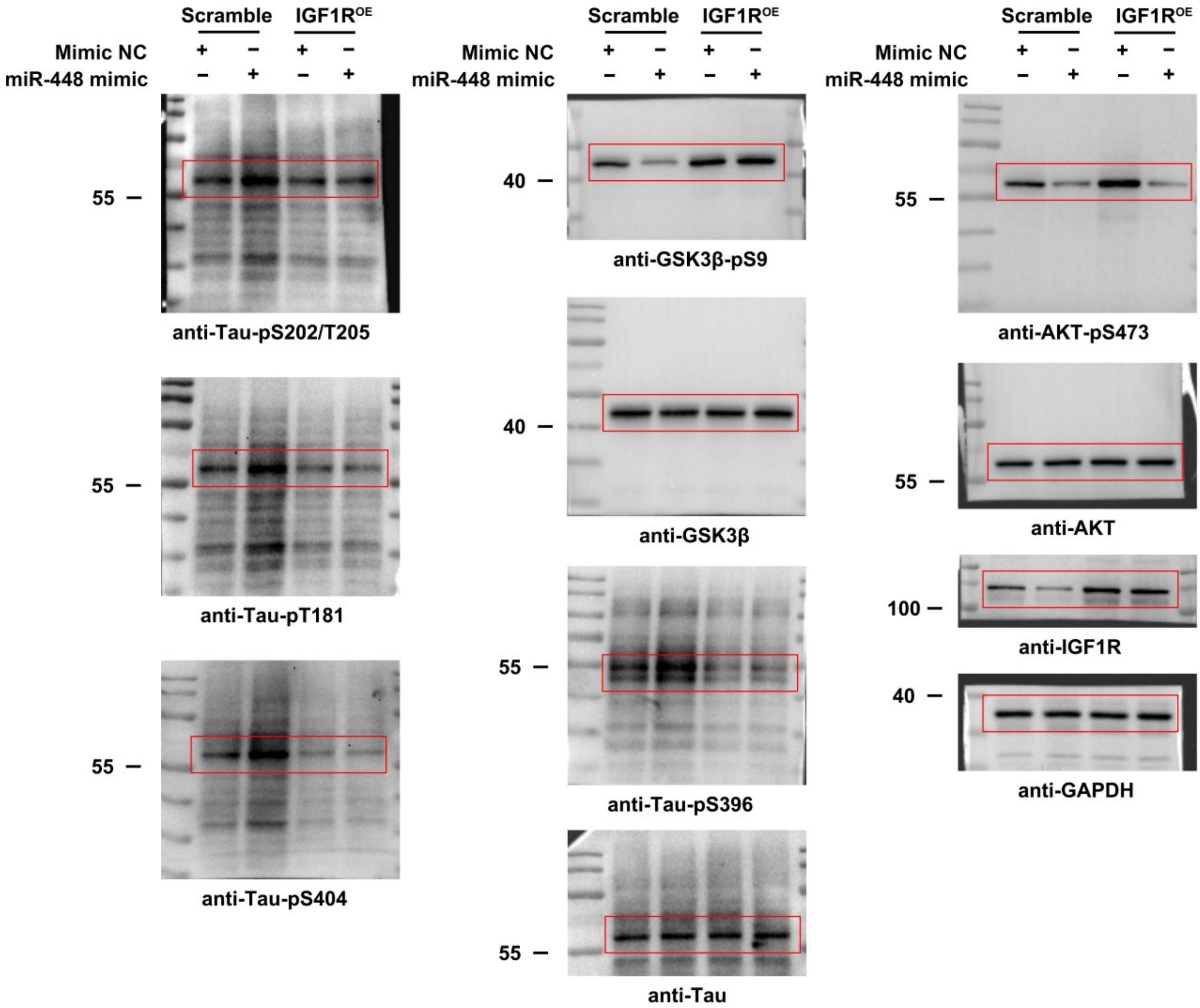
Scramble
IGF1R^OE
Mimic NC
miR-448 mimic
55
anti-Tau-pS202/T205
55
anti-Tau-pT181
55
anti-Tau-pS404
40
anti-GSK3β-pS9
40
anti-GSK3β
55
anti-Tau-pS396
55
anti-Tau
55
anti-AKT-pS473
55
anti-AKT
100
anti-IGF1R
40
anti-GAPDH

Figure S5G

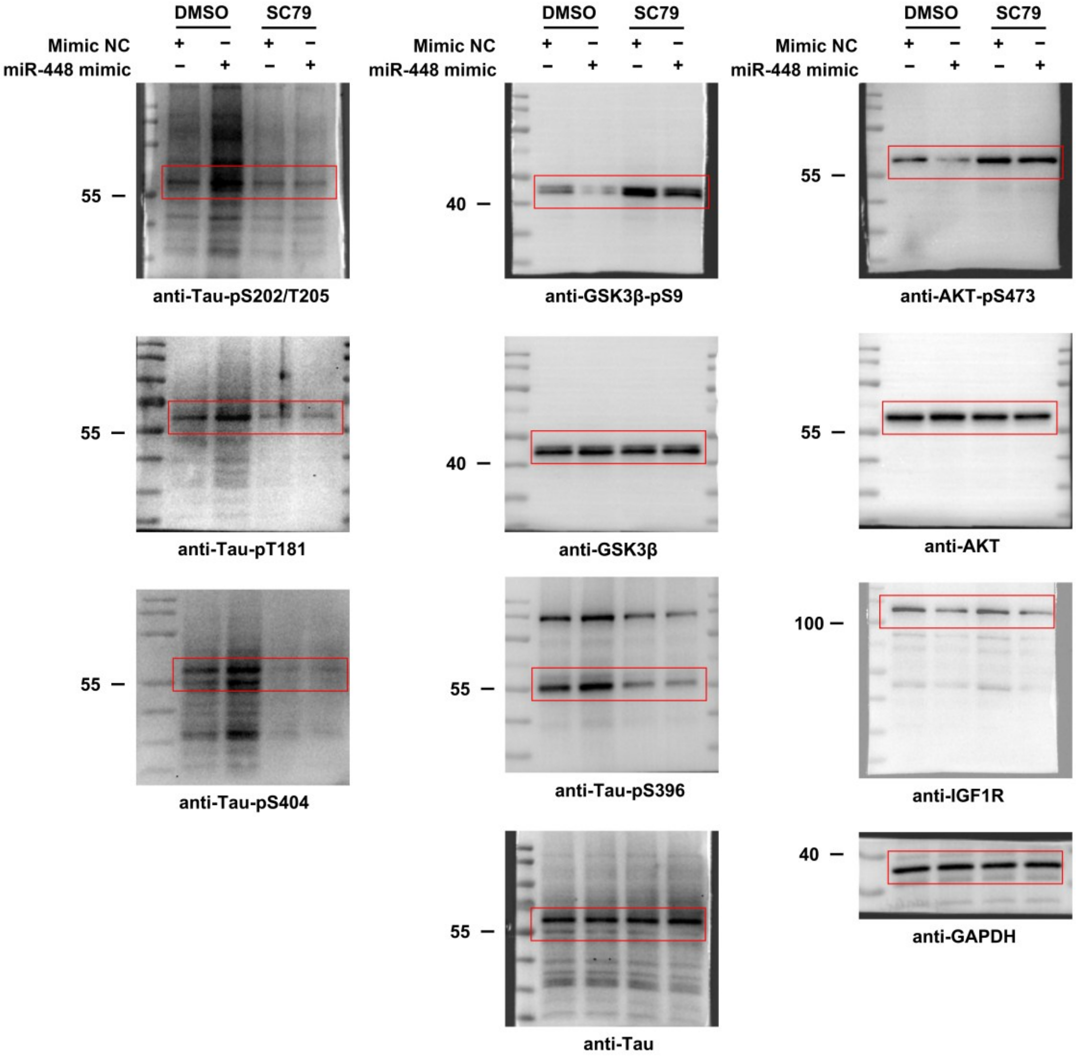
DMSO
SC79
Mimic NC
miR-448 mimic
55
40
100
anti-Tau-pS202/T205
anti-GSK3β-pS9
anti-AKT-pS473
anti-Tau-pT181
anti-GSK3β
anti-AKT
anti-Tau-pS404
anti-Tau-pS396
anti-IGF1R
anti-GAPDH
anti-Tau

Figure 6A

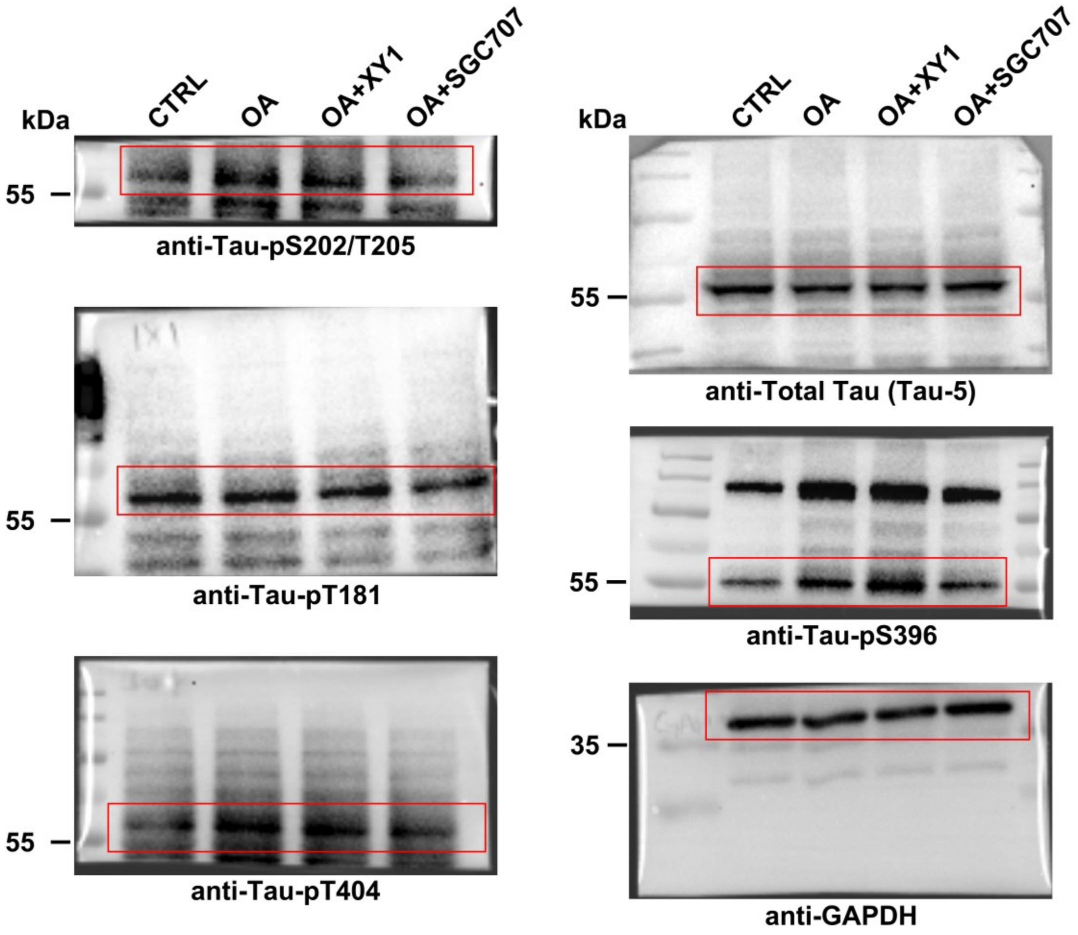

Figure 6A

p-tau S202 T205/total tau			
**CTRL**	**OA**	**OA+XY1**	**OA+SGC707**
0.5724	0.9711	0.9532	0.7397
0.5056	0.9175	0.8950	0.6601
0.5291	0.9996	1.0408	0.6078
p-tau pT181/total tau			
**CTRL**	**OA**	**OA+XY1**	**OA+SGC707**
0.5629	0.9463	0.9042	0.6930
0.5973	0.8342	0.9830	0.7172
0.5863	0.8657	0.8680	0.5975
p-tau pS404/total tau			
**CTRL**	**OA**	**OA+XY1**	**OA+SGC707**
0.6388	1.1233	1.0397	0.7926
0.6115	1.1080	1.1391	0.7333
0.5719	1.0313	1.0286	0.6588
p-tau pS396/total tau			
**CTRL**	**OA**	**OA+XY1**	**OA+SGC707**
0.4327	0.7907	1.0289	0.6512
0.4492	0.8614	1.1244	0.6731
0.4716	0.8929	1.0445	0.7308

Figure 6K

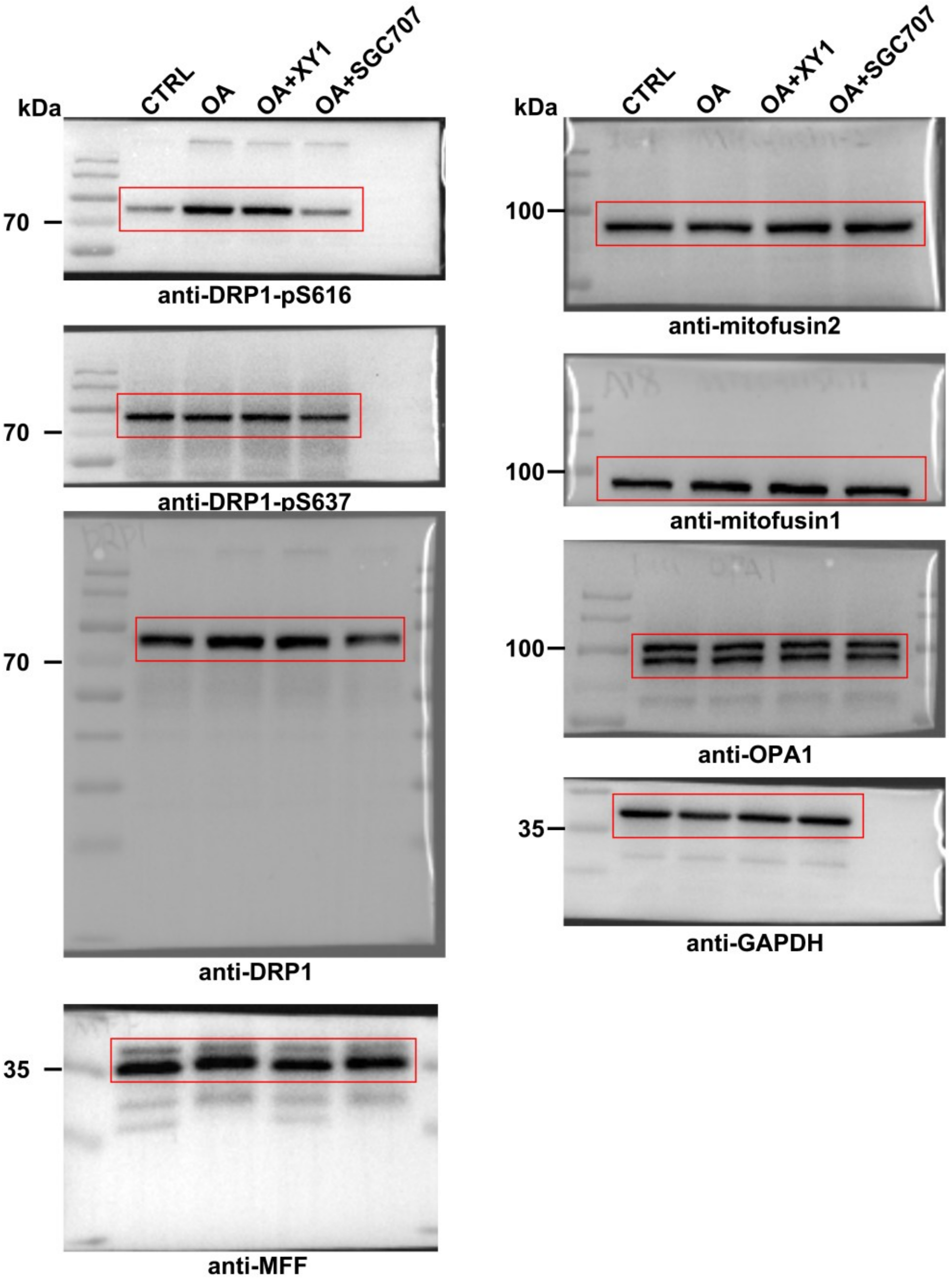

Figure 6K

p-DRP1 pS616/DRP1			
**CTRL**	**OA**	**OA+XY1**	**OA+SGC707**
0.5238	1.0454	1.1200	0.6809
0.5135	1.0786	1.0779	0.7325
0.4621	1.0275	1.1030	0.7186
p-DRP1 pS637/DRP1			
**CTRL**	**OA**	**OA+XY1**	**OA+SGC707**
1.1812	0.8402	1.0082	1.0688
1.0523	0.9424	0.9609	1.0077
1.0089	0.9325	0.9010	1.0447

DRP1/GAPDH			
**CTRL**	**OA**	**OA+XY1**	**OA+SGC707**
0.7550	0.9477	0.8419	0.6204
0.6905	0.8933	0.9100	0.6115
0.7465	0.9394	0.7975	0.6848
MFF/GAPDH			
**CTRL**	**OA**	**OA+XY1**	**OA+SGC707**
0.9228	0.8539	0.6573	0.7383
0.5532	0.6896	0.6879	0.7977
0.7453	0.8161	0.7108	0.7488
Mitofusin2/GAPDH			
**CTRL**	**OA**	**OA+XY1**	**OA+SGC707**
0.8438	0.7735	0.8811	0.9223
0.8850	0.8491	0.9046	0.9377
0.8755	0.8365	0.8538	0.8873
Mitofusin1/GAPDH			
**CTRL**	**OA**	**OA+XY1**	**OA+SGC707**
0.6940	0.8524	0.8939	0.8482
0.7506	0.8232	0.8612	0.7734
0.7069	0.7673	0.8106	0.8034
OPA1/GAPDH			
**CTRL**	**OA**	**OA+XY1**	**OA+SGC707**
0.8777	0.8231	0.7788	0.7780
0.8871	0.8548	0.8391	0.8098
0.9238	0.8411	0.8501	0.8520

Figure S6C

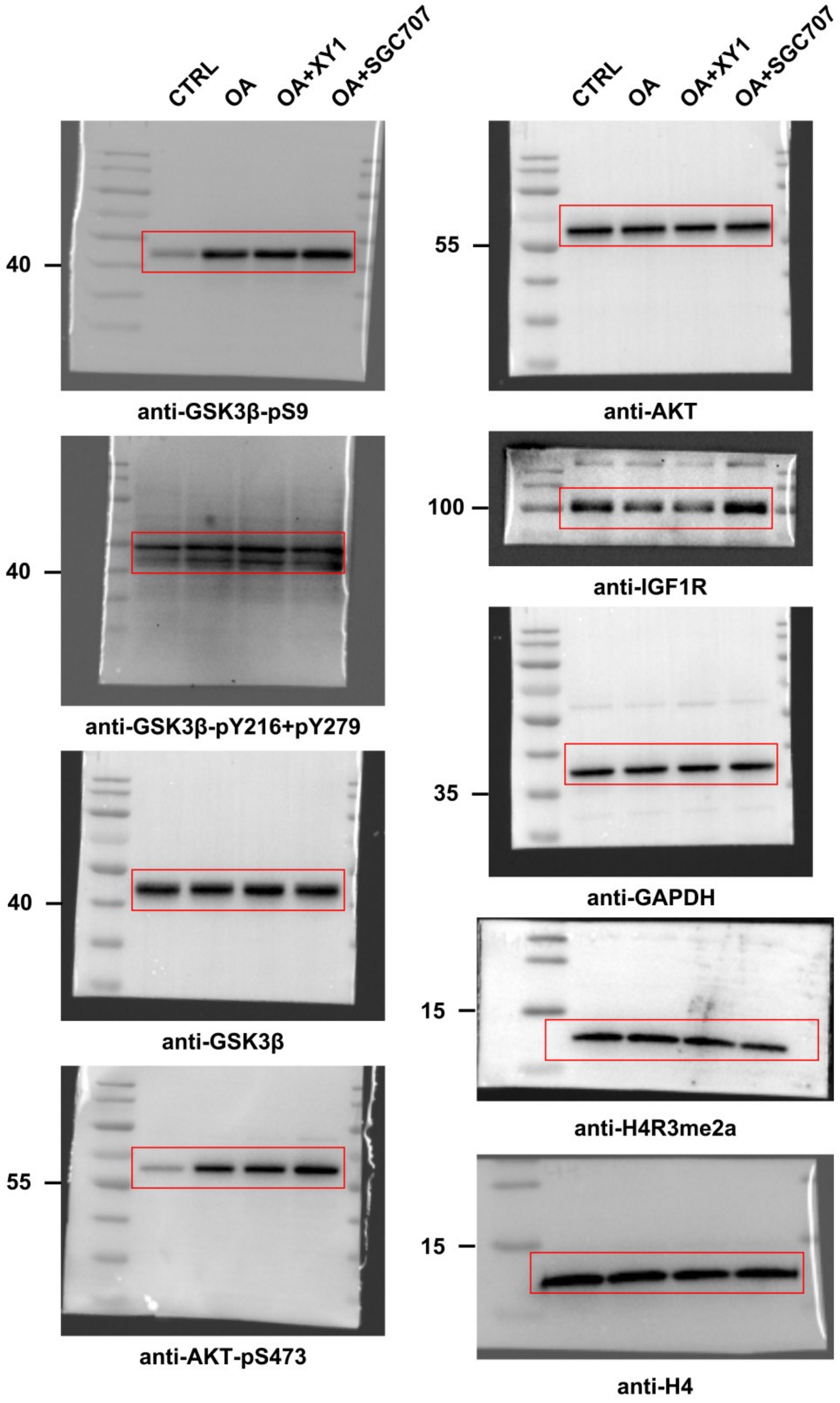

Figure S6C

p-GSK3β pS9/GSK3β			
**WT**	**OA**	**OA+XY1**	**OA+SGC707**
0.2501	0.7472	0.7899	0.9431
0.2675	0.8237	0.8403	1.0303
0.2555	0.8028	0.7543	0.9731

p-GSK3β pY216 pY279/GSK3β			
**CTRL**	**OA**	**OA+XY1**	**OA+SGC707**
0.2871	0.3515	0.3867	0.4163
0.2739	0.3965	0.4229	0.4336
0.2433	0.3610	0.4041	0.3702
p-AKT pS473/AKT			
**CTRL**	**OA**	**OA+XY1**	**OA+SGC707**
0.3767	1.1393	1.1222	1.4783
0.3322	0.9677	0.9974	1.3320
0.2988	0.9063	0.8919	1.2394
IGF1R/GAPDH			
**CTRL**	**OA**	**OA+XY1**	**OA+SGC707**
1.2828	0.9871	0.9661	1.5787
1.2249	0.8899	0.9602	1.5168
1.2448	0.8892	0.8135	1.4005
H4R3me2a/H4			
**CTRL**	**OA**	**OA+XY1**	**OA+SGC707**
0.7507	0.9355	0.9198	0.6050
0.7572	0.8958	0.8971	0.5980
0.7150	0.8880	0.8255	0.5481

Figure 7B

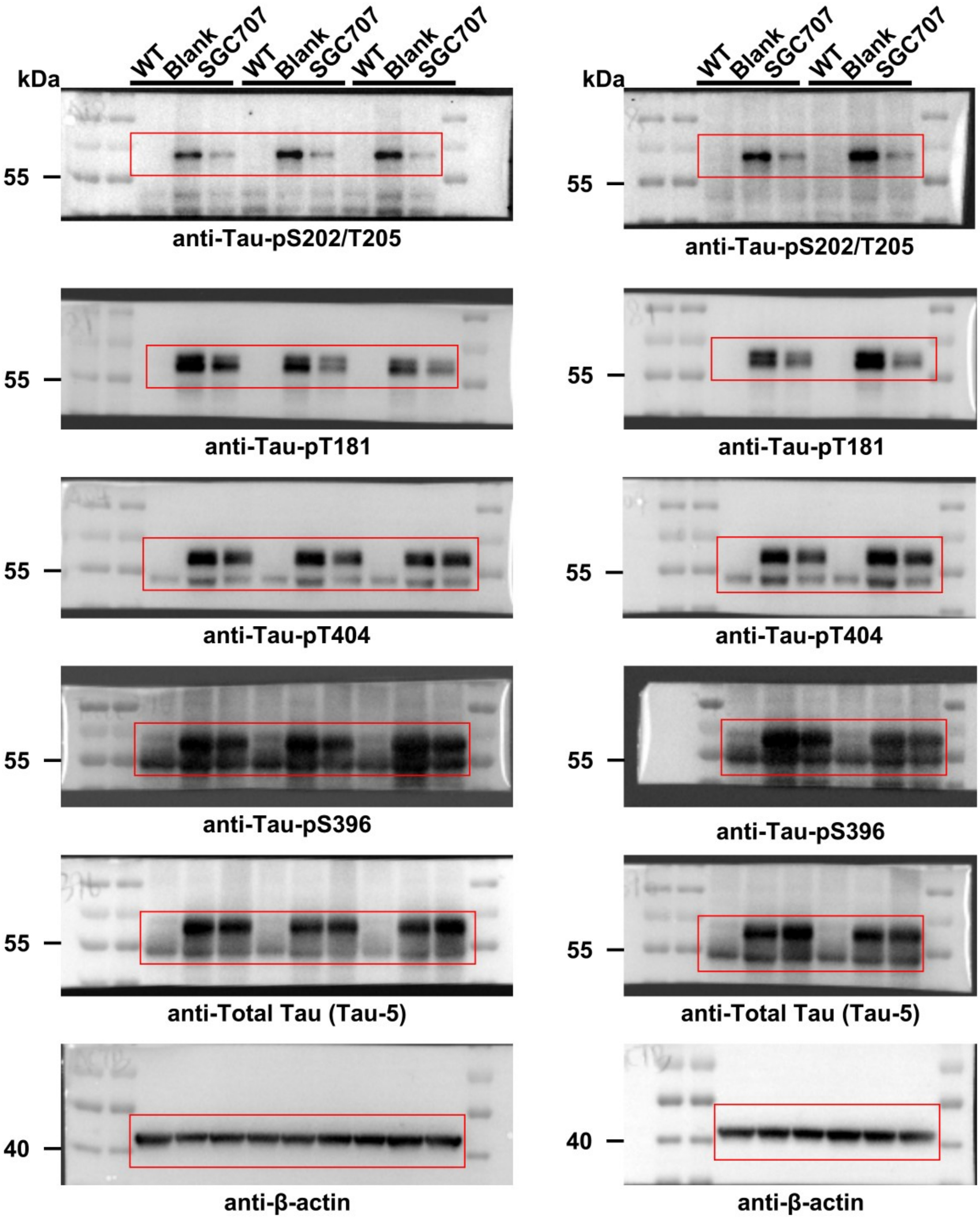

Figure 7B

p-tau S202 T205/total tau		
**WT+Saline**	**PS19+Saline**	**PS19+SGC707**
0.0000	0.3769	0.1440
0.0000	0.5754	0.2033
0.0000	0.5906	0.0966
0.0000	0.4659	0.1435
0.0000	0.5492	0.1737
p-tau pT181/total tau		
**WT+Saline**	**PS19+Saline**	**PS19+SGC707**
0.0000	0.7307	0.6190

0.0000	0.8086	0.4426
0.0000	0.6895	0.3279
0.0000	0.6468	0.3866
0.0000	0.8731	0.4189
p-tau pS404/total tau		
**WT+Saline**	**PS19+Saline**	**PS19+SGC707**
0.0000	0.7262	0.5852
0.0000	0.8821	0.6525
0.0000	0.8635	0.5960
0.0000	0.7408	0.5091
0.0000	0.8091	0.6686
p-tau pS396/total tau		
**WT+Saline**	**PS19+Saline**	**PS19+SGC707**
0.0000	0.8107	0.6990
0.0000	1.0682	0.8131
0.0000	1.3190	0.8438
0.0000	1.1352	0.7941
0.0000	1.0094	0.8180

Figure 7C

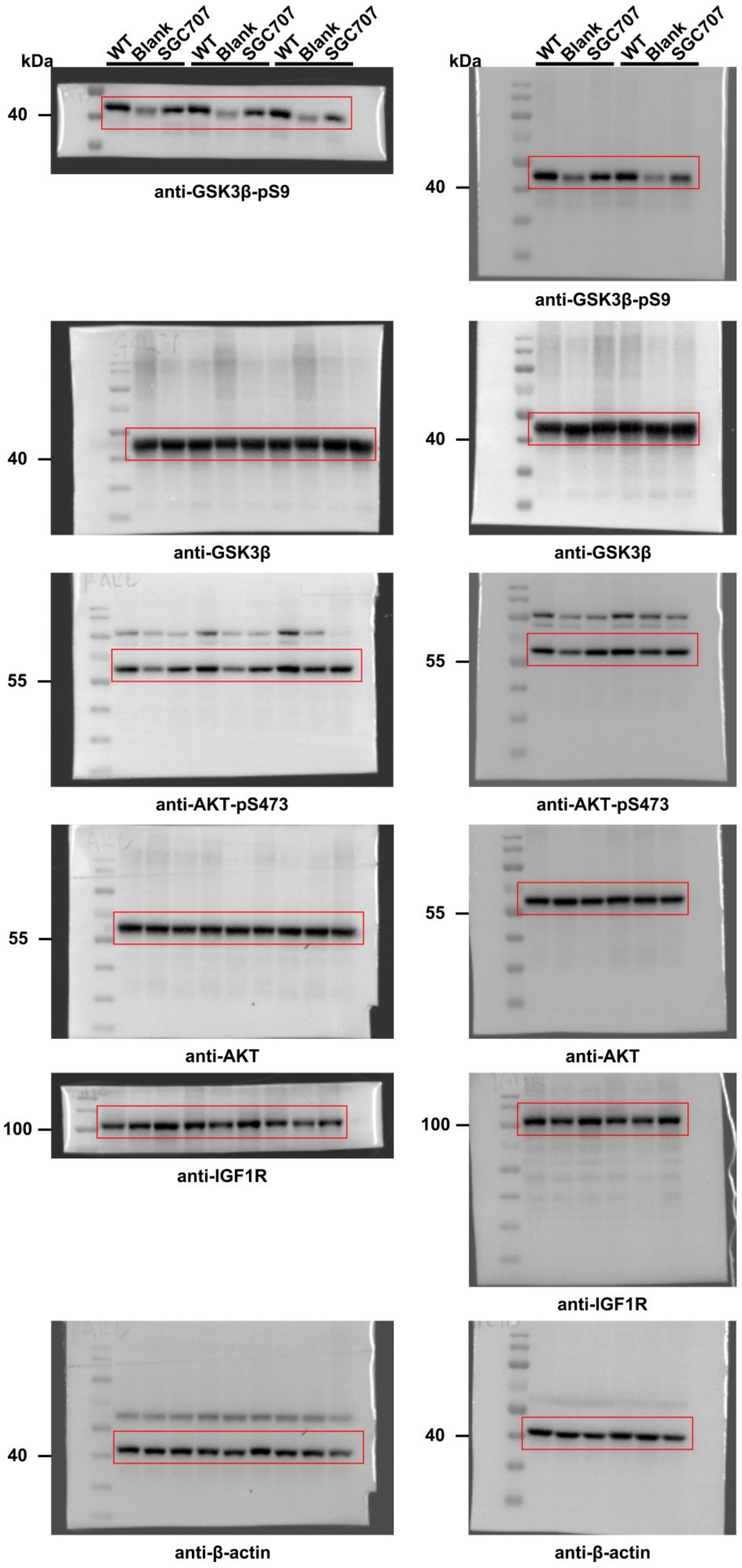

kDa
WT
Blank
SGC707
40
55
100
anti-GSK3β-pS9
anti-GSK3β
anti-AKT-pS473
anti-AKT
anti-IGF1R
anti-β-actin

Figure 7C

p-GSK3β pS9/GSK3β		
**WT+Saline**	**PS19+Saline**	**PS19+SGC707**
0.7410	0.3346	0.5610
0.7404	0.2570	0.5081
0.6945	0.2369	0.3697
0.5671	0.2397	0.4215
0.4833	0.1635	0.2875
p-AKT pS473/AKT		
**WT+Saline**	**PS19+Saline**	**PS19+SGC707**
0.8062	0.5076	0.8051
0.9770	0.4948	0.8522
0.9673	0.7497	0.9586
0.8253	0.5074	0.8930
0.9979	0.7372	0.8806
IGF1R/β-actin		
**WT+Saline**	**PS19+Saline**	**PS19+SGC707**
0.7694	0.8853	0.9774
1.0163	0.9649	0.9532
0.8072	0.7588	1.2600
0.8410	0.7886	1.0937
0.7721	0.7430	1.1478

Figure S7A

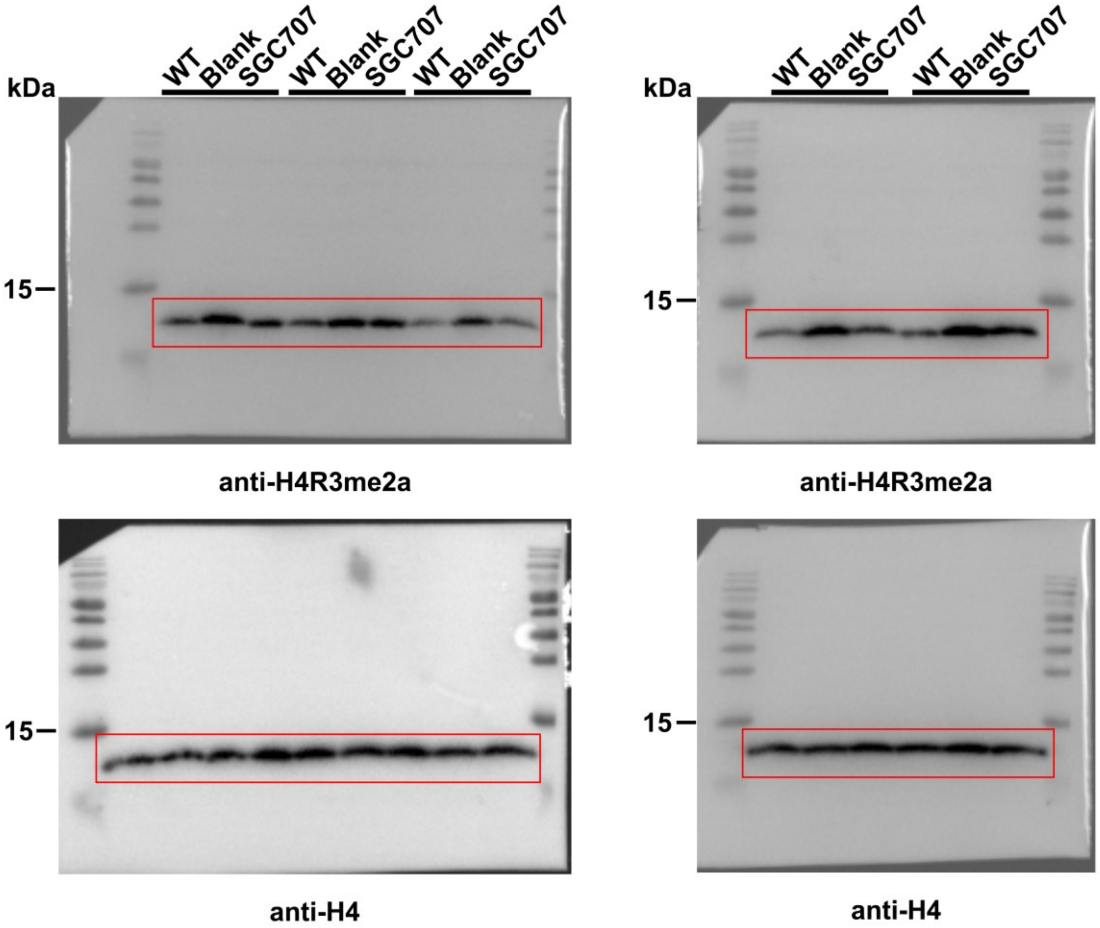

Figure S7A

H4R3me2a/H4		
**WT+Saline**	**PS19+Saline**	**PS19+SGC707**
0.6295	1.2064	0.8214
0.5487	0.9035	0.8024
0.3433	0.7752	0.4030
0.4654	1.1183	0.6541
0.5202	1.0170	0.9187
